# Real-time pure shift ^15^N HSQC of proteins: a real improvement in resolution and sensitivity

**DOI:** 10.1007/s10858-015-9913-z

**Published:** 2015-03-04

**Authors:** Peter Kiraly, Ralph W. Adams, Liladhar Paudel, Mohammadali Foroozandeh, Juan A. Aguilar, István Timári, Matthew J. Cliff, Mathias Nilsson, Péter Sándor, Gyula Batta, Jonathan P. Waltho, Katalin E. Kövér, Gareth A. Morris

**Affiliations:** 1 School of Chemistry, University of Manchester, Oxford Road, Manchester, M13 9PL UK; 2 Department of Anesthesiology and Pain Medicine, Mitochondria and Metabolism Center, University of Washington, 850 Republican St, Seattle, WA 98109 USA; 3 Department of Chemistry, Durham University, South Road, Durham, DH1 3LE UK; 4 Department of Inorganic and Analytical Chemistry, University of Debrecen, Egyetem tér 1, Debrecen, 4032 Hungary; 5 Manchester Institute of Biotechnology, University of Manchester, 131 Princess Street, Manchester, M1 7DN UK; 6 Agilent Technologies R&D and Marketing GmbH & Co. KG, Hewlett-Packard Strasse 8, 76337 Waldbronn, Germany; 7 Department of Organic Chemistry, University of Debrecen, Egyetem tér 1, Debrecen, 4032 Hungary

**Keywords:** Pure shift, Real-time, HSQC, Homodecoupling, Protein

## Abstract

Spectral resolution in proton NMR spectroscopy is reduced by the splitting of resonances into multiplets due to the effect of homonuclear scalar couplings. Although these effects are often hidden in protein NMR spectroscopy by low digital resolution and routine apodization, behind the scenes homonuclear scalar couplings increase spectral overcrowding. The possibilities for biomolecular NMR offered by new pure shift NMR methods are illustrated here. Both resolution and sensitivity are improved, without any increase in experiment time. In these experiments, free induction decays are collected in short bursts of data acquisition, with durations short on the timescale of *J*-evolution, interspersed with suitable refocusing elements. The net effect is real-time (*t*_2_) broadband homodecoupling, suppressing the multiplet structure caused by proton–proton interactions. The key feature of the refocusing elements is that they discriminate between the resonances of active (observed) and passive (coupling partner) spins. This can be achieved either by using band-selective refocusing or by the BIRD element, in both cases accompanied by a nonselective 180° proton pulse. The latter method selects the active spins based on their one-bond heteronuclear *J*-coupling to ^15^N, while the former selects a region of the ^1^H spectrum. Several novel pure shift experiments are presented, and the improvements in resolution and sensitivity they provide are evaluated for representative samples: the N-terminal domain of PGK; ubiquitin; and two mutants of the small antifungal protein PAF. These new experiments, delivering improved sensitivity and resolution, have the potential to replace the current standard HSQC experiments.

**Electronic supplementary material:**

The online version of this article (doi:10.1007/s10858-015-9913-z) contains supplementary material, which is available to authorized users.

## Introduction

To understand relationships between biological structure and function, we need tools for the study of complex systems, such as proteins and oligonucleotides, that have large numbers of constitutionally very similar elements. NMR methods provide invaluable details about both structure and dynamics at the atomic level (Tollinger et al. 2001), but can be limited by the overlap between resonances from multiple individual atomic sites. Indeed, increasing resolution has been crucial to broadening the scope of biomolecular NMR spectroscopy. The use of very strong magnetic fields and multidimensional experiments is now standard. Recent improvements in non-uniform sampling (Mayzel et al. 2014; Mobli and Hoch 2014) can shorten the overall durations of multidimensional NMR experiments, and can be used to increase resolution in indirect dimensions. Improvements in the basic resolution of standard protein 2D NMR experiments can translate directly into the more complex nD experiments that share building blocks, so improving the resolution of the basic HSQC experiment is a priority. Unfortunately this can generally only be achieved by using higher magnetic fields. While adequate sensitivity is usually available from cryogenically cooled NMR probes at high magnetic fields (Styles and Soffe 1984; Kovacs et al. 2005) spectral resolution in the direct proton dimension remains a fundamental limiting factor in current biomolecular NMR applications.

A range of novel methods have been developed for small molecules in recent years, that reduce the complexity of ^1^H NMR spectra by collapsing the multiplet structure of each resonance into a singlet (Zangger and Sterk 1997; Nilsson and Morris 2007; Aguilar et al. 2010, 2011; Sakhaii et al. 2013; Meyer and Zangger 2014a, b; Castañar et al. 2013a, b, 2014a, b; Morris et al. 2010; Sakhaii et al. 2009; Timári et al. 2014; Reinsperger and Luy 2014; Adams et al. 2014). These are often termed “pure shift” experiments, because they deliver pure chemical shift information without the complication of homonuclear coupling interactions. In the case of small molecules, valuable stereochemical information can often be derived from analysis of proton multiplets (Karplus 1959, 1960). However, such information is not normally required from the HSQC spectra of biomolecules [though there are specialised methods available for obtaining it (Permi 2003)]. Rather, the splitting of proton resonances in HSQC increases spectral complexity and degrades resolution and sensitivity, reducing the value of HSQC and its many derivatives in structural biology. There is thus a strong incentive to design pure shift HSQC analogues that would yield fully decoupled correlation maps, for example a ^1^H–^15^N HSQC spectrum without any splitting due to scalar coupling, showing only the ^1^H and the ^15^N chemical shifts, in the direct (*F*_2_) and the indirect (*F*_1_) dimensions respectively.

Heteronuclear ^1^ *J* couplings are typically very large compared to linewidths, so decoupling them requires rapid spin inversion, and most efficient decoupling schemes use continuous radiofrequency irradiation. In contrast, homonuclear couplings are rarely much more than a factor of 10 larger than linewidths, so in principle it should be possible to decouple them with relatively sparse periodic manipulations of the spin system. Pure shift methods typically acquire short chunks of data of duration ≪1/*J*_HH_ (Zangger and Sterk 1997; Nilsson and Morris 2007; Aguilar et al. 2010) since the evolution of the effects of coupling can be neglected on this timescale. Two types of experiment are in common use: interferogram-based, in which a composite free induction decay is built up from multiple individual chunks of data of duration 1/sw_ps_, each acquired after an evolution period *t*_ps_ that is incremented in steps of 1/sw_ps_; and real-time, in which blocks of data of duration 1/sw_ps_ are acquired one after another in the direct dimension (*t*_2_). The subscript “ps” refers to the pure shift dimension, which is essentially the same as the direct dimension in a real-time experiment, but requires increasing the dimensionality when the interferogram-based strategy is employed. The requirement that each chunk corresponds to a whole number of data points means that sw_ps_ must be an integer submultiple of the direct acquisition spectral width (sw). In each case one of a number of different possible pulse sequence elements can be used to refocus the effects of coupling, either in the middle of *t*_ps_ for an interferogram experiment, or in between acquisition of data chunks in a real-time experiment.

The *J*-refocusing pulse sequence elements used all distinguish between active spins, for which signals are to be recorded, and passive spins, for which the effects of couplings with active spins are to be suppressed. Since only a minority of spins are observed, there is often (although not invariably) a price in sensitivity and/or experiment time to be paid for the decoupling achieved. Increased experiment time is a particular problem in biomolecular research, where multidimensional experiments are the norm, so here real-time methods that acquire a pure shift FID in a single shot (Lupulescu et al. 2012; Meyer and Zangger 2013; Adams 2014; Kakita and Bharatam 2014) are to be preferred. No special data processing is needed for real-time pure shift NMR experiments, and standard hardware may be used provided that it supports windowed acquisition mode.

A variety of different *J*-refocusing elements have been published; each has its own advantages and disadvantages. Here, we compare the performance of BIRD (Bilinear Rotation Decoupling) (Garbow et al. 1982; Aguilar et al. 2011) and BASHD [Band-Selective-HomoDecoupling (Brüschweiler et al. 1988; Krishnamurthy 1997)] with that of the method currently used for homonuclear decoupling during acquisition for proteins, time-shared band-selective irradiation (Hammarström and Otting 1994; Kupče and Wagner 1995; Kooi et al. 1999; Struppe et al. 2013). The ^15^N HSQC experiment was chosen for the comparison because it is among the most important building blocks of modern multidimensional experiments, and is widely used for screening, e.g. in binding studies and for pH and temperature dependence. The specific versions used here are the multiplicity-edited gHSQC (Boyd et al. 1992) and the sensitivity-enhanced gHSQC-SE (Kontaxis et al. 1994; Schleucher et al. 1994), but the conclusions should be general.

**Fig. 1:**
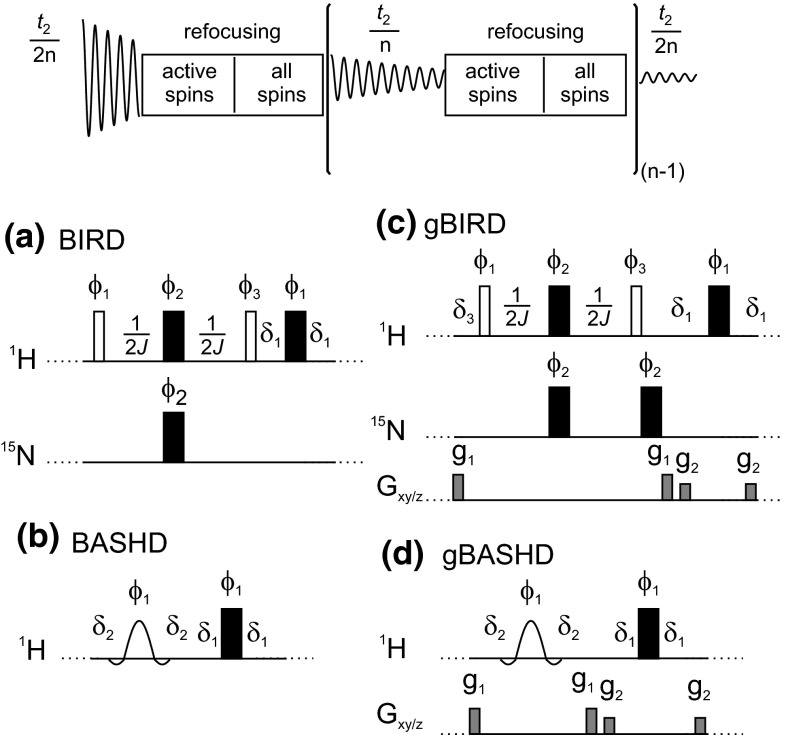
The generic data acquisition scheme for real-time pure shift NMR methods is shown at the *top*. The pulse sequence, preceding data acquisition, was either a standard multiplicity edited HSQC with gradient selection (gHSQC) or a gradient selected HSQC with sensitivity enhancement (gHSQC-SE); only the *J*-refocusing elements (**a**–**d**) are shown here for clarity. The pure shift sequence elements used here were: **a** BIRD; **b** BASHD; **c** BIRD with coherence transfer pathway selection (CTP) gradient pulses denoted by gBIRD; and **d** BASHD with CTP gradient pulses denoted by gBASHD. Delays δ_1_, δ_2_ and δ_3_ were usually set to 20 μs (amplifier and receiver blanking), 50 μs (changing power levels on the transmitter) and 208 μs (time correction for the second nitrogen 180° pulse including the corresponding blanking delays), respectively. See Supplementary Material for all details of the pulse programs. Electronic copies of pulse sequences, parameters, and experimental data are available from http://nmr.chemistry.manchester.ac.uk. The CTP gradient pulses were applied simultaneously on the *x* and *y* channels where triaxial gradients were available. The 16 step phase cycle of gHSQC was extended [Φ_1_ = {x, y}; Φ_2_ = {y, −x}; Φ_3_ = {−x, −y}] to 32 in order to suppress the effects of imperfections of the *J*-refocusing elements. Employing the full phase cycling is not mandatory (the minimum recommended is two steps), but improves the results for sensitivity-limited applications. See Supplementary Figures 7 and 8 for details

We have recently improved the resolution and sensitivity of the gHSQC experiment for small molecules (Paudel et al. 2013) by adding broadband homodecoupling using the BIRD (Garbow et al. 1982) element in a real-time acquisition scheme (see Fig. 1a). The band-selective analogue has recently been used to enhance the resolution of RDC measurements in partially-oriented proteins (Ying et al. 2014). The results of BIRD and BASHD based pure shift methods are directly compared here for the first time. Water suppression is improved by implementing coherence transfer pathway (CTP) selection using pulsed field gradients (Fig. 1c, d); the utility of triaxial gradients (Ferrage et al. 2003; Sarkar et al. 2008) is also explored.

The pure shift ^15^N HSQC methods were tested on: (**1**) L80C mutant of the N-terminal domain of phosphoglycerate kinase in its denaturated state; (**2**) a globular protein (ubiquitin) in its folded state; and (**3**) two mutants of *Penicillium* *chrysogenum* Antifungal Protein (PAF).

## Materials and methods

The pET5 expression vector for wild-type N-PGK comprising residues 1–174 of phosphoglycerate kinase from *G. stearothermophilus* has been described previously (Parker et al. 1996). Mutants were produced by the Quikchange™ method. BL21(DE3) strains of *E. coli* transformed with the appropriate expression vectors were incubated at 37 °C in minimal M9 media with [^15^N]ammonium chloride as the sole nitrogen source, and expression was induced by addition of 1 mM IPTG once an OD_600_ of 0.8 was reached, followed by overnight incubation. The expressed protein was purified as previously described. The NMR sample of (**1**) contained 20 mM Tris, 20 mM Bistris, 0.5 mM ethylenediaminetetraacetic acid, and 3 mM sodium azide, at pH 6.0, plus 3.3 M GuHCl, all in 90 % H_2_O/10 % D_2_O. The protein concentration was approximately 0.5 mM. The uniformly ^15^N-labeled ubiquitin sample (1 mM, 90 % H_2_O/10 % D_2_O; pH 7.0) was purchased from Cortecnet (Giotto Biotech).

The sensitivity-enhanced HSQC experiments were recorded using samples of unlabelled (6.5 mM, PAF^D55N^) and ^15^N-labelled (1.7 mM, PAF^D19S^) mutants of *Penicillium* antifungal protein (PAF) in 95 % H_2_O/5 % D_2_O using 20 mM Na_3_PO_4_ pH 6.0 buffer, 40 mM NaCl, 0.04 % NaN_3_, as 275 μL of solution in Shigemi NMR tubes. The molecular mass of (**3**) was ~6.2 kDa and the average proton *T*_2_ relaxation time was ~55 ms at 25 °C (estimated from linewidth). The proteins were expressed and purified as previously described (Batta et al. 2009; Váradi et al. 2013).

The NMR experiments on (**1**) and (**2**) were carried out using an 11.7 T (500 MHz for ^1^H) Agilent/Varian VNMRS spectrometer equipped with a triple resonance HCN triple-axis gradient probe of maximum *xy* and *z* gradients 37.2 and 68.6 G cm^−1^, respectively. 960 or 1024 complex points were acquired (and zero-filled up to 16,384 complex points) at a spectral width of 5 kHz, and 32 scans were accumulated in order to achieve the very good signal-to-noise ratio needed to allow detailed comparison between different methods. Data acquisition in the pure shift experiments typically consisted of 4–8 chunks. The spectral width in the indirect dimension (^15^N) was 1500 Hz, and 128 increments (zero-filled up to 1024 complex points) were acquired with a 5 s relaxation delay for the L80C mutant of the N-terminal domain of phosphoglycerate (**1**). In the case of ubiquitin (**2**) the spectral width in the indirect dimension (^15^N) was 2000 Hz and 64 increments (zero-filled up to 256 complex points) were acquired with a 3 s relaxation delay. The mother experiments, needed for comparison, were run with identical experimental parameters; the small difference (50–80 ms per transient for BIRD, significantly less for BASHD) in acquisition time due to the pure shift sequence elements was compensated for by adjusting the relaxation delay to give the same overall experiment duration. Traditional time-shared homodecoupling was achieved by using 87 % of the dwell time for sampling the free induction decay, 10 % for radiofrequency irradiation, and 3 % for amplifier blanking and T/R switch delays. CTP selection gradient pulses in the pure shift elements (gBIRD and gBASHD; see Fig. 1c, d) were 0.5 ms long and of 16.6 and 13.6 G cm^−1^ amplitude. Gradient stabilization delays in the pure shift elements were 200 µs. Using too-short gradient stabilization delays (strongly dependent on the spectrometer used) can lead to a systematic frequency shift (of the order of 2 Hz) in the proton dimension, which can be easily corrected by internal referencing as normal, or cured by tedious optimization of the amplitudes of the CTP gradients using opposite polarities for g_1_ and g_2_. Amide band-selective refocusing was implemented with reBURP (Geen and Freeman 1991) 180° pulses of 2 kHz bandwidth and 2.44 ms duration for (**1**) and 2.2 kHz bandwidth and 2.22 ms duration for (**2**), with the transmitter offset set to the middle of the amide region. (If off-resonance pulses are used it is essential that these be phase coherent with the main sequence rather than simply being implemented with direct frequency jumps of the transmitter channel). In the case of the BIRD element, Broadband Inversion Pulses (BIP) (Smith et al. 2001) were implemented to minimize off-resonance effects, but these turned out to be negligible for proteins having reasonably small ^15^N spectral widths (see Supporting Fig. 4 for a comparison). Off-resonance effects are more important for small molecules, where efficient inversion is difficult to achieve using rectangular pulses. Time-shared homodecoupling was achieved using the standard implementation of VnmrJ 4.0 software. Parameters for sample (**1**) and (**2**) were the following: constant-adiabaticity CAWURST pulses with duration of 20 ms; decoupling bandwidth 1500 Hz; adiabaticity 1.2; duty cycle 13 % (87 % sampling, 10 % decoupling and 3 % switching time); t5 super-cycle (Tycko et al. 1985) phases: 0°, 150°, 60°, 150°, 0°. The shaped pulse was created with these parameters, but the pulse shaping program did not take into account the duty cycle. Therefore the power level of the homodecoupling was increased up to, where sufficient decoupling was achieved. The resonance offset for the decoupling shape was experimentally optimized at 4.5 ppm for (**1**) and 4.0 ppm for (**2**). Experiments were acquired regulating temperature to +25 °C, and the average proton *T*_2_ relaxation time of the samples (**1**) and (**2**) were ~60 and ~35 ms (estimated from linewidth).

The pure shift FIDs were automatically concatenated by the spectrometer hardware and all spectra (**1**) and (**2**) were processed identically for the comparison of different methods. Weighting functions were not applied in the direct dimension in order to best view the homonuclear couplings. The function exp(−*t* ^2^/gf1^2^) was used in the indirect dimension, where *t* is the time and gf1 was set to 0.085 and 0.032 s for sample (**1**) and (**2**) respectively. VnmrJ 4.0 software was used for data acquisition, processing and plotting. Water presaturation was not used in any of the experiments, and solvent subtraction was not used during data processing.

The NMR experiments on unlabelled and ^15^N-labelled mutants of PAF (**3**) were performed at 25 °C on a Bruker Avance II 500 spectrometer equipped with a TXI z-gradient probe (maximum gradient was 50.1 G cm^−1^), and the resulting data were processed with TopSpin 2.1 or 3.0. The spectra in Fig. 6 were recorded with the following parameters: the spectral widths in ^1^H (^15^N) dimension were 4.788 (21.0) ppm, number of data points in ^1^H dimension was 1024, number of *t*_1_ increments was 128, number of scans was 128, relaxation delay was 1.8 s. Prior to 2D Fourier transformation, the data were apodized by multiplying with 90° shifted sine-squared function along both dimension and zero-filled up to 2048 × 512 complex points in F2 × F1. Data acquisition in the pure shift ^1^H–^15^N HSQC-SE experiment was divided into 8 chunks, and the length of each chunk was 26.7 ms.

## Results and discussion

**Fig. 2:**
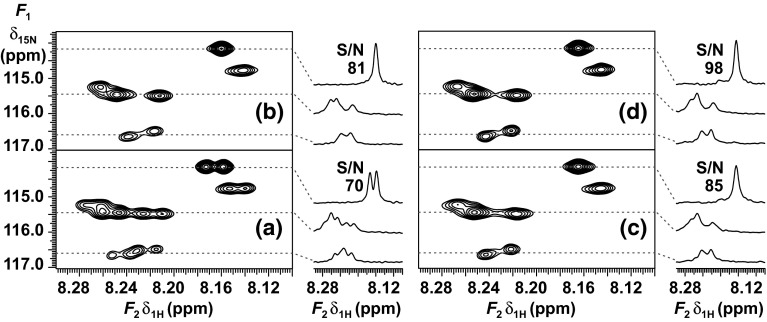
Partial ^1^H–^15^N-HSQC spectra of the L80C mutant of the N-terminal domain of phosphoglycerate kinase (**1**) in 3.6 M guanidine hydrochloride in 90 % H_2_O/10 % D_2_O, acquired using **a** HSQC, **b** HSQC with CAWURST time-shared homonuclear decoupling during acquisition, **c** pure shift gBIRD-HSQC, and **d** pure shift gBASHD-HSQC. Selected traces are shown at δ_15N_: 114.15, 115.34 and 116.55 ppm, respectively. Signal-to-noise (S/N) ratio is given for the top traces (δ_15N_ = 114.15 ppm), which are not distorted by overlap of resonances. The other two traces are shown to compare the resolution enhancement that was achieved by homodecoupling in **b**–**d**

Partial ^15^N HSQC spectra of (**1**) recorded with different acquisition schemes, including gBIRD, gBASHD and conventional time-shared homonuclear decoupling, are compared in Fig. 2. There are two well resolved and five partially overlapping doublets in this region of the HSQC spectrum. In the real-time pure shift spectra, all doublets have collapsed to singlets. Selected traces are shown next to each contour plot to illustrate performance. The less shielded resonances, around 8.24 ppm, show severe spectral overlap in the standard HSQC spectrum. There are two and three overlapping doublets at δ_15N_ = 116.55 ppm (bottom trace) and δ_15N_ = 115.34 ppm (middle trace) respectively. Separate integration of these cross-peaks is clearly impossible in the standard HSQC spectrum because of the overlapping multiplet structures. Neither automated nor manual peak-picking can determine all chemical shifts accurately in this region, particularly when short acquisition times (<100 ms) and line-broadening weighting functions mask the fine structure of the amide resonances. Pure shift NMR is a powerful tool in this example because each resonance appears as a single peak, making peak picking relatively trivial. All the five chemical shifts are resolved for peak-picking in the homodecoupled spectra, see bottom and middle cross-sections of Fig. 2b–d. There is relatively little difference between the pure shift spectra at first glance, but systematic comparison reveals that the resonances are slightly broader in the BIRD spectrum. This can be rationalized by the effect of *T*_2_ relaxation during the longer *J*-refocusing elements in BIRD. The implementation of traditional time-shared homodecoupling also improves resolution in this example, but it has some major drawbacks, discussed below, that hamper its routine application.

Besides enhancing the resolution, the real-time pure shift method also improves the sensitivity of the HSQC experiment, slightly but usefully. Signal-to-noise (S/N) ratios are given for the most shielded resonance at δ_15N_ = 114.15 ppm in each experiment in Fig. 2. The highest signal-to-noise (S/N) ratio was achieved by gBASHD and corresponded to c.a. 40 % enhancement relative to the standard HSQC spectrum (Fig. 2a). However, this sensitivity enhancement will vary from resonance to resonance, for the following reasons. (1) The natural linewidths are not small compared to homonuclear *J*-couplings in proteins, so the theoretical maximum sensitivity enhancement, that is a factor of +100 % for doublets and triplets, is rarely achievable. The enhancement factor is larger for resonances with larger homonuclear couplings or slower *T*_2_ relaxation, but smaller for resonances with unresolved couplings. (2) There is a sensitivity penalty for real-time acquisition that is dependent on the duration of the *J*-refocusing sequence element. This is the main reason why the sensitivity of the band-selective method is slightly higher than that of BIRD for proteins. (3) Sensitivity is clearly affected by the efficiency of homodecoupling, which varies between methods. BASHD and time-shared methods are less effective at the edges of the selected frequency band, while the BIRD element is longer which leads to more relaxation loss between the data acquisition periods.

In summary, both sensitivity and resolution will generally improve for resonances showing multiplet structure in the HSQC spectrum. Radiofrequency pulse imperfections and *T*_2_ relaxation during the *J*-refocusing double spin echo elements of the acquisition blocks cause some extra loss of magnetization in the real-time pure shift experiments as compared to the mother experiment (Paudel et al. 2013). This leads to resonances in the pure shift spectrum that, while narrower than the multiplet they replace, are slightly broader than an individual multiplet component. However, significant loss of sensitivity is only expected when the natural line width becomes larger than the scalar couplings, at which point pure shift methods are in any case inappropriate. The sensitivity gain from homodecoupling for a doublet vanishes around *J T*_2_ = 0.1, where the coupling becomes unresolved (Kupče et al. 2002). In the experiments described here, real-time pure shift NMR improved the sensitivity usefully (typically by 30 %) on (**1**). Slightly better results were achieved using BASHD than BIRD, because the *J*-refocusing block between data acquisition blocks is shorter in the former. Notably, the duration of the BIRD element is 1/^1^ *J*_NH_ (typically 11.1 ms) independently of the field strength, whereas the duration of the BASHD element spanning the amide proton region of proteins (2.4 ms here) scales inversely with field strength. Therefore, at higher magnetic fields the advantage of the BASHD method increases. Conventional homonuclear decoupling by time-shared band-selective irradiation (Fig. 2b) also results in improved resolution, but has some significant disadvantages making this method unable to meet with the needs of most biomolecular applications. First, the exact positions of the peaks differ from the true chemical shifts because of the Bloch–Siegert effect (Bloch and Siegert 1940). Second, the water resonance lies within the chemical shift region of C_α_H so the band-selective irradiation very significantly reduces the efficiency of water suppression. This problem will be discussed in detail later. Third, the dwell time between data points is shared between acquisition (87 % of the dwell time) and decoupling period, reducing the sensitivity advantage of decoupling by about 7 % (Kupče et al. 2002). The signal-to-noise ratio difference observed here between time-shared homodecoupling (Fig. 2b) and real time gBASHD (Fig. 2d) experiments was significantly larger, apparently because of the increased *t*_2_-noise caused by imperfect water suppression in the former case.

All three decoupled spectra (b) to (d) in Fig. 2 show better sensitivity than the parent HSQC spectrum (a). The selected traces were plotted under identical conditions to allow direct comparison of sensitivity and line-width. BASHD outperformed BIRD, and both showed slightly better sensitivity, and much better resolution, than standard HSQC.

Although real-time pure shift acquisition adds to the complexity of an HSQC pulse sequence, only two additional basic parameters need to be chosen. They are the characteristics of the *J*-refocusing element (the duration 1/^1^J_NH_ of the BIRD element, or the bandwidth and pulse shape of the BASHD selective refocusing pulse), and the duration 1/sw_ps_ of the basic data acquisition chunk. The former can easily be automated and does not require input from the user. The latter is a compromise between the requirements to minimise homonuclear *J*-evolution, and to minimise signal losses through relaxation during and pulse imperfections in the *J*-refocusing element. If too small an sw_ps_ is used (longer chunks), *J*-evolution will lead to imperfect decoupling and the appearance of sidebands at multiples of sw_ps_. If sw_ps_ is too large (shorter chunks), frequent application of *J*-refocusing will lead to excessive signal loss and hence line broadening.

**Fig. 3:**
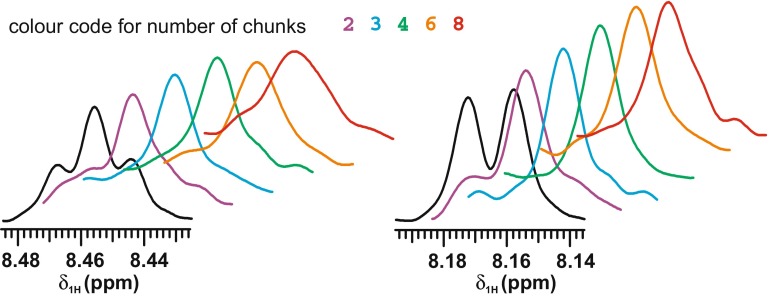
Selected *F*_2_ traces at δ_15N_ = 112.00 ppm (*left*) and δ_15N_ = 114.15 ppm (*right*) from the conventional HSQC spectrum (*black*) and from BIRD pure shift HSQC spectra (*colour coded*) of (**1**), showing the effect of increasing the number of data acquisition chunks: 2 *purple*; 3 *blue*; 4 *green*; 6 *orange* and 8 *red* (i.e. increasing sw2 from 9.7 to 39.1 Hz) for a total acquisition time of c.a. 200 ms

For the systems studied here, a total acquisition time about 200 ms per measurement is needed to make full use of the resolution improvement offered by pure shift methods. The choice of sw_ps_ (and hence of the number of chunks) determines the balance between spectral artefacts and line broadening, as illustrated in Fig. 3. Two sets of traces, at different nitrogen chemical shifts, through the *F*_2_ dimension of different HSQC spectra are shown. The traces plotted in black are from a conventional HSQC spectrum, with no homodecoupling; the remainder are from BIRD-decoupled spectra with increasing values of sw_ps_ and hence number of chunks needed to form the c.a. 200 ms long free induction decay. The purple traces, acquired with data acquisition chunks of 102.4 ms each (sw_ps_ = 9.7 Hz), clearly show the expected sidebands at ±sw_ps_, but by the green pair, corresponding to 4 chunks of 51.2 ms (sw_ps_ = 19.5 Hz) the sidebands are negligible.

Increasing the number of chunks (and sw_ps_) further simply results in a slight line broadening, as signal losses through relaxation and pulse imperfection increase. As noted earlier, a very small systematic shift in proton frequency, typically <2 Hz, can occur, which arises from finite gradient field recovery time; this effect was corrected by appropriate referencing. Alternatively, it is possible to cure the problem at source by alternating signs and magnitudes of successive gradient pulse pairs to cancel the average field error.

**Fig. 4:**
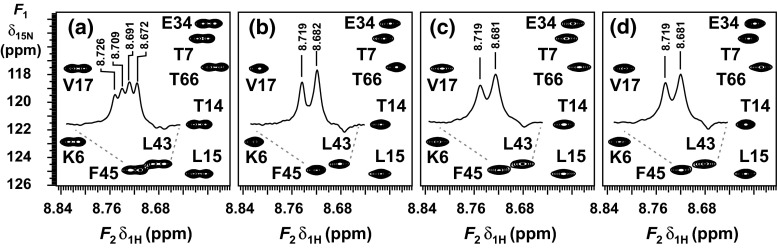
Partial ^1^H–^15^N-HSQC spectra of ^15^N-ubiquitin in 90 % H_2_O/10 % D_2_O (**2**) acquired by using **a** HSQC, **b** HSQC with CAWURST homonuclear decoupling during acquisition, **c** pure shift gBIRD-HSQC and **d** pure shift gBASHD-HSQC

The real-time pure shift experiments were also applied to ^15^N-ubiquitin (**2**), to facilitate comparison with standard methods, this time with 6 data chunks acquired in 200 ms (sw_ps_ = 30 Hz). The resolution enhancement achieved by different homodecoupling schemes is shown in Fig. 4. Both the time-shared homodecoupling and the pure shift methods reduce the spectral complexity by collapsing the multiplets to singlets. Here the narrowest lines were observed when using time-shared decoupling, but the water signal suppression was about an order of magnitude worse than for the real-time pure shift approaches, which would represent a significant obstacle to practical application.

In general, water suppression is particularly challenging for experiments that use homonuclear decoupling. Problems arise for real-time pure shift experiments because the double spin echo element inverts the magnetization of passive spins, which unfortunately includes that of water magnetization, from chunk to chunk. Because of radiation damping, water *z*-magnetization is partially transferred to the *xy* plane by the pure shift elements even with perfect radiofrequency pulses. A seemingly trivial solution would be to flip back water magnetization selectively during the double spin echo, but this would affect those C_α_H resonances close to or under the water resonance, leading to recoupling effects for the NH residues coupled to them. A better solution is to apply CTP selection gradient pulse pairs around the 180° rotations and extend the phase cycle by using 2 steps of EXORCYCLE on the pure shift elements. The use of CTP selection gradient pulses reduces the residual water signal by more than an order of magnitude, to a level comparable to that in a normal HSQC experiment. The minimum number of transients required is the same for pure shift and normal HSQC methods, but if more transients are used to improve sensitivity then implementation of the extended phase cycle can improve the results. Neither flip back nor CTP selection is possible for the time-shared decoupling, leaving water suppression as a major problem for this technique.

**Fig. 5:**
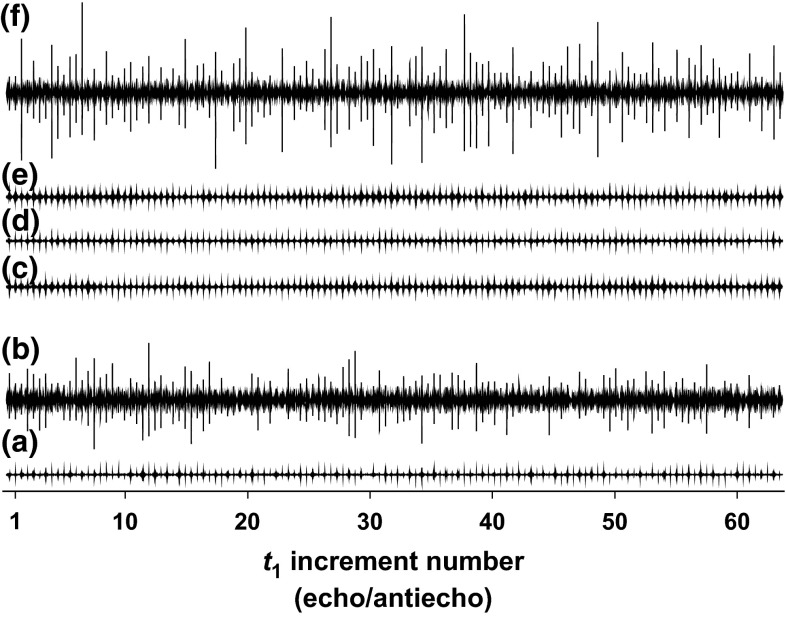
Residual water signal intensity in sample (**2**) as a function of *t*_1_ in **a** standard HSQC; **b** BASHD-HSQC (no PFG pulses in the pure shift element); **c** gBASHD-HSQC using only *z* gradient pulses and **d** and simultaneous *xy* gradients to enforce CTP selection in the pure shift elements; **e** gBIRD-HSQC using *xy* gradients; **f** HSQC with time-shared CAWURST homonuclear decoupling during acquisition. All spectra were plotted with *identical vertical scale*. Water presaturation was not used

Interestingly, the quality of pure shift spectra is influenced not only by the strength but also by the direction of the CTP selection gradient pulses. Figure 5 compares the water signal intensity in standard HSQC (Fig. 5a) and real-time BASHD experiments without (Fig. 5b) and with gradient pulses applied along the *z* axis (Fig. 5c) or in the transverse (*xy*) plane (Fig. 5d). Only the gradient pulses between the chunks were changed; other gradient pulses remained along the *z* axis. The strengths of the transverse gradient pulses were matched to the *z* gradient pulses by comparing the signal attenuation in DOSY (Nilsson et al. 2004) experiments. Transverse gradient pulses dephase the water magnetization more dependably, reducing unwanted signal in the 2D spectrum. If water suppression is crucial for a protein sample (e.g. because of low concentration or natural abundance) then triple gradient axis systems can give better results. The BIRD element also affects the water magnetization, and similar results were observed when adding CTP gradient pulses to the BIRD experiment (Fig. 5e).

As noted earlier, homodecoupling by time-shared irradiation with CAWURST pulses nicely enhances resolution, but water suppression is about an order of magnitude worse compared to real-time pure shift experiments, because the pulses are applied to the C_α_H resonances and hence also affect water magnetization. The poor water suppression and Bloch–Siegert shifts of resonances seen with time-shared irradiation have hampered the routine application of homodecoupling in protein NMR spectroscopy to date. The new real-time pure shift experiments can deliver good homodecoupling without such complications.

**Fig. 6:**
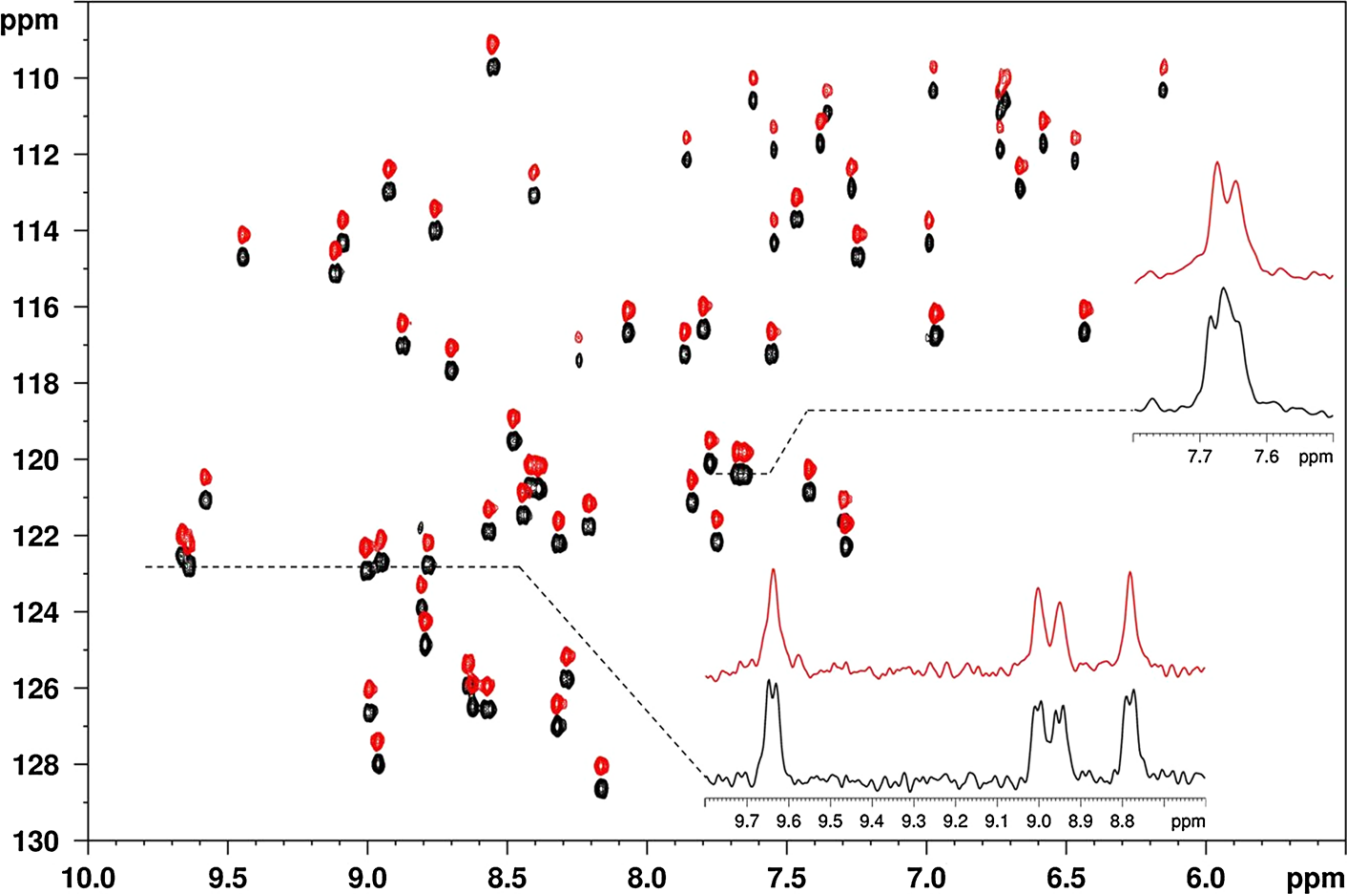
^1^H–^15^N HSQC-SE spectra of unlabelled mutant PAF^D55N^ (**3**) in 95 % H_2_O/5 % D_2_O without (*black*, *lower*) and with (*red*, *upper*) real-time pure shift gBIRD acquisition. The pure shift spectrum is shifted in the nitrogen dimension for easier comparison. The *top right inset* shows two overlapping peaks

To check the power and robustness of the gradient enhanced, BIRD-based broadband homodecoupling scheme of Fig. 1c, sensitivity-enhanced ^15^N HSQC spectra were recorded using the pulse sequence of Fig. S6 on samples of a non-labelled (6.5 mM) (Fig. 6) and ^15^N-labelled (1.7 mM) (Fig. S1 in Supplementary Material) mutant of *Penicillium* antifungal protein (PAF, 55 amino acid residue) in 95 % H_2_O/5 % D_2_O. The beneficial features of the pure shift HSQC sequence are illustrated in Fig. 6, which compares the sensitivity-enhanced HSQC spectra and representative *F*_2_ traces of the non-labelled PAF^D55N^ mutant recorded with the standard (black) and homodecoupled sequences (red). As expected, decoupling of proton–proton interactions yields a reduction in the linewidths observed, resulting in considerably improved resolution and so allowing unequivocal definition of peaks for automated data analysis.

The purging and coherence selection gradient pulse scheme employed in the sensitivity-enhanced pure shift HSQC sequence eliminates the strong water signal very efficiently, yielding clean and artefact-free spectra. The results shown clearly demonstrate that the pure shift methods proposed here are suitable for the study of small proteins at low (^15^N labelled) and standard concentrations.

## Conclusions

Practical homonuclear decoupling has been demonstrated for ^15^N HSQC spectra of proteins without any increase in experiment time. Such methods provide an increase in both resolution and sensitivity, so long as *T*_2_ is not limiting (in practice, for small proteins and for naturally disordered regions of larger proteins). Real-time pure shift experiments suffer from some sensitivity loss due to proton *T*_2_ relaxation, which also leads to slight broadening of the resonances. The collapse of multiplet structures into singlets compensates for the reduced sensitivity. BASHD performs slightly better than BIRD in this respect, because it uses a shorter *J*-refocusing pulse sequence element. The best resolution and sensitivity is however achieved with time-shared homodecoupling, provided that the poorer water suppression and the Bloch–Siegert effects can be tolerated. Both BASHD- and BIRD-based real-time pure shift methods deliver significantly better water suppression than homodecoupling by time-shared irradiation. In practice, the same level of water suppression can be achieved as in standard HSQC experiments if coherence transfer pathway selection with pulsed field gradients is used during data acquisition. The use of triaxial gradients is helpful, but not essential.

Pure shift methods are only useful where multiplet splittings are comparable to or greater than natural linewidths. If the natural linewidth is much greater than the homonuclear coupling then the disadvantages outweigh any advantages. In all other systems, real-time pure shift acquisition schemes should be able to replace normal acquisition, offering better resolution and similar or better sensitivity with no penalty in experiment time. The real-time pure shift acquisition schemes presented here should be compatible with any HSQC pulse sequence. Further exploration of their potential to improve standard 3D experiments (e.g. backbone assignment) is ongoing. A particularly important advantage of real-time pure shift methods over normal acquisition in ^15^N HSQC is that automatic peak-picking algorithms should no longer require that extra line broadening be used to obscure the fine structure of resonances. In a pure shift spectrum there is only one response from each chemically non-equivalent proton, making it ideal for chemical shift assignment.

## Electronic supplementary material

Below is the link to the electronic supplementary material.

Supplementary material 1 (DOC 5023 kb)
