# Supplementary material for: Real-time pure shift 15N HSQC of proteins: a real improvement in resolution and sensitivity
Source: J Biomol NMR. 2015 Mar 4;62(1):43–52. doi: 10.1007/s10858-015-9913-z (PMC4432093; doi:10.1007/s10858-015-9913-z)
Supplement: Supplementary file 1 — Supplementary material 1 (DOC 5023 kb) [file 10858_2015_9913_MOESM1_ESM.doc]

**Real-time pure shift ^15^N HSQC of proteins: a real improvement in resolution and sensitivity**

Peter Kiraly^1^, Ralph W. Adams^1^, Liladhar Paudel^1,2^, Mohammadali Foroozandeh^1^, Juan A. Aguilar^3^, István Timári^4^, Matthew J. Cliff^5^, Mathias Nilsson^1^, Péter Sándor^6^, Gyula Batta^7^, Jonathan P. Waltho^5^, Katalin E. Kövér^4^, Gareth A. Morris^1^,*

*^1^ P. Kiraly, L. Paudel, R.W. Adams, M. Foroozandeh, M. Nilsson and G.A. Morris, School of Chemistry, University of Manchester, Oxford Road, Manchester, M13 9PL, UK*

*^2^ Mitochondria and Metabolism Center, Department of Anesthesiology & Pain Medicine, 850 Republican St, University of Washington, Seattle, WA 98109*

*^3^ J. A. Aguilar, Department of Chemistry, Durham University, South Road, Durham, DH1 3LE, UK*

*^4^ I. Timári, K. E. Kövér, Department of Inorganic and Analytical Chemistry, University of Debrecen, Egyetem tér 1, H-4032 Debrecen, Hungary*

*^5^ M. J. Cliff, J. P. Waltho, Manchester Institute of Biotechnology, University of Manchester, 131 Princess Street, Manchester, M1 7DN, UK*

*^6^ Péter Sándor, Agilent Technologies R&D and Marketing GmbH & Co. KG, Hewlett-Packard Strasse 8. 76337 Waldbronn, Germany*

*^7^ G. Batta, Department of Organic Chemistry, University of Debrecen, Egyetem tér 1, H-4032 Debrecen, Hungary*

**Supplementary Material**


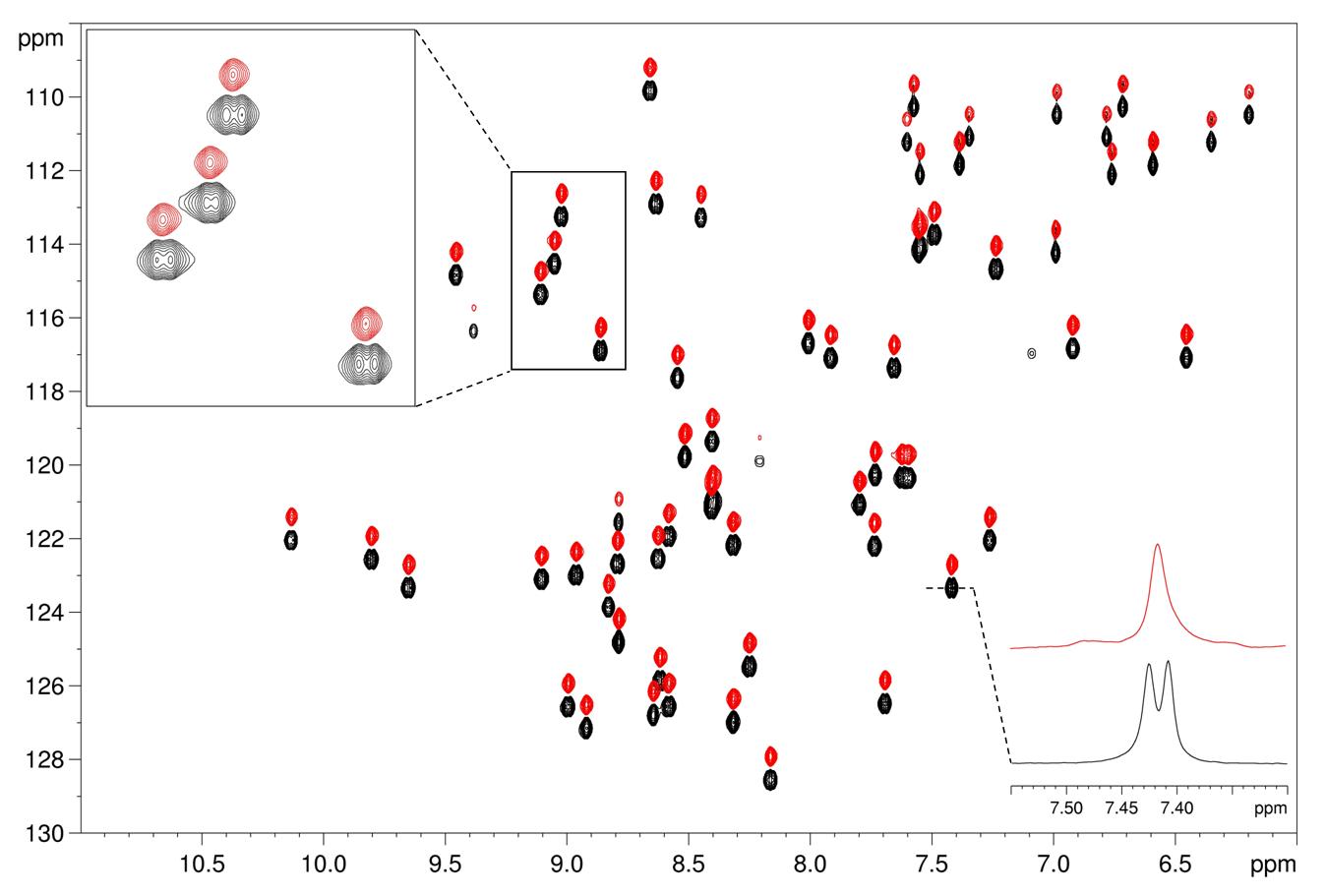


Supplementary Figure 1 ^1^H-^15^N HSQC-SE spectra of uniform ^15^N-labelled mutant PAF^D19S^ (3) in 95 % H_2_O / 5 % D_2_O without (black, lower) and with (red, upper) real-time pure shift gBIRD acquisition. The pure shift spectrum is shifted in the nitrogen dimension for easier comparison. The following parameters were used in these experiments: spectral widths in ^1^H (^15^N) dimension = 4.7880 (21.0) ppm, number of data points in ^1^H dimension = 2048, number of *t_1_* increments = 128, number of scans = 4, relaxation delay = 1.8 s. Data acquisition in the pure shift experiment was divided into 16 chunks and the length of each chunk was 26.7 ms. Traces in the inset are extracted at δ_15N_ = 123.3 ppm.


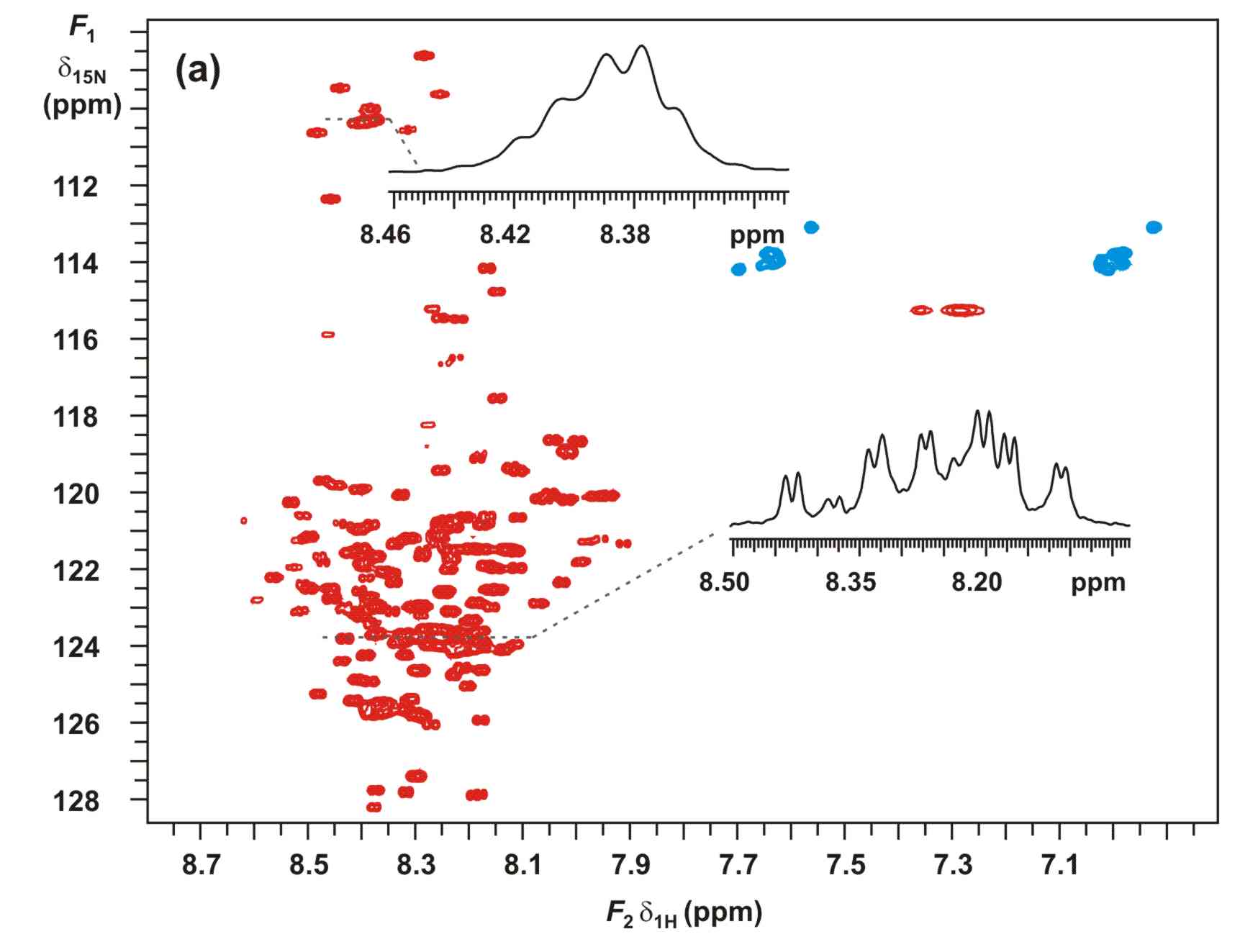

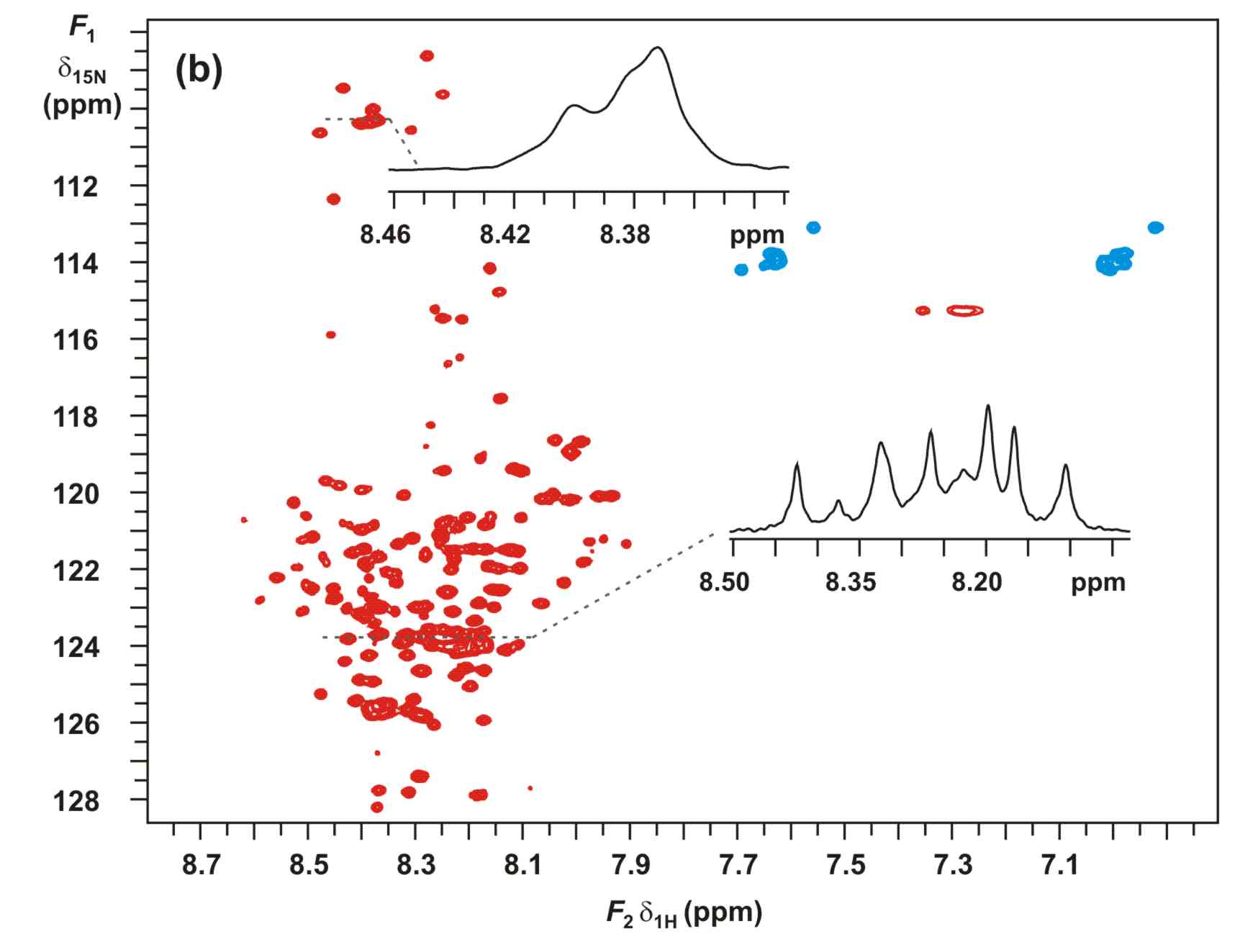

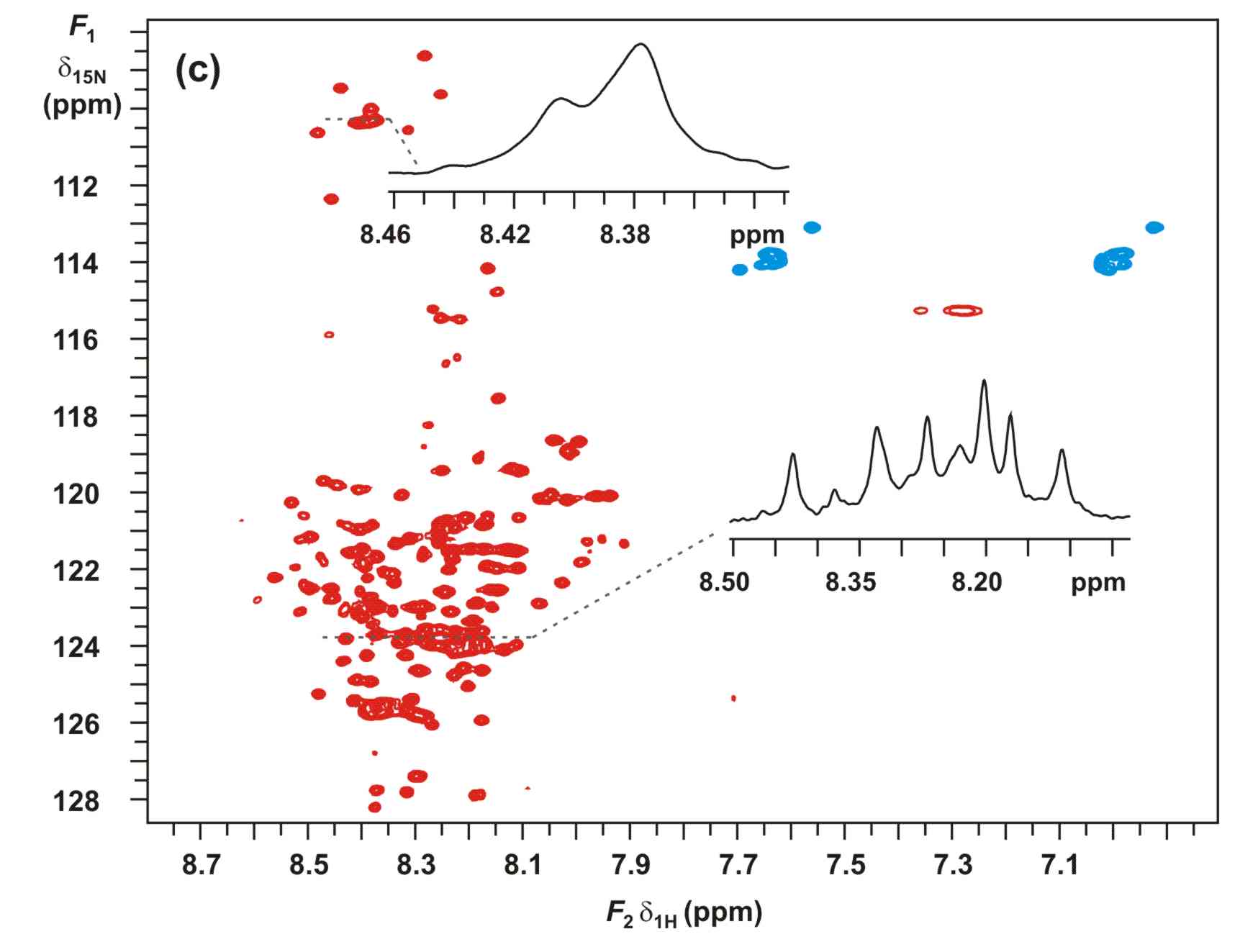

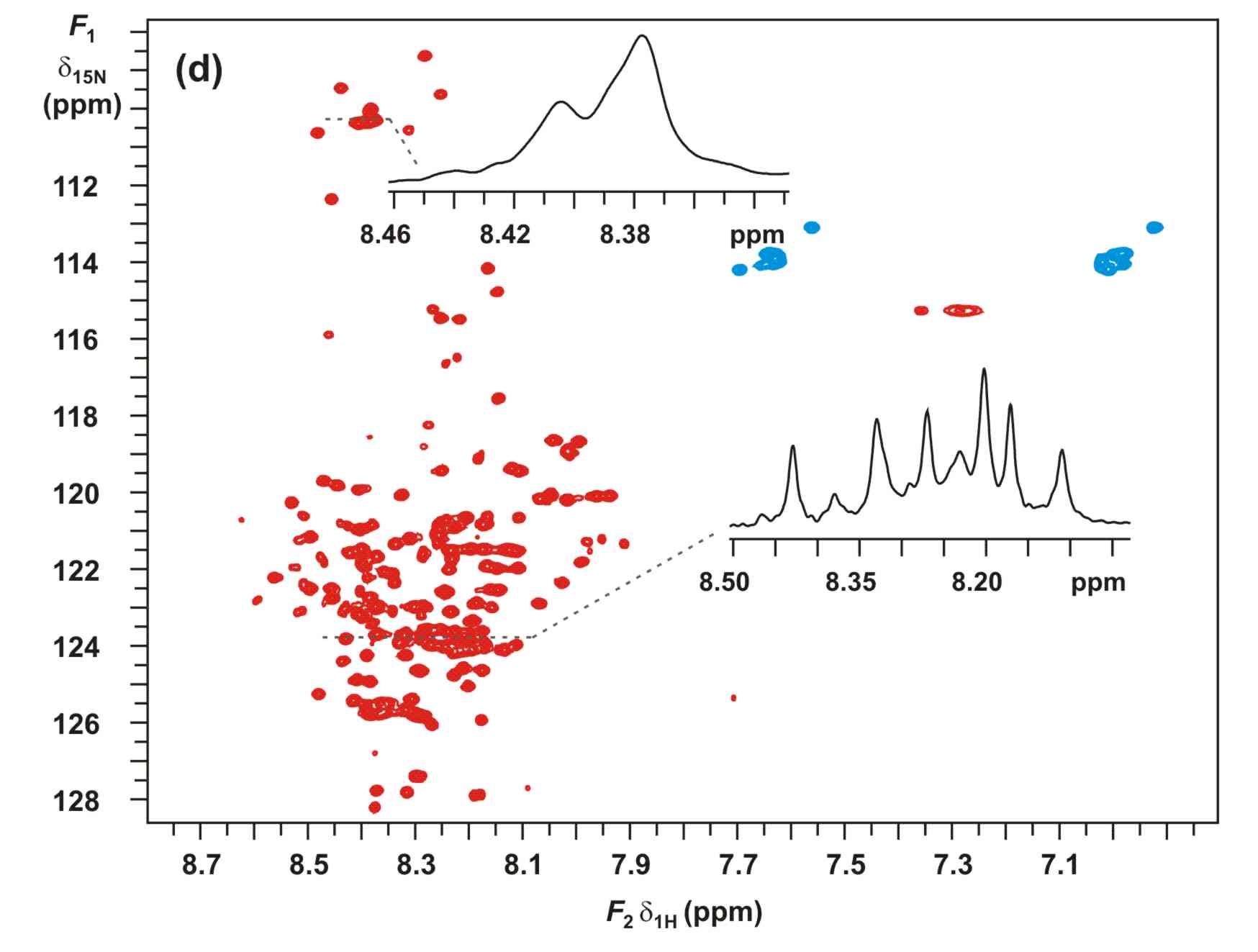


Supplementary Figure 2 ^1^H-^15^N-HSQC spectra of L80C mutant of the N-terminal domain of phosphoglycerate kinase (1) in 3.6 M guanidine hydrochloride in 90 % H_2_O / 10 % D_2_O acquired by using gHSQC (top left); CAWURST homonuclear decoupling during acquisition of gHSQC (top right); pure shift gBIRD-HSQC (bottom left); pure shift gBASHD-HSQC (bottom right). Insets are shown at δ_15N_ 110.29 and 123.87 ppm, respectively.


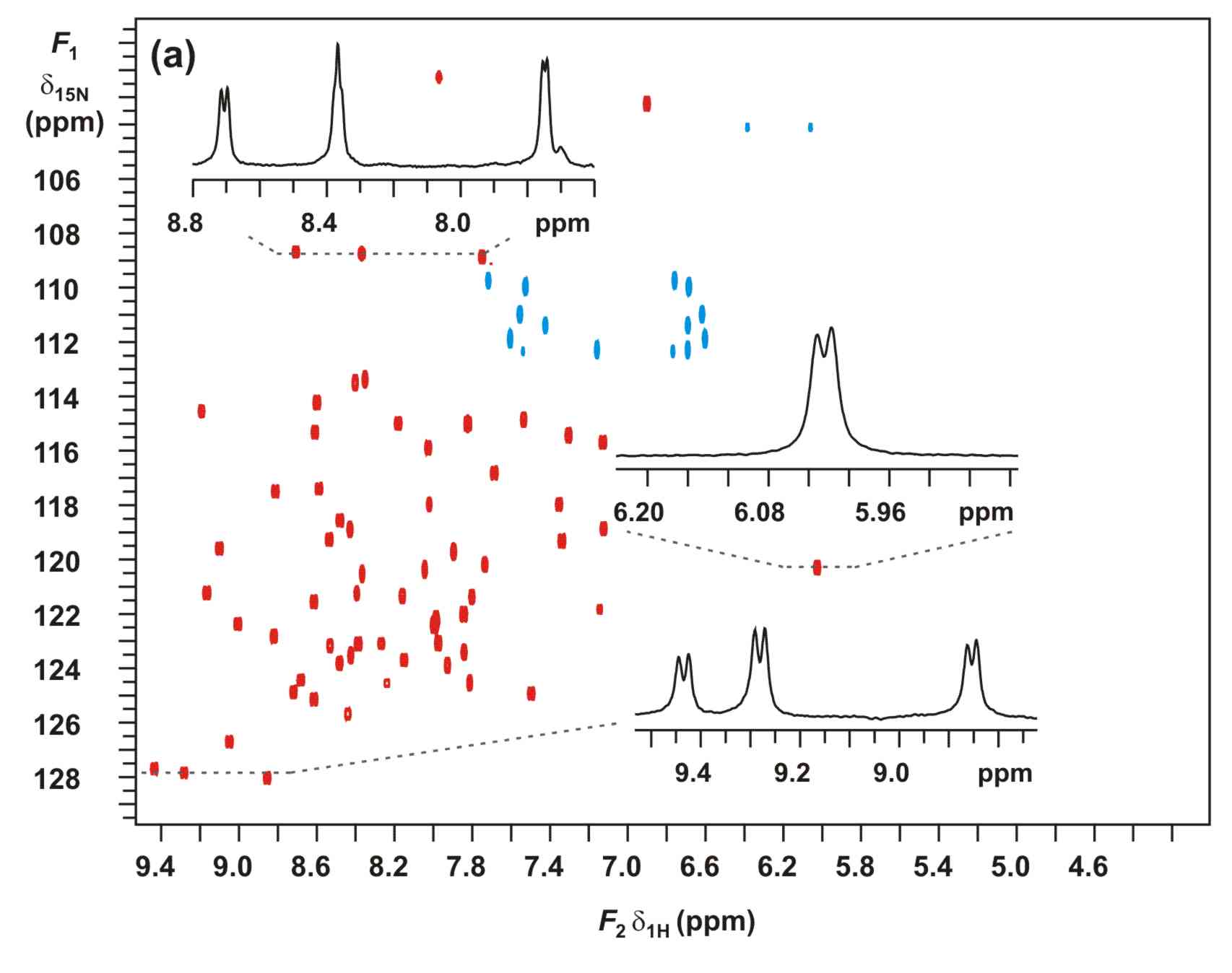

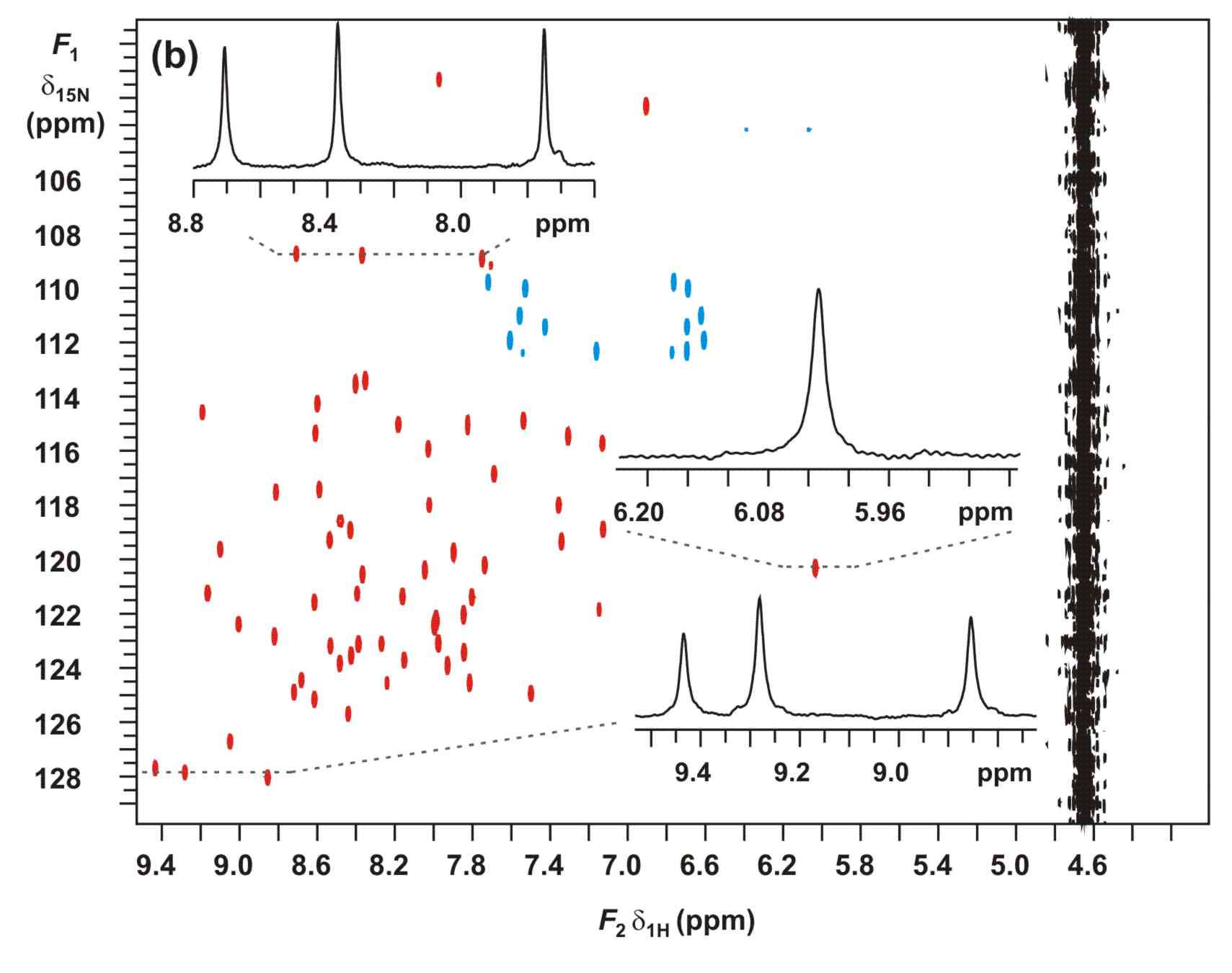

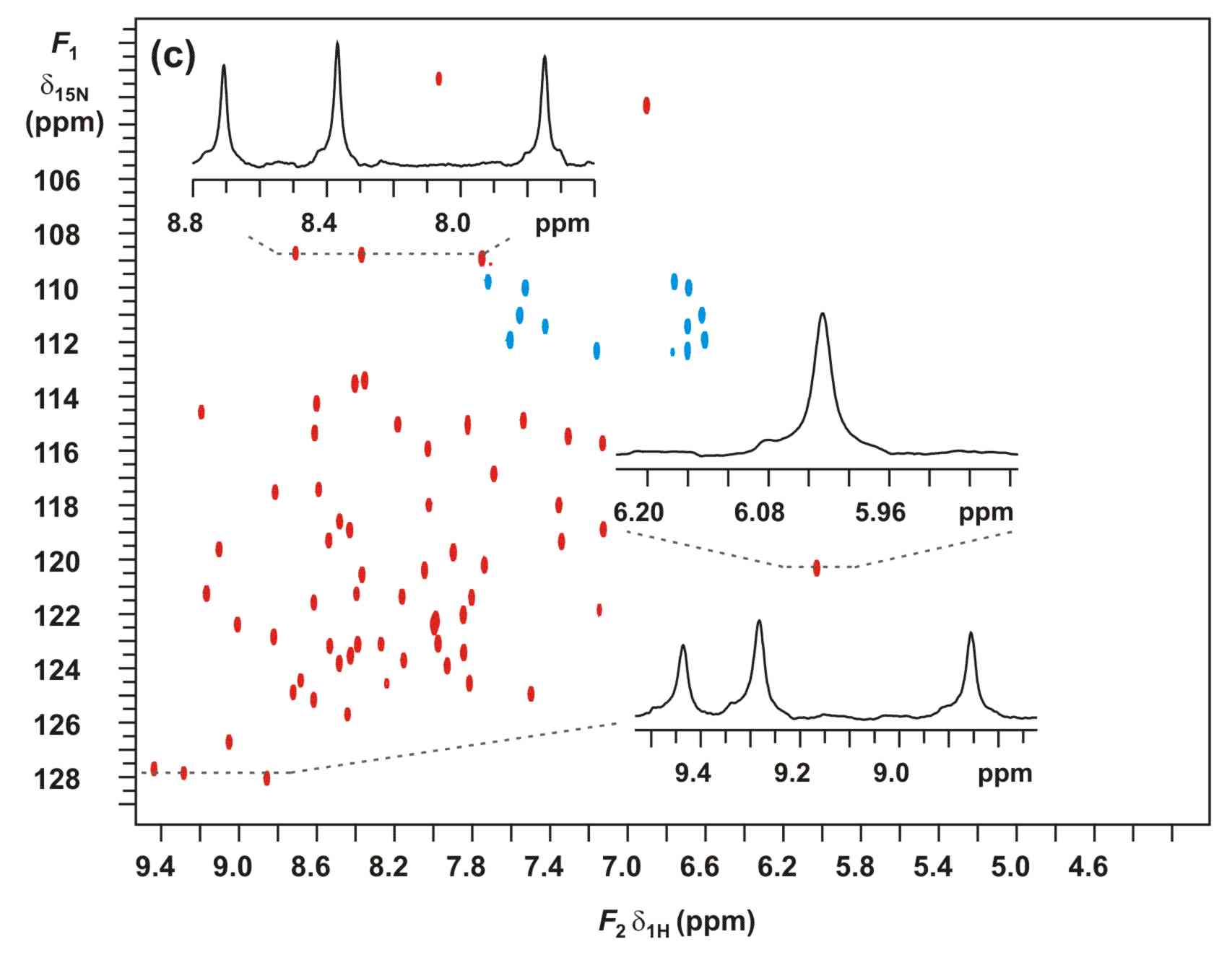

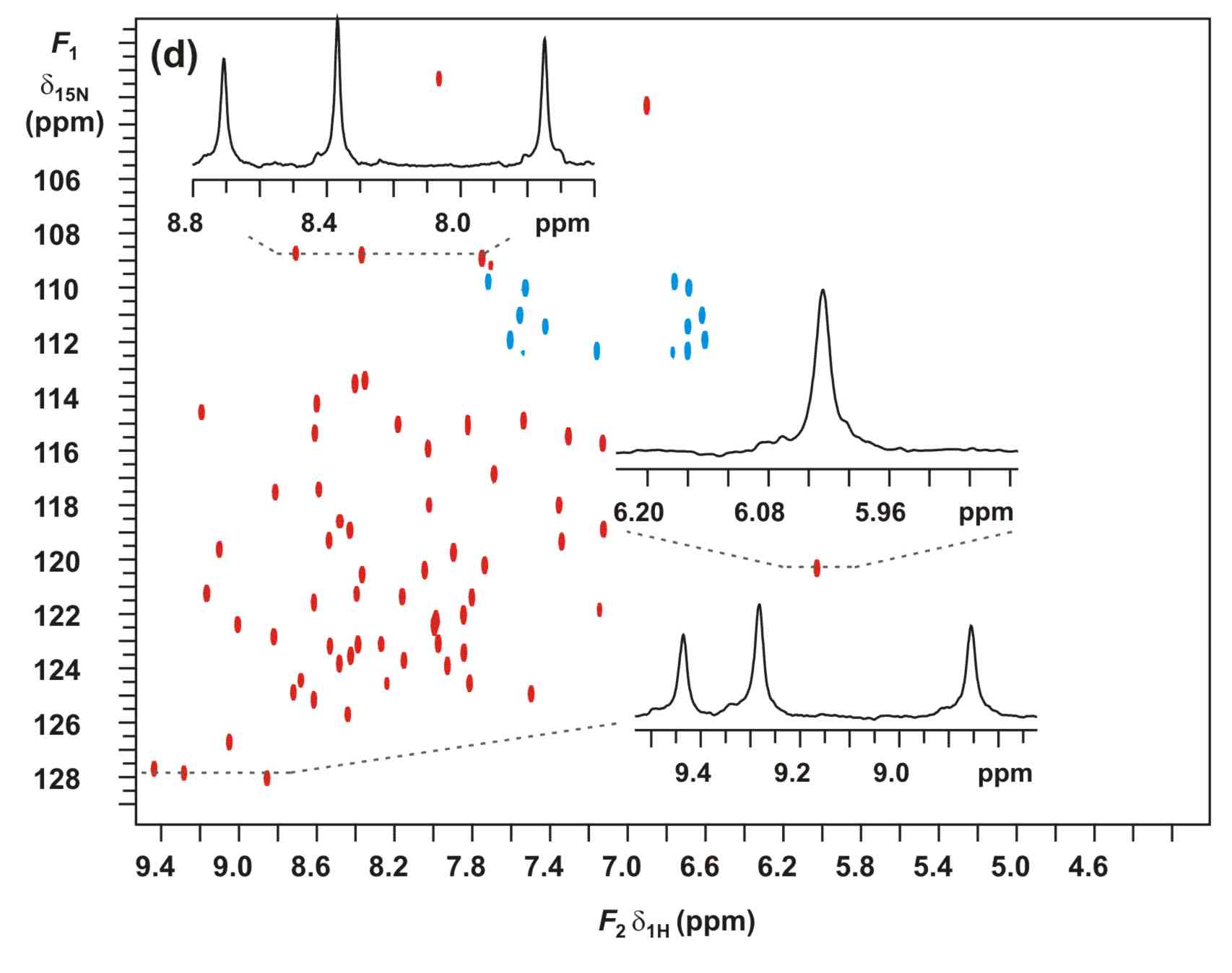

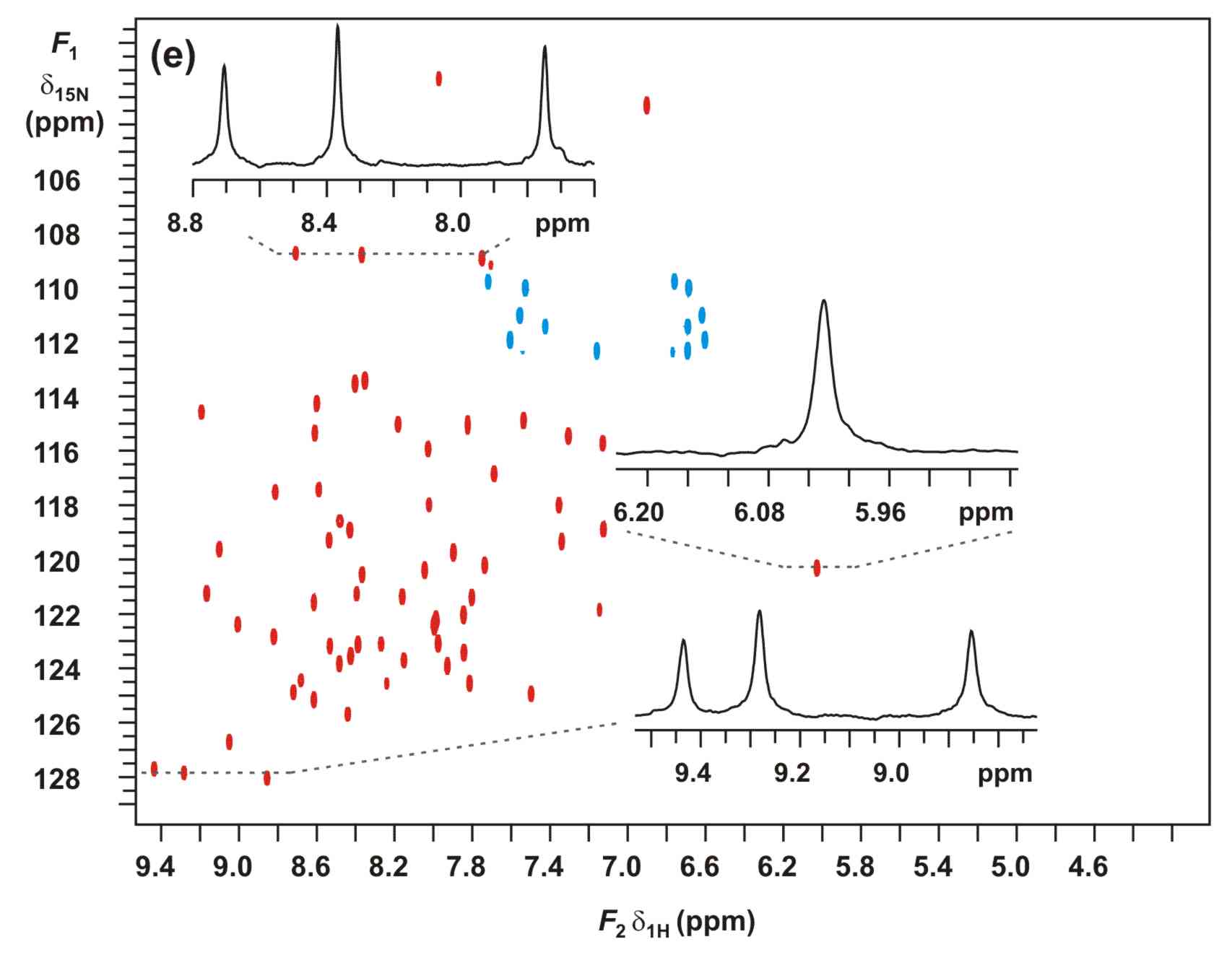

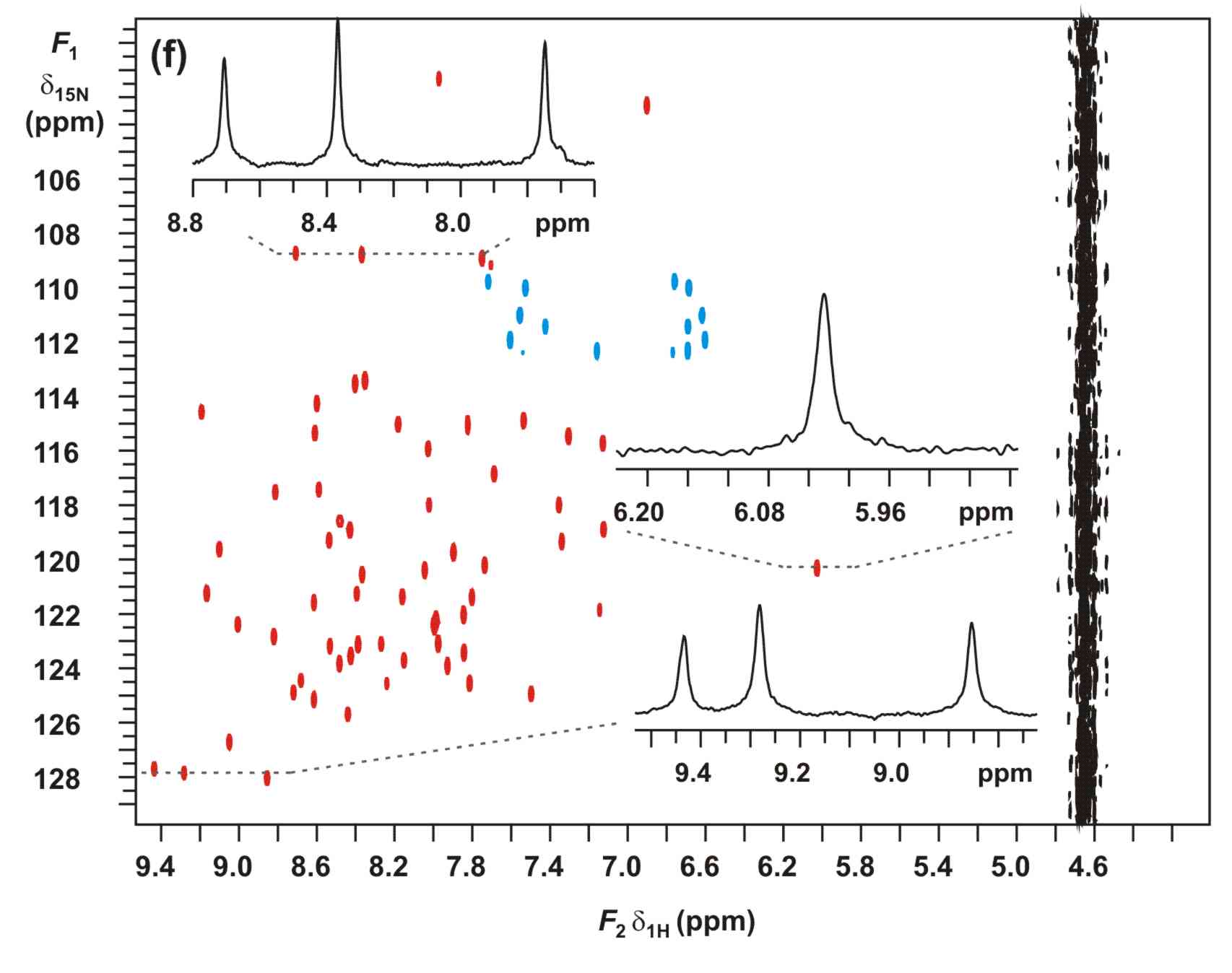


Supplementary Figure 3 ^1^H-^15^N-HSQC spectra of ^15^N-ubiquitin in 90% H_2_O / 10% D_2_O (2) acquired by using (a) HSQC (b) HSQC with CAWURST homonuclear decoupling during acquisition (c) pure-shift gBIRD-HSQC and (d-f) pure-shift gBASHD-HSQC: (d) with simultaneous *xy* gradients (e) with *z* gradients and (f) without gradients to enforce CTP selection in the pure shift elements. Insets are shown at δ_15N_ 108.84, 120.30 and 127.86 ppm, respectively.

**Table S1: Data for the middle traces shown in Figure S3 (δ_15N_=120.30ppm)**

|  | HSQC^1^ | HDEC^2^ | gBIRD^3^ | gBASHD*xy*^4^ | gBASHD*z*^5^ | BASHD^6^ |
| --- | --- | --- | --- | --- | --- | --- |
| signal-to-noise or  signal-to-artefact ratio | 224 | 52^§^ | 217 | 266 | 220 | 70^§^ |
| relative peak height | 100 | 131 | 109 | 126 | 118 | 122 |

^§^ noise region is strongly affected by the strong water signal

^1^ standard HSQC (see Figures S3a and S5a)

^2^ HSQC with CAWURST time-shared homonuclear decoupling during acquisition (see Figure S3b)

^3^ real-time pure shift HSQC with BIRD using CTP gradients (see Figures S3c and S5d)

^4^ real-time pure shift HSQC with band-selective homodecoupling using simultaneous *xy* gradients to enforce CTP selection (see Figures S3d and S5e)

^5^ real-time pure shift HSQC with band-selective homodecoupling using *z* gradients to enforce CTP selection (see Figures S3e and S5e)

^6^ real-time pure shift HSQC with band-selective homodecoupling (see Figures S3f and S5c)


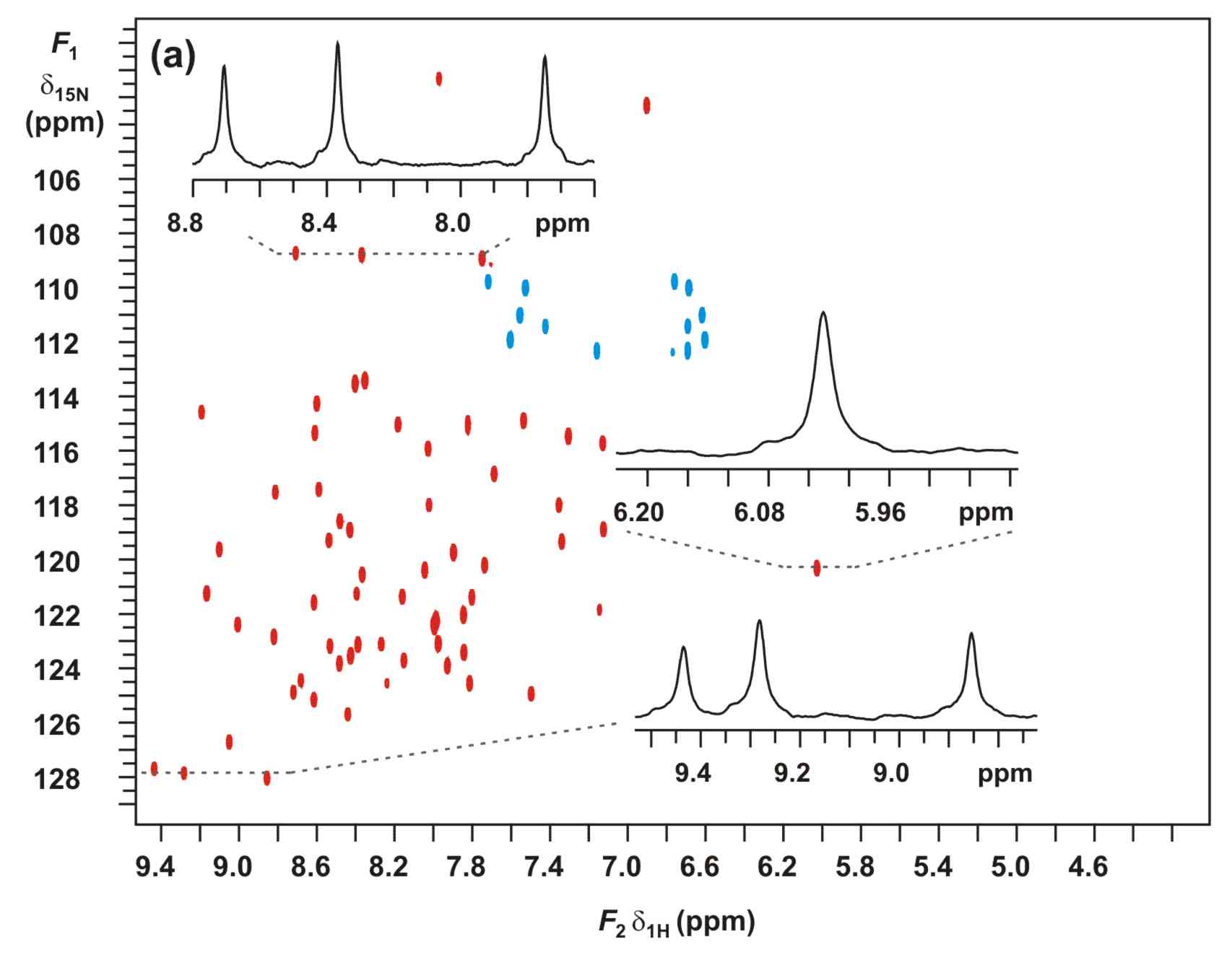

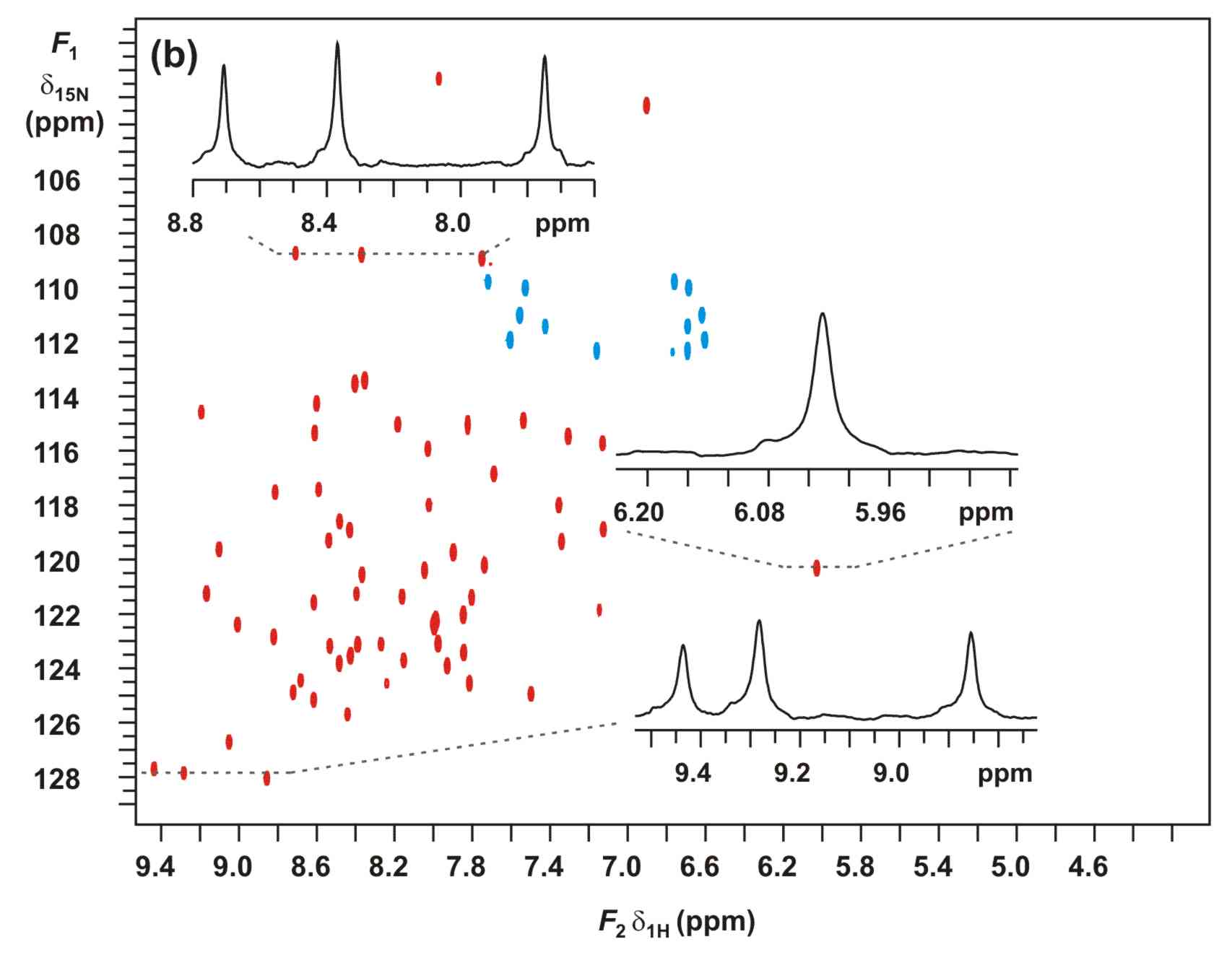


Supplementary Figure 4 ^1^H-^15^N-HSQC spectra of ^15^N-ubiquitin in 90% H_2_O / 10% D_2_O (2) acquired by using pure shift gBIRD-HSQC with hard (left) and BIP (right) 180° nitrogen pulses. Insets are shown at δ_15N_ 108.84, 120.30 and 127.86 ppm, respectively.

**Pulse sequence diagrams for real-time pure shift ^15^N HSQC**


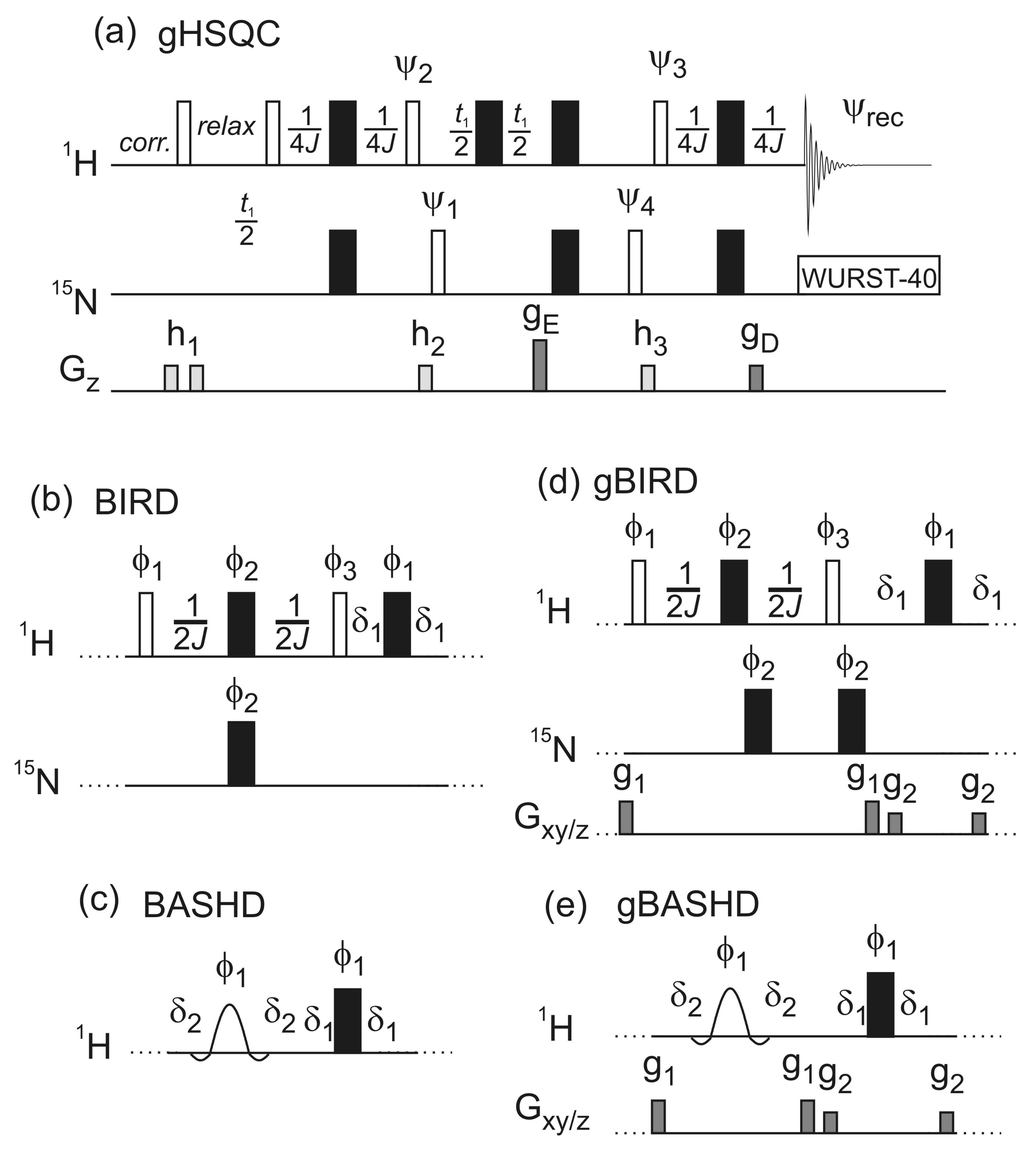


Supplementary Figure 5 Pulse sequence diagram of (a) gradient-selected and multiplicity-edited HSQC (denoted as gHSQC); (b) BIRD; (c) BASHD; (d) gBIRD and (e) gBASHD. Narrow and wide filled bars correspond to 90° and 180° pulses, respectively, with phase *x* unless indicated otherwise. The CTP gradients in the pure shift elements were applied simultaneously on the *x* and *y* channels when triaxial gradients were available. Only the acquisition scheme was changed for the pure shift pulse sequences. An extended phase cycle was implemented to provide further improvements when time averaging is used to improve sensitivity. gHSQC part: Ψ_1_={x,-x}; Ψ_2_={y,y,-y,-y}; Ψ_3_={[x]_4_,[-x]_4_}; Ψ_4_={[x]_8_,[-x]_8_}; Ψrec={y,-y,-y,y,[-y,y,y,-y]_2_,y,-y,-y,y}); pure-shift part Ψ_1_={x,x,y,y}; Ψ_2_={y,y,-x,-x}; Ψ_3_={-x,-x,-y,-y}; Ψrec={[y,-y]_2_,[-y,y]_4_,[y,-y]_2_,[-y,y]_2_,[y,-y]_4_,[-y,y]_2_}).


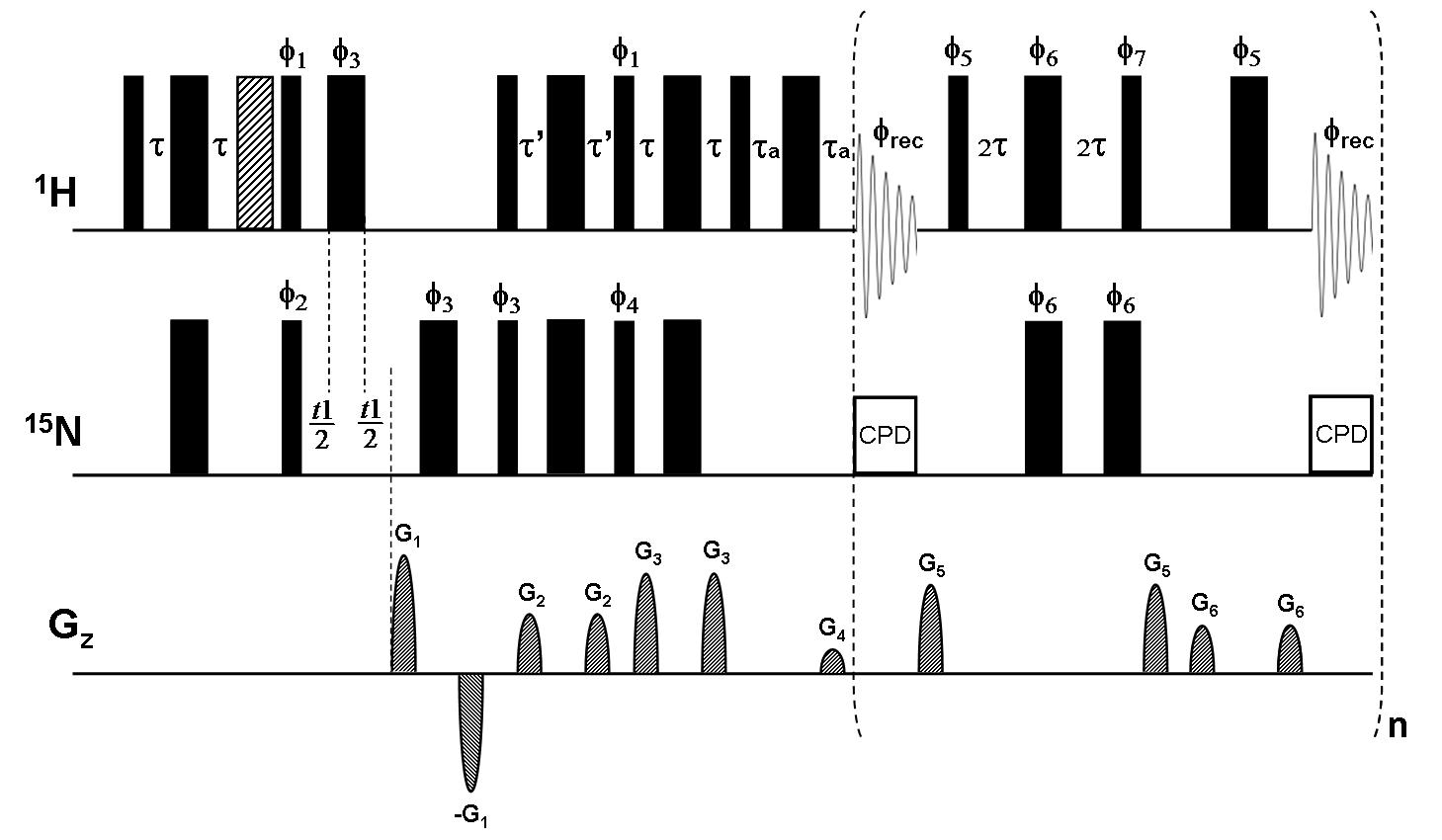


Supplementary Figure 6 Pulse sequence diagram for the real-time pure shift ^1^H-^15^N sensitivity enhanced HSQC experiment. Narrow and wide filled bars correspond to 90° and 180° pulses, respectively, with phase *x* unless indicated otherwise. Trim pulse during the initial INEPT step is shown as striped wide bar. Phases are *ϕ*_1_ = *y*; *ϕ*_2_ = *x, -x*; *ϕ*_3_ = *x, x, x, x, -x, -x, -x, -x*; *ϕ*_4_ = *y, y, y, y, -y, -y, -y, -y*; *ϕ*_5_ = *x, x, y, y*; *ϕ*_6_ = *y, y, -x, -x*; *ϕ*_7_ = *-x, -x, -y, -y*; and *ϕ*_rec_ = *x, -x, x, -x, -x, x, -x, x.* Delays are set as follows: τ = 1/(4* ^1^*J*_NH_), τ’ = 1/(4* ^1^*J*_NH_) for NH or 1/(8* ^1^*J*_NH_) for all multiplicities, τ_a_ = p16 + d16 + 8 μs. Coherence order selection and echo-antiecho phase sensitive detection in the ^15^N-dimension are achieved with gradient pulses G_1_, -G_1_ and G_4_ in the ratio 80 : -80 : 16.2. Purging gradient pulses G_2_ and G_3_ are set to 42 %, 67 % of maximum gradient strength (50.1 G/cm), typically with 1 ms duration (p16) followed by a recovery delay d16 = 100 μs. CTP selection gradient pulses: G_5_ = 61 % and G_6_ = 31 % are used around the BIRD block and the hard 180° proton pulse between data chunks with 0.5 ms duration followed by a recovery delay (100 μs). CPD decoupling is turned on during acquisition.


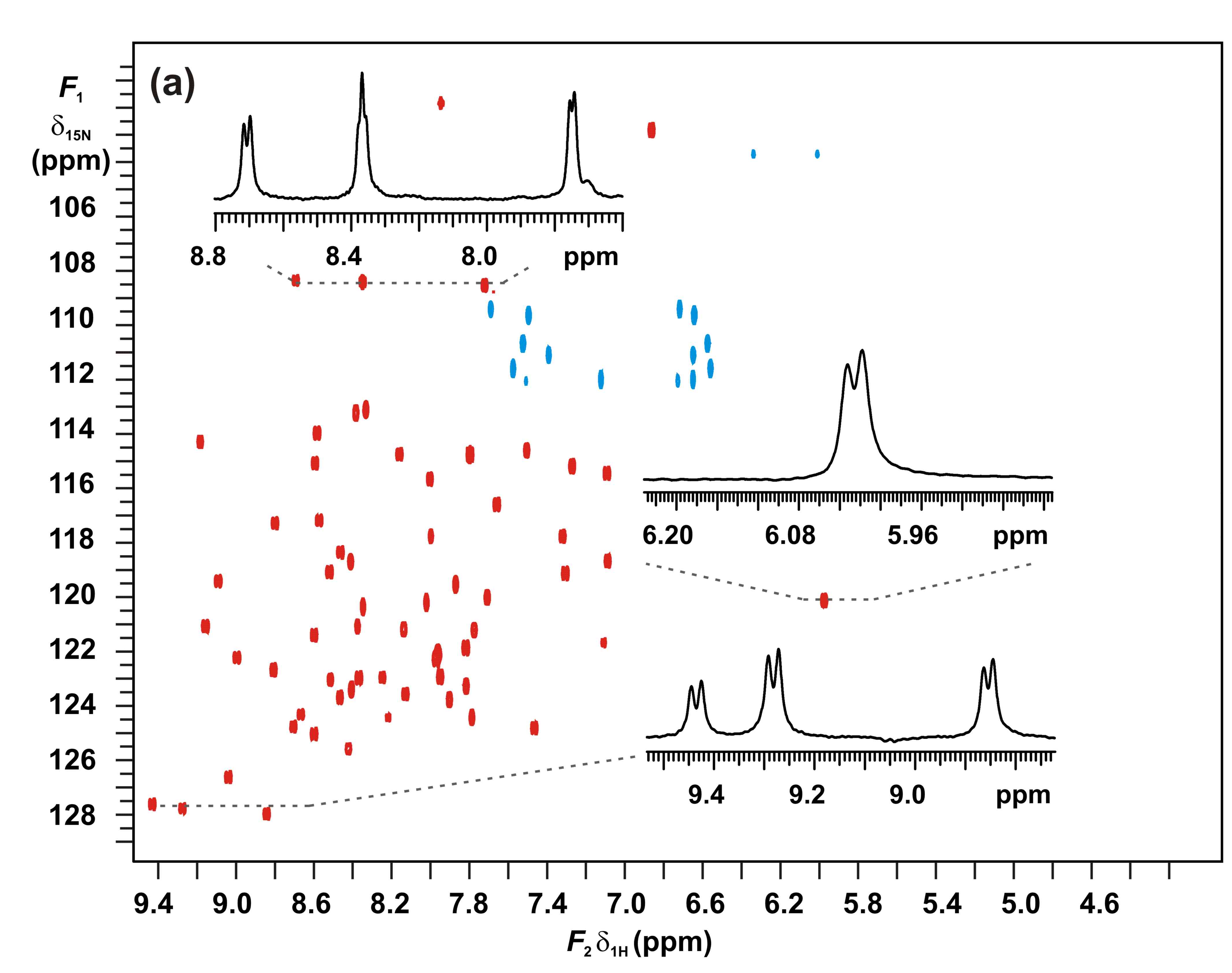

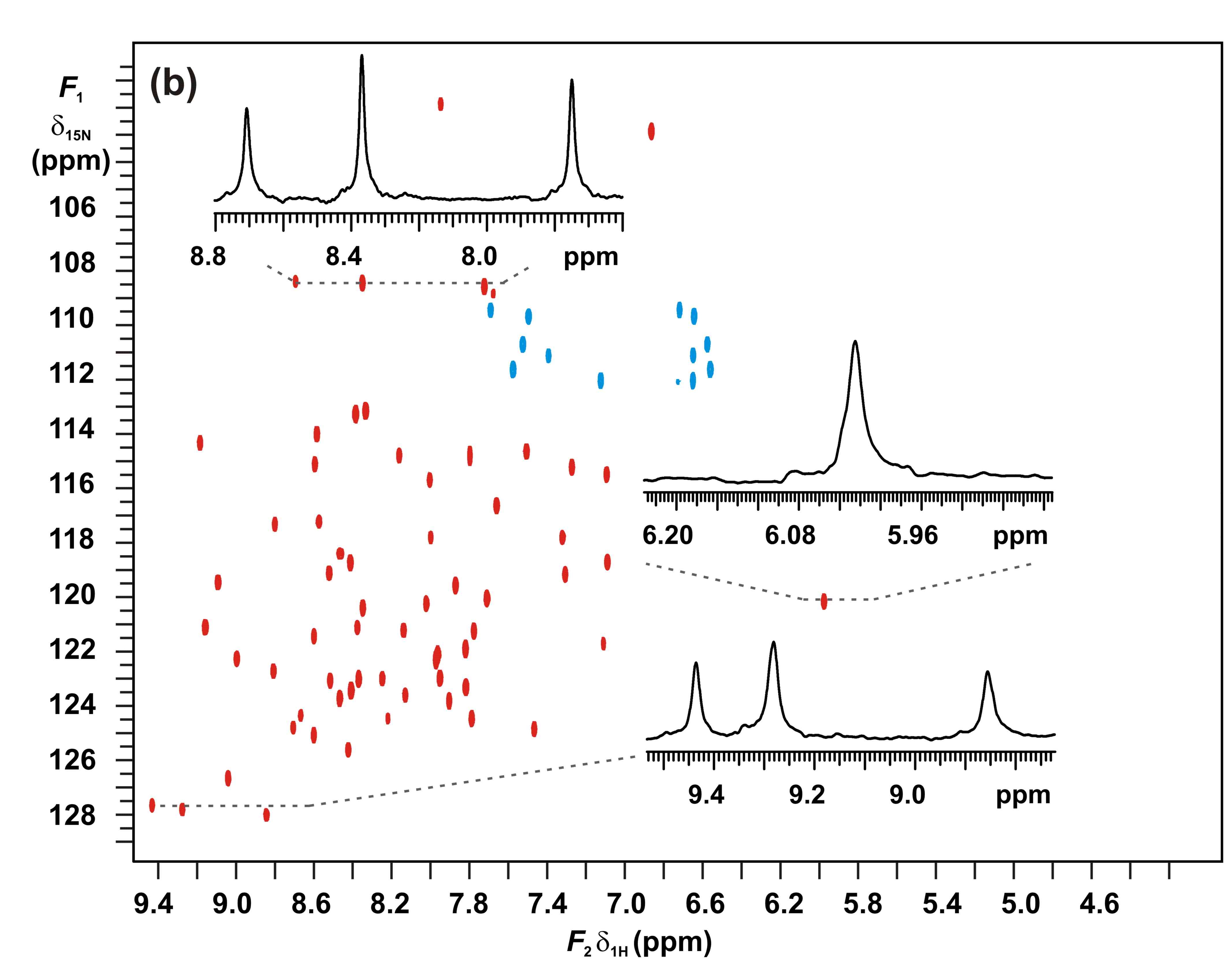

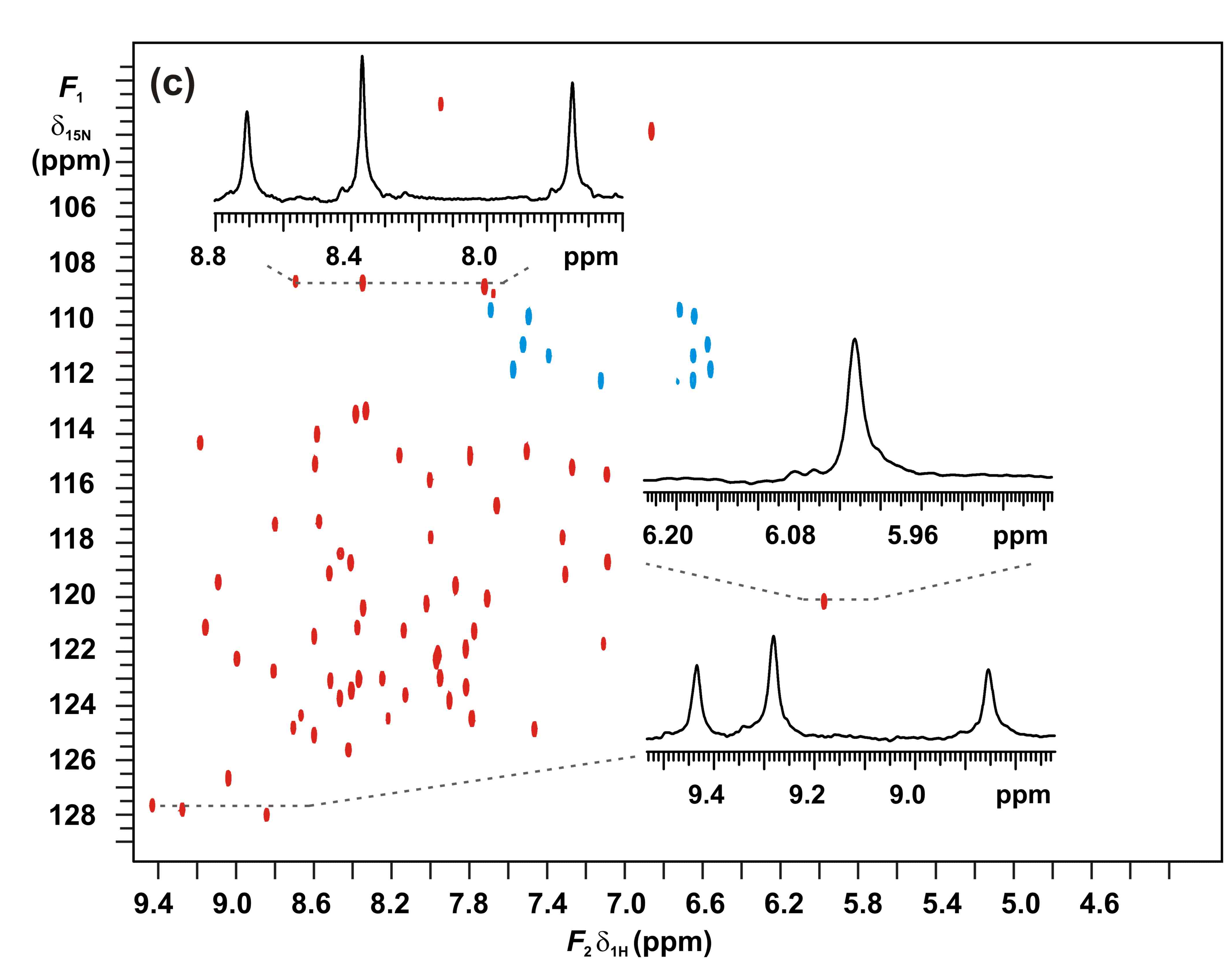

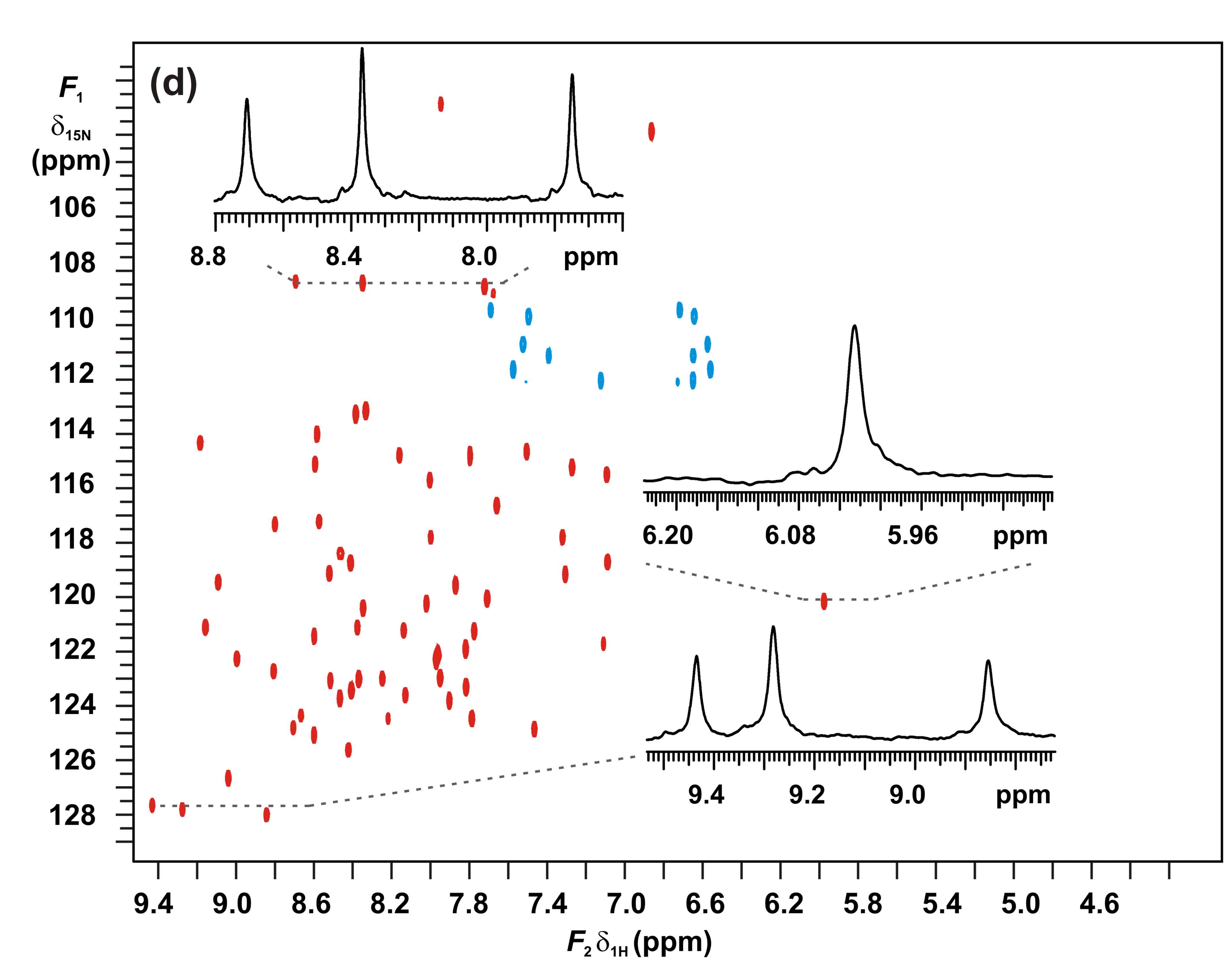


Supplementary Figure 7 ^1^H-^15^N-HSQC spectra of ^15^N-ubiquitin in 90% H_2_O / 10% D_2_O (2) acquired by (a) HSQC and real-time pure shift gBASHD-HSQC with (b) and without (c) chunk-to-chunk phase cycling of the J-refocusing element as suggested in (Ying 2014). These experiments were recorded by using the same 32 scans as for those shown on Supplementary Figure 3 and 4, but here only the first two steps of the phase cycling was used. In the case of (d) four steps were used from our phase cycling, which is more important than the MLEV type chunk-to-chunk phase cycling in our case. However, real-time pure shift experiments can be performed with the same minimal phase cycling as the normal HSQC if sensitivity limitation does not require more scans. See below a comparison for a selected resonance (I36). Insets are shown at δ_15N_ 108.84, 120.30 and 127.86 ppm, respectively.


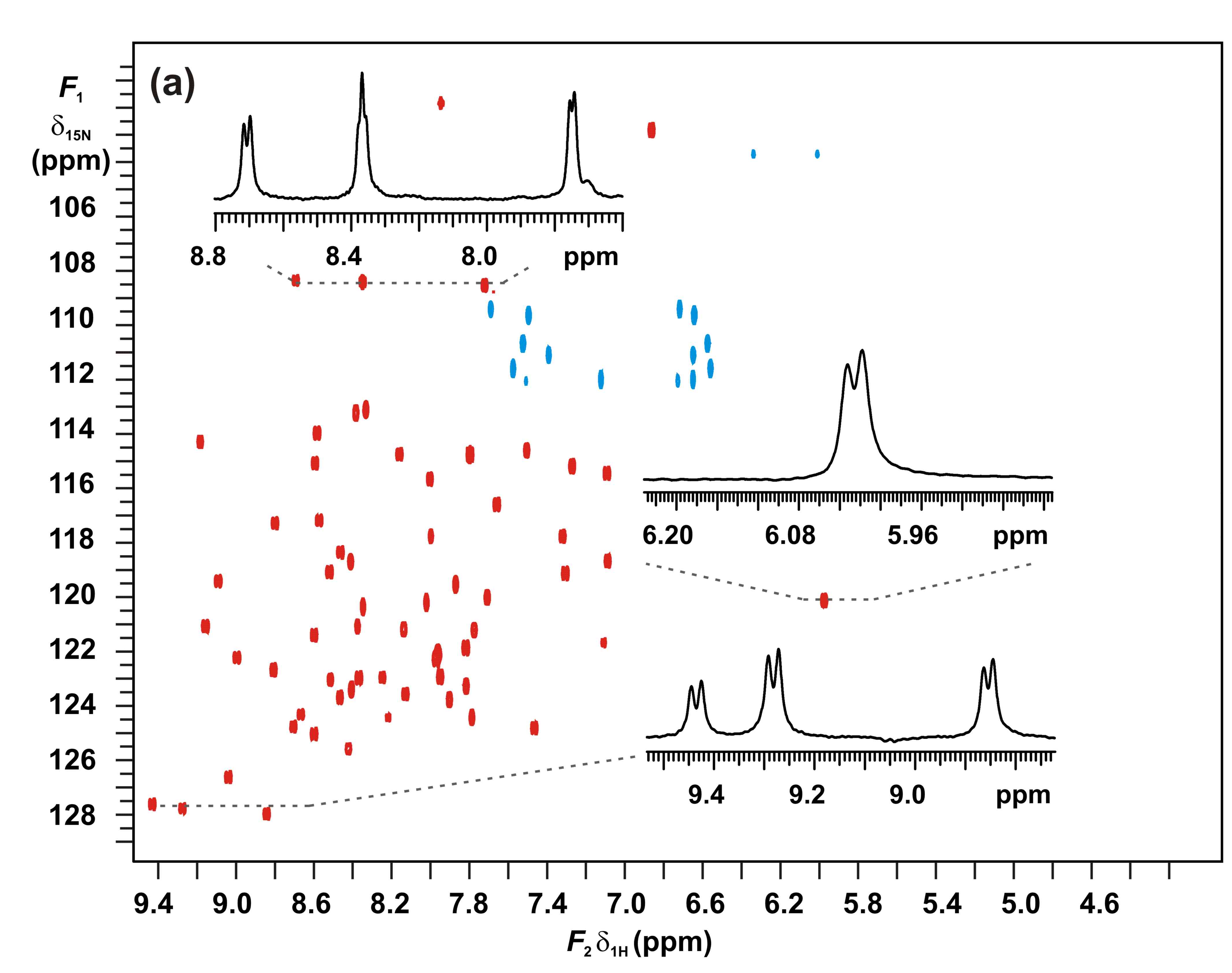

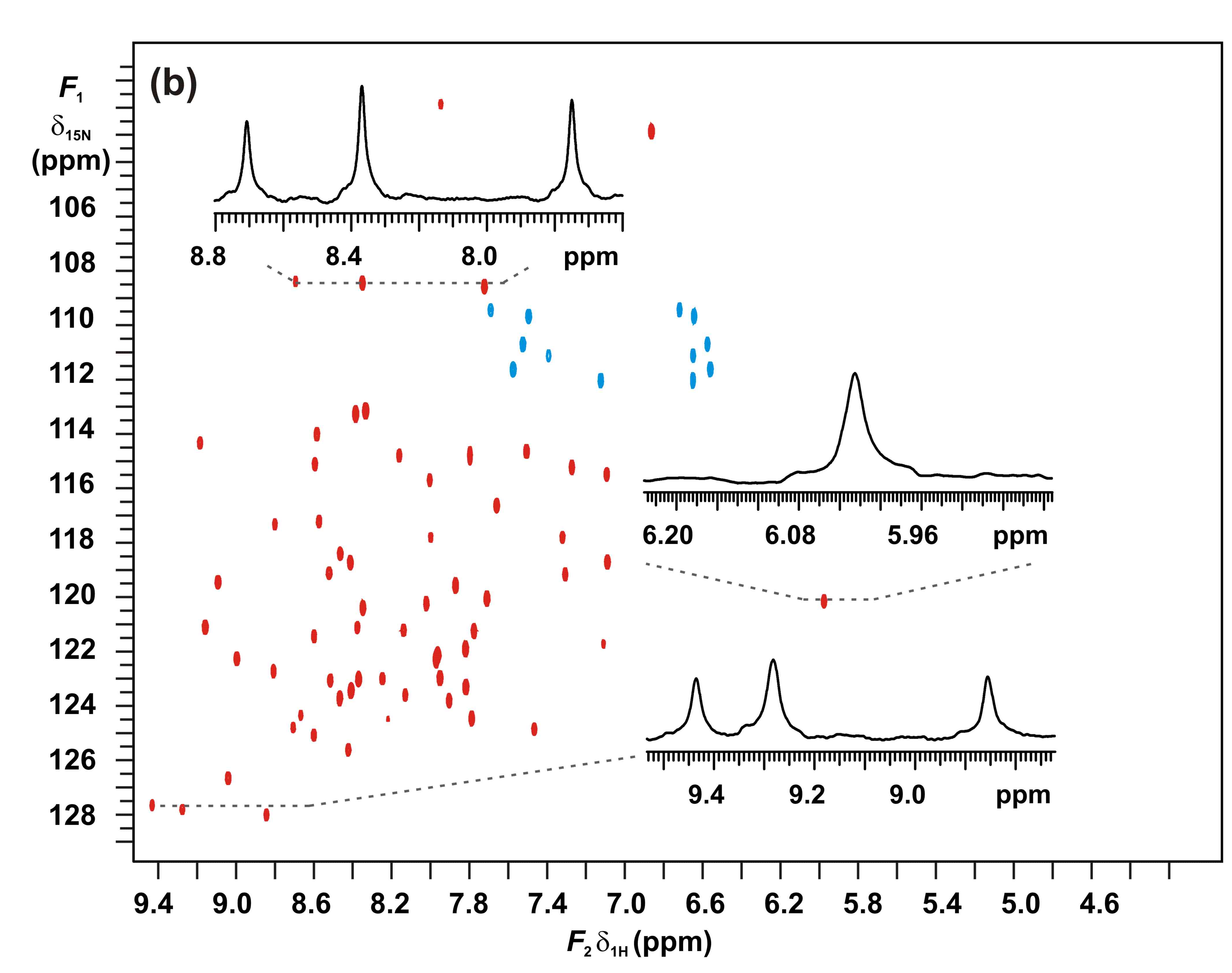

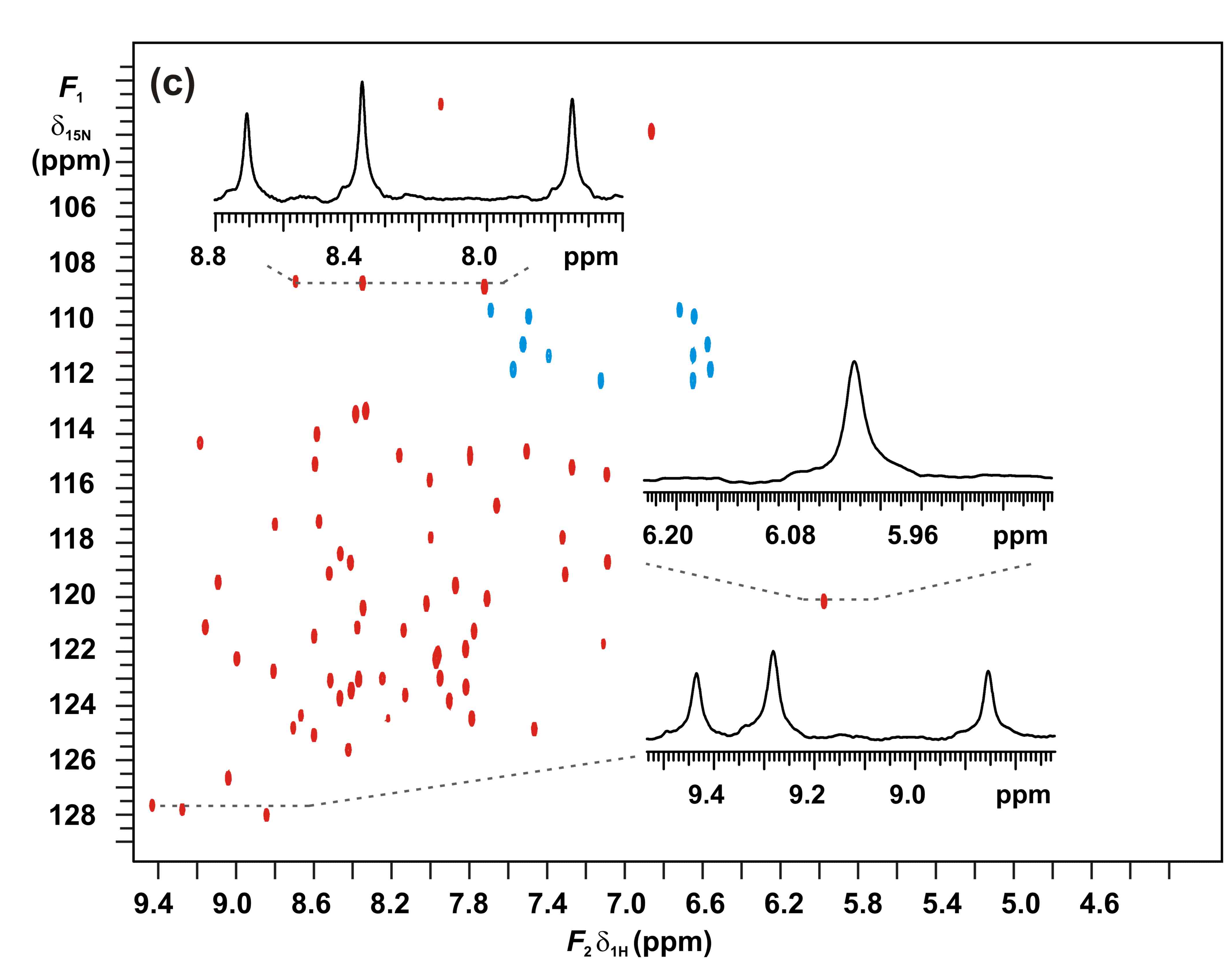

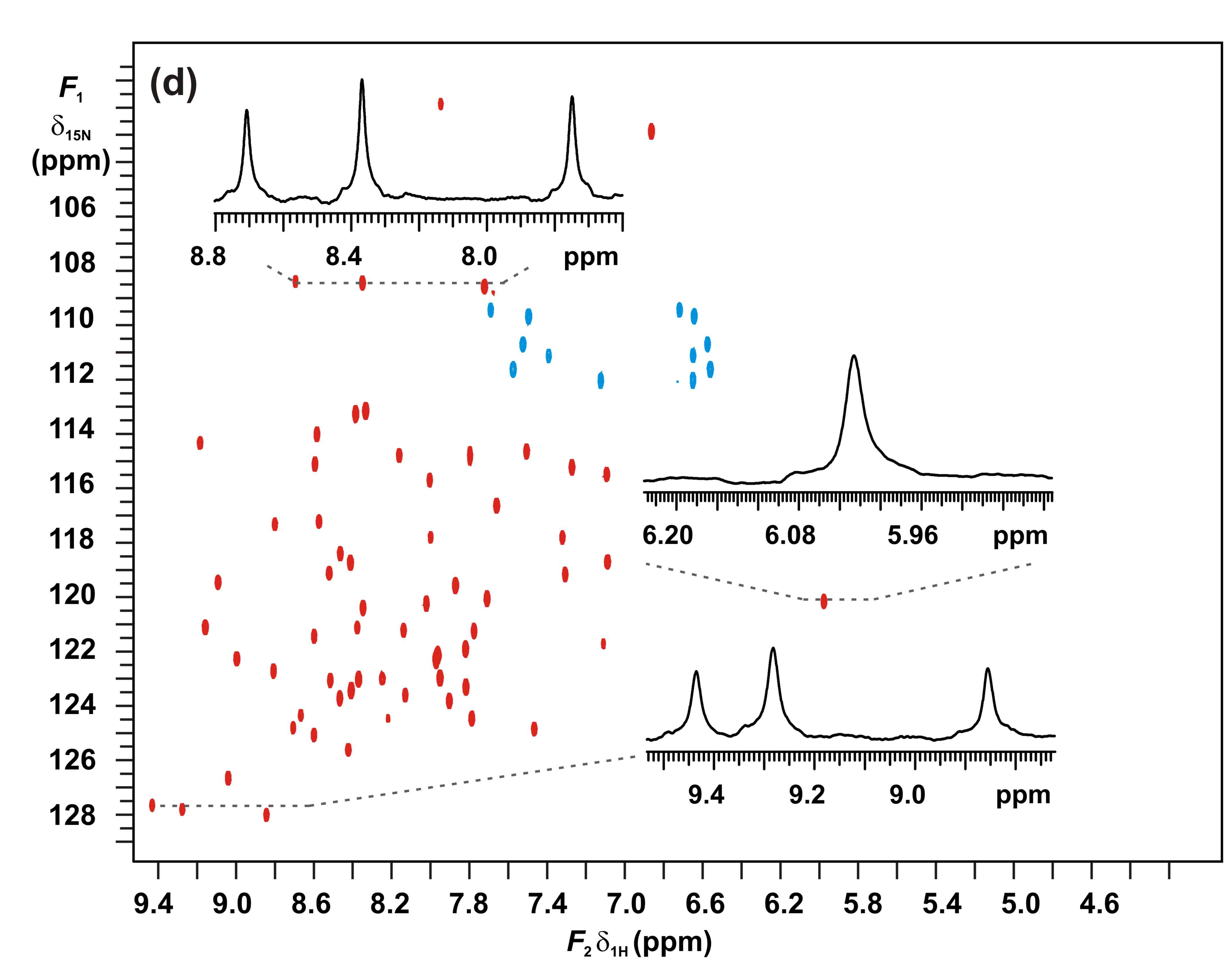


Supplementary Figure 8 ^1^H-^15^N-HSQC spectra of ^15^N-ubiquitin in 90% H_2_O / 10% D_2_O (2) acquired by (a) HSQC and real-time pure shift gBIRD-HSQC with (b) and without (c) chunk-to-chunk phase cycling of the J-refocusing element as suggested in (Ying 2014). These experiments were recorded by using the same 32 scans as for those shown on Supplementary Figure 3 and 4, but here only the first two steps of the phase cycling was used. In the case of (d) four steps were used from our phase cycling, which is more important than the MLEV type chunk-to-chunk phase cycling in our case. However, real-time pure shift experiments can be performed with the same minimal phase cycling as the normal HSQC if sensitivity limitation does not require more scans. See below a comparison for a selected resonance (I36). Insets are shown at δ_15N_ 108.84, 120.30 and 127.86 ppm, respectively.

**Pulse sequence code for Agilent/Varian spectrometers**

/*Pulse Sequence Code for real-time pure shift gHSQC in proteins using 15N-BIRD vs. BASHD vs homoDEC*/

/* was derived from LPKP_final_rtgHSQCBIRD_Suppl.c published in Angew.Chem.Int.Ed. 2013 (52) 11616-11619.*/

/*----------------------------------------------------------

Developed By NMR Group

School of Chemistry

University of Manchester

United Kingdom

Dec 2014

----------------------------------------------------------*/

/*User's Guide for experimental setup:

1. BIRD = 'n' selects conventional gHSQC

2. BIRD = 'n' and homo='y' selects traditional homodecoupling as implemented in VnmrJ 4.0

Create the required band-selective homodecoupling shape with Pbox (select the region and set the pulse length to 20ms; shape CAWURST)

set hdseq, hdpwr about more than 20dB than calculated by Pbox (26-36dB); hdmf

bear in mind that "hdof" does not supported for shaped homoDEC (see VnmrJ4.0 PSG object code)

-----------------------------------------------------------------------

3. BIRD = 'y' selects real-time pure shift gHSQC

Four options within BIRD are:

BIRDmode ='h' selects hard 15N inversion pulse during BIRD //works well for proteins because sw1 is small

BIRDmode ='b' selects shaped 15N inversion pulse during BIRD //recommended BIP shapes useful for sw1>5kHz;

(parameters to adjust: shp_HBIP, pw_HBIP, pwr_HBIP and shp_XBIP, pw_XBIP and pwr_XBIP)

(hard pulses usually good for proton thus recommended to set shp_HBIP='' pw_HBIP=pw90 pwr_HBIP=tpwr)

BIRDmode ='n' selects a 1H band-selective inversion pulse instead of BIRD-filter //applied for NH-region in proteins to reduce relaxation loss in real-time ACQ

(parameters to adjust: shp_NH, pw_NH, pwr_NH; the shape has to be phase modulated if not on-resonance, frequency jumps on the transmitter should be avoided during ACQ)

Note: npoints has to be an integer submultiple of np; set chunktime by setting npoints for a given sw then set np=$i*npoints where $i is the recquired number of loops for the total acquisition time

chunk_time=npoints/(2*sw)=at/cycles

cycles=np/npoints, an integer

at=np/(2*sw)=cycles*npoints/(2*sw)=cycles*chunk_time

--------------------------------------------------------------------------------------------------------------------------------*/

//tested on VNMRS console with VnmrJ4.0 and INOVA with VnmrJ2.2C

//the latter needs some modifications in the code, see comments at the beginning of ACQ period

/*

real time variables

v20 reserved for the loop (hardloop on INOVA, software loop on VNMRS)

v1-v4 phase cycle as used in standard HSQC code

v7-v9 phase cycle on the pure shift elements

v10 assigned to ct

*/

#include <standard.h>

//#include <chempack.h>

/*------------------------------------------

Phase tables for Varian gHSQC

--------------------------------------------*/

static int ph1[4] = {1,1,3,3}, //v1 - proton 90 at the end of first inept

ph2[2] = {0,2}, //v2 - X 90 at the end of first inept

ph3[8] = {0,0,0,0,2,2,2,2}, //v3 - proton 90 in 2nd inept

ph4[16] = {0,0,0,0,0,0,0,0,2,2,2,2,2,2,2,2}, //v4 - X 90 in 2nd inept

ph5[16] = {1,3,3,1,3,1,1,3,3,1,1,3,1,3,3,1}; //oph

/*------------------------------------------

Phase tables for rtgHSQC-BIRD

---------------------------------------------*/

static int ph11[8] = {1,1,1,1,3,3,3,3}, //v1

ph12[2] = {0,2}, //v2

ph13[16] = {0,0,0,0,0,0,0,0,2,2,2,2,2,2,2,2}, //v3

ph14[32] = {0,0,0,0,0,0,0,0,0,0,0,0,0,0,0,0,2,2,2,2,2,2,2,2,2,2,2,2,2,2,2,2}, //v4

ph15[32] = {1,3,1,3,3,1,3,1,3,1,3,1,1,3,1,3,3,1,3,1,1,3,1,3,1,3,1,3,3,1,3,1}; //oph

static int ph17[4] = {0,0,1,1}, //v7 - 1st 90 of bird and the hard 180

ph18[4] = {1,1,2,2}, //v8 - simpulse 180 of bird

ph19[4] = {2,2,3,3}; //v9 - 2nd 90 of bird

pulsesequence()

{

/*-------------------------------------------------------

DECLARE AND LOAD VARIABLES

----------------------------------------------------------*/

//HSQC part

double evolcorr=2.0*pw+4.0e-6,

tau = 1.0/(4.0*(getval("j1xh"))),

taug=2.0*tau,

mult = getval("mult");

int phase1 = (int)(getval("phase")+0.5),

ZZgsign=1.0,

icosel;

//BIRD

double d1_corr=0.0, j1xh = getval("j1xh"),

rof3=getval("rof3"), //delay for receiver off - can be zero if ddrpm='r'

tBal=getval("tBal"), //supports inova console if ~1/(fb*1.3)

pw_HBIP = getval("pw_HBIP"),

pw_XBIP = getval("pw_XBIP"),

pwr_HBIP = getval("pwr_HBIP"),

pwr_XBIP = getval("pwr_XBIP"),

pw_NH = getval("pw_NH"),

pwr_NH = getval("pwr_NH"),

//supports calibration of 1H and 15N pulses directly by using this HSQC sequence

calH = getval("calH"), //calibrate pw; array between 0.2 and 1.8 with steps of 0.2

calX = getval("calX"), //calibrate pwx; array between 0.2 and 1.8 with steps of 0.2

cal_pwlvl = getval("cal_pwlvl"),

cal_pwxlvl = getval("cal_pwxlvl"),

//array this variables instead of all pulses on obs/dec channels for better calibration curves

cal_pw = getval("cal_pw"), //if cal_pwlvl!=tpwr then use this with calH

cal_pwx = getval("cal_pwx"), //if cal_pwxlvl!=pwxlvl then use this with calX

gt1 = getval("gt1"), //CTP during ACQ for BIRD or NHsel

gzlvl1 = getval("gzlvl1"),

gt2 = getval("gt2"), //CTP during ACQ for hard 180

gzlvl2 = getval("gzlvl2"),

gstab = getval("gstab"), //gradient stabilization delay for CTP gradients during the pure shift elements

npoints=getval("npoints"), // npoints should be an integer multiple of np

cycles=np/npoints;

/*********************/

cycles = (double)((int)((cycles)));

initval(cycles,v20);

/*********************/

char shp_HBIP[MAXSTR], shp_XBIP[MAXSTR], shp_NH[MAXSTR],

lkgate_flg[MAXSTR], //lock gating functionality (not essential for this experiment)

homo[MAXSTR], //classic homodecoupling

gradaxis[MAXSTR]; //gradient axis during ACQ ("x"/"y"/"z"/"t" implement x/y/z/ or simultanous XY gradient pulses)

getstr("shp_HBIP",shp_HBIP);

getstr("shp_XBIP",shp_XBIP);

getstr("shp_NH",shp_NH);

getstr("lkgate_flg",lkgate_flg);

getstr("homo",homo);

getstr("gradaxis",gradaxis);

//gradients

double gtE = getval("gtE"), //HSQC encoding

gzlvlE = getval("gzlvlE"),

gtD = getval("gtD"), //HSQC decoding

gzlvlD = getval("gzlvlD"),

hsglvl = getval("hsglvl"),

hsgt = getval("hsgt"),

hsgstab = getval("hsgstab");

char BIRD[MAXSTR], // Flag to choose normal HSQC or pure-shift ('n'/'y')

BIRDmode[MAXSTR]; //Flag to choose hard-BIRD/bip-BIRD/BASHD ('h'/'b'/'n')

getstr("BIRD",BIRD);

getstr("BIRDmode",BIRDmode);

char sspul[MAXSTR], nullflg[MAXSTR], PFGflg[MAXSTR];

getstr("sspul",sspul);

getstr("nullflg",nullflg);

getstr("PFGflg",PFGflg);

//d1 correction was normalized to BIRDmode='b' and supports HSQC/bip-BIRD/NHsel

if (BIRD[0]=='n') d1_corr=(np/npoints)*(pw*4.0+1.0/j1xh+pw_XBIP+4.0e-5+2.0*(gt1+gt2+2.0*gstab)+8.0*rof1);

if ((BIRD[0]=='y') && (BIRDmode[0]=='n')) d1_corr=(np/npoints)*(4.0*rof1+(pw*4.0+1.0/j1xh+pw_XBIP+4.0e-5+2.0*(gt1+gt2+2.0*gstab))-(pw_NH+2.0*5.0e-5+(pw*2.0+4.0e-5)+2.0*(gt1+gt2+2.0*gstab)));

if ((BIRD[0]=='y') && (BIRDmode[0]=='b')) d1_corr=0.0;

//check to avoid dangerous sample heating by RF and GRD

if (d1_corr > 0.20*d1)

{

abort_message("Correction for relaxation delay is more than 20 percent! Check ACQ parameters...\n");

}

//evolcorr and mult declarations

evolcorr = 2*pw+4.0e-6;

if (mult > 0.5)

taug = 2*tau;

else

taug = gtE + gstab + 2*GRADIENT_DELAY;

ZZgsign=-1;

if (mult == 2) ZZgsign=1;

icosel = 1;

//setup the phase cycle

assign(ct,v10);

if (BIRD[0]=='n')

{

//gHSQC phases

settable(t1,4,ph1);

settable(t2,2,ph2);

settable(t3,8,ph3);

settable(t4,16,ph4);

settable(t5,16,ph5);

}

else

{

//extended phase cycling for pure shift

settable(t1,8,ph11);

settable(t2,2,ph12);

settable(t3,16,ph13);

settable(t4,32,ph14);

settable(t5,32,ph15);

settable(t7,4,ph17);

settable(t8,4,ph18);

settable(t9,4,ph19);

getelem(t7, v10, v7);

getelem(t8, v10, v8);

getelem(t9, v10, v9);

}

getelem(t1, v10, v1);

getelem(t2, v10, v2);

getelem(t3, v10, v3);

getelem(t4, v10, v4);

getelem(t5, v10, oph);

initval(2.0*(double)(((int)(d2*getval("sw1")+0.5)%2)),v5);

if ((phase1 == 2) || (phase1 == 5))

icosel = -1;

add(v2,v5,v2);

add(oph,v5,oph);

/* BEGIN PULSE SEQUENCE */

status(A);

//correct the total experiment time for HSQC-ref experiment

if ((d1_corr>0.0) && (BIRD[0]=='n')) delay(d1_corr);

if ((d1_corr>0.0) && (BIRD[0]=='y') && (BIRDmode[0]=='n')) delay(d1_corr);

if (sspul[A] == 'y')

{

//lock gating

if (lkgate_flg[0] == 'y') lk_hold(); /* turn lock sampling off */

if (PFGflg[A] == 'y')

{

obspower(tpwr);

delay(5.0e-5);

if (hsgt>0.0) { zgradpulse(hsglvl,hsgt); }

rgpulse(pw,zero,rof1,rof1);

if (hsgt>0.0) { zgradpulse(hsglvl,hsgt); }

}

else

{

obspower(tpwr-12);

delay(5.0e-5);

rgpulse(500*pw,zero,rof1,rof1);

rgpulse(500*pw,one,rof1,rof1);

}

}

if (nullflg[0]=='y') obspower(tpwr);

else obspower(cal_pwlvl);

decpower(pwxlvl);

txphase(zero);

decphase(zero);

obsoffset(tof);

decoffset(dof);

if (lkgate_flg[0] == 'y') lk_sample(); /* turn lock sampling on */

delay(d1);

if (lkgate_flg[0] == 'y') lk_hold(); /* turn lock sampling off */

delay(5.0e-5);

status(B);

/****** null flag starts here *****/

if (getflag("nullflg"))

{

rgpulse(0.5*pw,zero,rof1,rof1);

txphase(zero);

delay(2.0*tau);

simpulse(2.0*pw,2.0*pwx,zero,zero,rof1,rof1);

txphase(two);

delay(2.0*tau);

rgpulse(1.5*pw,two,rof1,rof1);

txphase(zero);

// purge gradient

if (hsgt>0.0) { zgradpulse(hsglvl,hsgt); }

if (cal_pwlvl!=tpwr) obspower(cal_pwlvl);

delay(hsgstab);

}

//option to proton calibrations

rgpulse(cal_pw*calH,zero,rof1,rof1);

if (cal_pwlvl!=tpwr) obspower(tpwr);

delay(tau);

simpulse(2.0*pw,2.0*pwx,zero,zero,rof1,rof1);

txphase(v1);

delay(tau);

rgpulse(pw,v1,rof1,rof1);

// purge gradient

if (hsgt>0.0) { zgradpulse(hsglvl,2.0*hsgt); }

decphase(v2);

if (cal_pwxlvl!=pwxlvl) decpower(cal_pwxlvl);

delay(hsgstab);

//option to pwx calibrations

decrgpulse(cal_pwx*calX, v2, rof1, 2.0e-6);

txphase(zero);

decphase(zero);

if (cal_pwxlvl!=pwxlvl) decpower(pwxlvl);

delay(d2/2.0); // First half of t1 evolution

rgpulse(2.0*pw,zero,2.0e-6,2.0e-6);

delay(d2/2.0); // Second half of t1 evolution

zgradpulse(gzlvlE,gtE);

delay(taug - gtE - 2.0*GRADIENT_DELAY);

simpulse(mult*pw,2.0*pwx,zero,zero,rof1,rof1);

delay(taug + evolcorr);

decrgpulse(pwx,v4,2.0e-6,rof1);

// purge gradient

if (hsgt>0.0) { zgradpulse(ZZgsign*0.6*hsglvl,1.2*hsgt); }

txphase(v3);

delay(hsgstab);

rgpulse(pw,v3,rof1,rof1);

delay(tau - (2.0*pw/PI) - 2.0*rof1);

simpulse(2.0*pw,2.0*pwx,zero,zero,rof1,rof1);

zgradpulse(icosel*gzlvlD,gtD);

decpower(dpwr);

delay(tau - gtD - 2.0*GRADIENT_DELAY - POWER_DELAY);

/************************Acquisition starts here**************************/

delay(tBal);

//filter delay (Hoult) for inova; adjust tBal manually for the same effect

//tBAL=(1.0/(getval("fb")*1.3)) gives good results

setacqmode(WACQ|NZ); //use this line only for vnmrs console; comment this out in case of inova; that is the only change required to use the sequence with inova

obsblank();

delay(rof2);

startacq(alfa);

//Observe the 1st half chunk

if (BIRD[0]=='y')

{

status(C);

acquire(npoints/2.0,1.0/sw);

rcvroff();

status(B);

obspower(tpwr);

txphase(v7);

// optional CTP gradient

if (gt1>0.0)

{

if (gradaxis[0]=='t')

{

rgradient('x',gzlvl1);

rgradient('y',gzlvl1);

delay(gt1);

rgradient('x',0.0);

rgradient('y',0.0);

}

else {

if ((gradaxis[0]=='x') || (gradaxis[0]=='y'))

{

rgradient(gradaxis[0],gzlvl1);

delay(gt1);

rgradient(gradaxis[0],0.0);

}

else zgradpulse(gzlvl1,gt1); }

delay(gstab);

}

//hard pulses on all channels in BIRD

if (BIRDmode[0]== 'h')

{

delay(2.0*pwx+2.0*rof1);

rgpulse(pw,v7,rof1,rof1);

decpower(pwxlvl);

delay(2.0*tau);

simpulse(2.0*pw,2.0*pwx,v8,v8,rof1,rof1);

delay(2.0*tau);

rgpulse(pw,v9,rof1,rof1);

decrgpulse(2.0*pwx,v8,rof1,rof1);

}

//shaped180 pulses on all channels in BIRD

if (BIRDmode[0]== 'b')

{

delay(pw_XBIP+2.0*rof1);

rgpulse(pw,v7,rof1,rof1);

if (pwr_HBIP!=tpwr) obspower(pwr_HBIP);

if (pwr_XBIP!=pwxlvl) decpower(pwr_XBIP); else decpower(pwxlvl);

delay(2.0*tau);

simshaped_pulse(shp_HBIP,shp_XBIP,pw_HBIP,pw_XBIP,v8,v8,rof1,rof1);

if (pwr_HBIP!=tpwr) obspower(tpwr);

delay(2.0*tau);

rgpulse(pw,v9,rof1,rof1);

decshaped_pulse(shp_XBIP,pw_XBIP,v8,rof1,rof1);

}

//BASHD method for proteins

if (BIRDmode[0]== 'n')

{

if (pwr_NH!=tpwr) obspower(pwr_NH);

delay(5.0e-5);

shaped_pulse(shp_NH,pw_NH,v7,rof1,rof1);

if (pwr_NH!=tpwr) obspower(tpwr);

delay(5.0e-5);

}

// optional CTP gradient

if (gt1>0.0)

{

if (gradaxis[0]=='t')

{

rgradient('x',gzlvl1);

rgradient('y',gzlvl1);

delay(gt1);

rgradient('x',0.0);

rgradient('y',0.0);

}

else {

if ((gradaxis[0]=='x') || (gradaxis[0]=='y'))

{

rgradient(gradaxis[0],gzlvl1);

delay(gt1);

rgradient(gradaxis[0],0.0);

}

else zgradpulse(gzlvl1,gt1); }

delay(gstab);

}

delay(2.0e-5);

// optional CTP gradient

if (gt2>0.0)

{

if (gradaxis[0]=='t')

{

rgradient('x',gzlvl2);

rgradient('y',gzlvl2);

delay(gt2);

rgradient('x',0.0);

rgradient('y',0.0);

}

else {

if ((gradaxis[1]=='x') || (gradaxis[1]=='y'))

{

rgradient(gradaxis[1],gzlvl2);

delay(gt2);

rgradient(gradaxis[1],0.0);

}

else zgradpulse(gzlvl2,gt2); }

delay(gstab);

}

// hard180 proton pulse

rgpulse(2.0*pw,v7,rof1,rof1);

// optional CTP gradient

if (gt2>0.0)

{

if (gradaxis[0]=='t')

{

rgradient('x',gzlvl2);

rgradient('y',gzlvl2);

delay(gt2);

rgradient('x',0.0);

rgradient('y',0.0);

}

else {

if ((gradaxis[1]=='x') || (gradaxis[1]=='y'))

{

rgradient(gradaxis[1],gzlvl2);

delay(gt2);

rgradient(gradaxis[1],0.0);

}

else zgradpulse(gzlvl2,gt2); }

delay(gstab);

}

// change DEC power

decpower(dpwr);

obsblank();

delay(1.0e-5);

rcvron(); //this includes rof3

delay(1.0e-5);

decr(v20);

//more chunks

starthardloop(v20);

status(C);

acquire(npoints,1.0/sw);

rcvroff();

status(B);

obspower(tpwr);

txphase(v7);

// optional CTP gradient

if (gt1>0.0)

{

if (gradaxis[0]=='t')

{

rgradient('x',gzlvl1);

rgradient('y',gzlvl1);

delay(gt1);

rgradient('x',0.0);

rgradient('y',0.0);

}

else {

if ((gradaxis[0]=='x') || (gradaxis[0]=='y'))

{

rgradient(gradaxis[0],gzlvl1);

delay(gt1);

rgradient(gradaxis[0],0.0);

}

else zgradpulse(gzlvl1,gt1); }

delay(gstab);

}

//hard pulses on all channels in BIRD

if (BIRDmode[0]== 'h')

{

delay(2.0*pwx+2.0*rof1);

rgpulse(pw,v7,rof1,rof1);

decpower(pwxlvl);

delay(2.0*tau);

simpulse(2.0*pw,2.0*pwx,v8,v8,rof1,rof1);

delay(2.0*tau);

rgpulse(pw,v9,rof1,rof1);

decrgpulse(2.0*pwx,v8,rof1,rof1);

}

//shaped180 pulses on all channels in BIRD

if (BIRDmode[0]== 'b')

{

delay(pw_XBIP+2.0*rof1);

rgpulse(pw,v7,rof1,rof1);

if (pwr_HBIP!=tpwr) obspower(pwr_HBIP);

if (pwr_XBIP!=pwxlvl) decpower(pwr_XBIP); else decpower(pwxlvl);

delay(2.0*tau);

simshaped_pulse(shp_HBIP,shp_XBIP,pw_HBIP,pw_XBIP,v8,v8,rof1,rof1);

if (pwr_HBIP!=tpwr) obspower(tpwr);

delay(2.0*tau);

rgpulse(pw,v9,rof1,rof1);

decshaped_pulse(shp_XBIP,pw_XBIP,v8,rof1,rof1);

}

//BASHD method for proteins

if (BIRDmode[0]== 'n')

{

if (pwr_NH!=tpwr) obspower(pwr_NH);

delay(5.0e-5);

shaped_pulse(shp_NH,pw_NH,v7,rof1,rof1);

if (pwr_NH!=tpwr) obspower(tpwr);

delay(5.0e-5);

}

// optional CTP gradient

if (gt1>0.0)

{

if (gradaxis[0]=='t')

{

rgradient('x',gzlvl1);

rgradient('y',gzlvl1);

delay(gt1);

rgradient('x',0.0);

rgradient('y',0.0);

}

else {

if ((gradaxis[0]=='x') || (gradaxis[0]=='y'))

{

rgradient(gradaxis[0],gzlvl1);

delay(gt1);

rgradient(gradaxis[0],0.0);

}

else zgradpulse(gzlvl1,gt1); }

delay(gstab);

}

delay(2.0e-5);

// optional CTP gradient

if (gt2>0.0)

{

if (gradaxis[0]=='t')

{

rgradient('x',gzlvl2);

rgradient('y',gzlvl2);

delay(gt2);

rgradient('x',0.0);

rgradient('y',0.0);

}

else {

if ((gradaxis[1]=='x') || (gradaxis[1]=='y'))

{

rgradient(gradaxis[1],gzlvl2);

delay(gt2);

rgradient(gradaxis[1],0.0);

}

else zgradpulse(gzlvl2,gt2); }

delay(gstab);

}

// hard180 proton pulse

rgpulse(2.0*pw,v7,rof1,rof1);

// optional CTP gradient

if (gt2>0.0)

{

if (gradaxis[0]=='t')

{

rgradient('x',gzlvl2);

rgradient('y',gzlvl2);

delay(gt2);

rgradient('x',0.0);

rgradient('y',0.0);

}

else {

if ((gradaxis[1]=='x') || (gradaxis[1]=='y'))

{

rgradient(gradaxis[1],gzlvl2);

delay(gt2);

rgradient(gradaxis[1],0.0);

}

else zgradpulse(gzlvl2,gt2); }

delay(gstab);

}

// change DEC power

decpower(dpwr);

obsblank();

delay(1.0e-5);

rcvron(); //this includes rof3

delay(1.0e-5);

endhardloop();

//last half chunk

status(C);

acquire(npoints/2.0,1.0/sw);

rcvroff();

endacq();

incr(v20);

if (lkgate_flg[0] == 'y') lk_sample(); /* turn lock sampling on */

}

// ACQ for conventional gHSQC

else

{

if (homo[A]=='y')

{ status(C); }

else

{

status(C);

acquire(np,1.0/sw);

rcvroff();

if (lkgate_flg[0] == 'y') lk_sample(); /* turn lock sampling on */

}

}

}

**Parameter file for Agilent/Varian spectrometers**

il 2 2 1 0 0 2 1 0 1 64

1 "n"

2 "y" "n"

dmm2 4 2 4 0 0 2 1 0 1 64

1 "ccp"

9 "c" "f" "g" "m" "p" "r" "u" "w" "x"

CSshape 2 2 8 0 0 2 1 0 1 64

1 "gexp"

0

CSlines 7 1 32767 0 0 2 1 0 1 64

1 4

0

CS_inf 4 2 4 0 0 3 1 0 1 64

1 "y"

0

BIRDmode 4 2 4 0 0 2 1 0 1 64

1 "n"

0

Archive1 2 2 8 0 0 2 1 0 1 64

1 ""

0

ACQtime 7 1 100000000 0 1 2 1 256 1 64

1 1189.103942

0

BIRD 4 2 4 0 0 2 1 0 1 64

1 "y"

0

CS_dpars 4 2 4 0 0 3 1 0 1 64

1 "n"

0

CS_cln 4 2 4 0 0 3 1 0 1 64

1 "y"

0

CSdomain 7 1 32767 0 0 2 1 0 1 64

1 1

0

CSdensity 1 1 100 0 0 2 1 0 1 64

1 100

0

CS_se 4 2 4 0 0 3 1 0 1 64

1 "y"

0

CSdnoise 4 2 4 0 0 3 1 0 1 64

1 "y"

0

CSinp 2 2 8 0 0 2 1 0 1 64

1 "n"

0

CSprosize 7 1 32767 0 0 2 1 256 1 64

3 0 0 0

0

CSpars 2 2 8 0 0 2 1 256 1 64

1 ""

0

CSniIndex 7 1 32767 0 0 2 1 256 1 64

1 0

0

CSni2Index 7 1 32767 0 0 2 1 256 1 64

1 0

0

CSprofile 2 2 8 0 0 2 1 256 1 64

3 ""

""

""

0

CSreconmethod 2 2 8 0 0 3 1 0 1 64

1 "CLEAN"

0

CSseed 1 1 9.99999984307e+17 -9.99999984307e+17 0 2 1 0 1 64

1 169

0

H1reffrq 1 1 9.99999984307e+17 -9.99999984307e+17 0 2 1 0 1 64

1 499.82345741

0

CStype 4 2 4 0 0 2 1 0 1 64

1 "a"

0

CSsp 7 1 32767 0 0 3 1 0 1 64

1 0

0

CSshowpro 4 2 4 0 0 2 1 0 1 64

1 "y"

0

CSshowsch 4 2 4 0 0 2 1 0 1 64

1 "y"

0

CSthr 1 1 100 1 0.1 3 1 0 1 64

1 2

0

CSwt 4 2 4 0 0 2 1 0 1 64

1 "n"

0

CSwp 7 1 32767 0 0 3 1 0 1 64

1 0

0

Qgain 1 1 9.99999984307e+17 -9.99999984307e+17 0 2 1 0 1 64

1 30

0

MinSW 2 2 8 0 0 3 1 0 1 64

1 "skip"

4 "off" "auto" "manual" "skip"

PFGflg 4 2 4 0 0 2 1 0 1 64

1 "y"

0

Qinsref 1 1 9.99999984307e+17 -9.99999984307e+17 0 2 1 0 1 64

1 7.4912e-05

0

Qins 1 1 9.99999984307e+17 -9.99999984307e+17 0 2 1 0 1 64

1 80

0

Qmult 1 1 9.99999984307e+17 -9.99999984307e+17 0 2 1 0 1 64

1 0.5

0

aig 2 2 2 0 0 4 1 1 1 64

1 "ai"

2 "nm" "ai"

acqstatus 7 1 32767 0 0 2 1 256 1 64

2 101 0

0

acqdim 7 1 32767 0 0 2 1 0 1 64

1 2

0

accord 4 2 4 0 0 2 1 0 1 64

1 "y"

0

acqcycles 1 1 9.99999984307e+17 -9.99999984307e+17 0 2 1 0 1 64

1 128

0

acqpath 2 2 8 0 0 2 1 0 1 64

1 ""

0

adir2tmplt 2 2 8 0 0 2 1 0 1 64

1 ""

0

actionid 2 2 8 0 0 4 1 0 1 64

1 "null"

0

alock 2 2 8 0 0 2 1 0 1 64

1 "n"

5 "a" "n" "s" "u" "y"

alfa 6 1 8190 0 0.4 2 1 2 1 64

1 10

0

ap 2 2 1023 0 0 4 1 6 1 64

1 "1:SAMPLE:date,solvent,sample;1:ACQUISITION:sw:1,at:3,np:0,fb:0,ss(ss):0,d1:3,nt:0;1:2D ACQUISITION:sw1:1,ni,d2(d2):6,phase;1:PRESATURATION:satmode,wet;1:TRANSMITTER:tn,sfrq:3,tof:1,tpwr:0,pw:3;1:DECOUPLER:dn,dof:1,dm,decwave,dmf,dpwr,pwxlvl,pwx:3;1:HSQC:j1xh:1,nullflg,mult;2:FLAGS:hs,sspul,PFGflg,hsglvl;2:SPECIAL:temp:1,gain:0,spin:0;2:GRADIENTS:gzlvlE,gtE:6,EDratio:3,gstab:6;2:F2 PROCESSING:lb(lb):2,sb(sb):3,sbs(sb):3,gf(gf):3,gfs(gf):3,awc(awc):3,lsfid(lsfid):0,phfid(phfid):1,proc(proc<>'ft'),fn;2:F1 PROCESSING:lb1(lb1):2,sb1(sb1):3,sbs1(sb1):3,gf1(gf1):3,gfs1(gf1):3,awc1(awc1):3,proc1(proc1<>'ft'),fn1:0;2:DISPLAY:sp:1,wp:1,sp1:1,wp1:1,rfl:1,rfp:1,rfl1:1,rfp1:1;2:PLOT:wc:1,sc:1,wc2:1,sc2:1,vs:0,th:0,aig*,dcg*,dmg*;"

0

apptype 2 2 8 0 0 2 1 1 1 64

1 "hetero2D"

0

arraydim 7 1 32768 1 1 2 1 5 1 64

1 128

0

arraydelta 7 1 32767 0 0 4 1 0 1 64

1 1

0

array 2 2 256 0 0 2 1 1 1 64

1 "phase"

0

arraydodc 4 2 4 0 0 4 1 0 1 64

1 "n"

0

at 1 1 14 14 14 2 1 8203 1 64

1 0.192

0

arraymax 7 1 32767 0 0 4 1 0 1 64

1 128

0

arrayflip 4 2 4 0 0 4 1 0 1 64

1 "n"

0

arrayelemts 1 1 9.99999984307e+17 -9.99999984307e+17 0 2 1 0 1 64

1 2

0

arraystop 7 1 32767 0 0 4 1 0 1 64

1 62

0

arraystart 7 1 32767 0 0 4 1 0 1 64

1 62

0

arraywhitewash 4 2 4 0 0 4 1 0 1 64

1 "n"

0

axis 4 2 4 0 0 4 1 0 1 64

1 "pd"

11 "c" "d" "1" "2" "3" "h" "k" "m" "n" "p" "u"

awc 1 1 1 -1 0.001 3 1 1 0 64

1 0

0

avwdir 2 2 8 0 0 3 1 0 1 64

1 ""

0

awc1 1 1 1 -1 0.001 3 1 1 0 64

1 0

0

axisf 4 2 4 0 0 4 1 0 1 64

1 "s"

4 "m" "n" "s" "u"

bs 7 1 32767 0 1 2 1 0 1 64

1 4

0

ct 7 1 1000000000 0 1 2 1 6 1 64

1 32

0

cf 7 1 32767 0 1 3 1 1 0 64

1 1

0

cal_pwx 6 1 13 13 13 2 1 8192 1 64

1 47

0

cal_pw 6 1 13 13 13 2 1 8192 1 64

1 9.6

0

calX 1 1 9.99999984307e+17 -9.99999984307e+17 0 2 1 0 1 64

1 1

0

calH 1 1 9.99999984307e+17 -9.99999984307e+17 0 2 1 0 1 64

1 1

0

cal_pwlvl 7 1 32767 0 0 2 1 0 1 64

1 61

0

cal_pwxlvl 7 1 32767 0 0 2 1 0 1 64

1 58

0

celem 1 1 9.99999984307e+17 -9.99999984307e+17 0 2 1 0 1 64

1 128

0

compshape 2 2 8 0 0 2 1 0 1 64

1 "hard"

0

comment 2 2 8 0 0 3 1 0 1 64

1 ""

0

cmult 1 1 2 0 0 2 1 0 1 64

1 1

0

composit 4 2 4 0 0 2 1 0 1 64

1 "n"

0

cppauseflg 4 2 4 0 0 4 1 0 1 64

1 "n"

0

console 2 2 8 0 0 2 1 0 1 64

1 "vnmrs"

0

cp 2 2 1 0 0 2 1 0 1 64

1 "y"

2 "y" "n"

cr1 1 1 1000000000 -1000000000 0 4 1 3 1 64

1 6426.23215334

0

cr 1 1 1000000000 -1000000000 0 4 1 1 1 64

1 4693.42540637

0

crf 1 1 9.99999984307e+17 -9.99999984307e+17 0 4 1 0 1 64

1 0

0

dcrmv 4 2 9.99999984307e+17 -9.99999984307e+17 0 4 1 0 1 64

1 "n"

2 "y" "n"

date 2 2 9 0 0 2 1 1 1 64

1 "Sep 8 2014"

0

d1 3 1 14 14 14 2 1 8194 1 64

1 3

0

cutoff 1 1 9.99999984307e+17 -9.99999984307e+17 0 4 1 0 0 64

1 200

0

customflag 4 2 4 0 0 4 1 0 1 64

1 "n"

0

dataProcessed 1 1 9.99999984307e+17 -9.99999984307e+17 0 3 1 0 1 64

1 0

0

d2 3 1 14 14 14 2 1 8194 1 64

1 0

0

dataid 2 2 8 0 0 3 1 0 1 64

1 ""

0

dbwwet 1 1 9.99999984307e+17 -9.99999984307e+17 0 2 1 0 1 64

1 5000

0

datname 2 2 8 0 0 3 1 0 1 64

1 ""

0

dcg 2 2 3 0 0 4 1 1 1 64

1 ""

2 "dc" "cdc"

decwavewet 2 2 8 0 0 2 1 0 1 64

1 "w"

0

ddrtc 6 1 1000 0 0.4 2 1 0 1 64

1 34.8

0

ddrpm 2 2 8 0 0 2 1 0 1 64

1 "r"

0

deltaf 1 1 100 0 0 4 1 0 1 64

1 0.0206321094312

0

delta 1 1 5000000 0 0 4 1 1 1 64

1 198.364257812

0

delta1 1 1 5000000 0 0 4 1 3 1 64

1 70.3125

0

dfrq 5 1 1000000000 -1000000000 0 2 1 8 1 64

1 50.6526247

0

displaymanual 2 2 8 0 0 2 1 0 1 64

1 "basics"

0

dg2 2 2 8 0 0 4 1 4 1 64

6 "1:1st DECOUPLING:dfrq:3,dn,dpwr:0,dof:1,dm,dmm,dmf:0,dseq,dres:1,homo;"

"2(numrfch>2):2nd DECOUPLING:dfrq2:3,dn2,dpwr2:0,dof2:1,dm2,dmm2,dmf2:0,dseq2,dres2:1,homo2;"

"2(numrfch>3):3rd DECOUPLING:dfrq3:3,dn3,dpwr3:0,dof3:1,dseq3,dres3:1,homo3;"

"3(ni2):3D ACQUISITION:d3:3,sw2:1,ni2:0,phase2:0;"

"3(ni2):3D DISPLAY:rp2:1,lp2:1;"

"4(ni2):3D PROCESSING:lb2:3,sb2:3,sbs2(sb2):3,gf2:3,gfs2(gf2):3,awc2:3,wtfile2,proc2,fn2:0;"

0

dg 2 2 1023 0 0 4 1 6 1 64

1 "1:ACQUISITION:sw:1,at:3,np:0,bs(bs):0,ss(ss):0,d1:3,d2(d2):3,nt:0,ct:0;1:2D ACQUISITION:sw1:1,ni,phase:0;1:PRESATURATION:satmode,wet;1:Details in dgss:;2:TRANSMITTER:tn,sfrq:3,tof:1,tpwr:0,pw:3;2:DECOUPLER:dn,dof:1,pwxlvl:0,pwx:3,dm,decwave,dmf:0,dpwr:0;3:HSQC:j1xh:1,nullflg,mult;3:GRADIENTS:gzlvlE,gtE:6,EDratio:3,gstab:6,hsglvl,hsgt:6;3:SPECIAL:temp:1,spin,gain,sspul,pw90:3;4:PROCESSING:cf(nf):0,lb(lb):2,sb(sb):3,sbs(sb):3,gf(gf):3,gfs(gf):3,awc(awc):3,proc(proc<>'ft'),fn:0,math(math<>'f');4:2D PROCESSING:lb1(lb1):2,sb1(sb1):3,sbs1(sb1):3,gf1(gf1):3,gfs1(gf1):3,awc1(awc1):3,proc1(proc1<>'ft'),fn1:0,pmode;4:SAMPLE:date,solvent,sample;"

0

dg1 2 2 1023 0 0 4 1 6 1 64

1 "1:DISPLAY:sp:1,wp:1,vs:0;1:REFERENCE:rfl:1,rfp:1,cr:1,delta:1;1:PHASE:lp:1,rp:1,rp1(ni):1,lp1(ni):1;2:CHART:sc:0,wc:0,hzmm:2,vp:0,axis,pltmod,,th:0,,ho:2,vo:2,,trace:2;3(ni):2D:sp1:1,wp1:1,sc2:0,wc2:0,rfl1:1,rfp1:1;3:FLAGS:aig*,dcg*,dmg*;3:FID:sf:3,wf:3,vf:0;4:INTEGRAL:intmod,is:2,ins:3,io:0,,lvl:3,tlt:3;"

0

dgssm 2 2 8 0 0 4 1 6 1 64

1 "1:WET:wet,gzlvlw,gtw:6,gswet:6,dz:6;1:C13 WET:c13wet,dofwet:1,dpwrwet,dmfwet;2:WETshapes:wetshape1,wetshape2,wetshape3,wetshape4;3:WETpowers:wetpwr1,wetpwr2,wetpwr3,wetpwr4;3:WETpulses:pwwet1:3,pwwet2:3,pwwet3:3,pwwet4:3;4:PRESAT:satmode,satpwr,satdly:3,satfrq:1;"

0

dgs 2 2 1023 0 0 4 1 6 1 64

5 "1:AXIAL SHIMS:z1:0,z2:0,z3:0,z4:0,z5:0,z6:0,z7:0;"

"2:NON AXIAL SHIMS:x1:0,y1:0,xz:0,yz:0,xy:0,x2y2:0,x3:0,y3:0,xz2:0,yz2:0,zxy:0,zx2y2:0,z3x:0,z3y:0,z2x2y2:0,z2xy:0;"

"3:NON AXIAL SHIMS:zx3:0,zy3:0,x4:0,y4:0,z4x:0,z4y:0,z3x2y2:0,z3xy:0,z2x3:0,z2y3:0,z3x3:0,z3y3:0,z4x2y2:0,z4xy:0,z5x:0,z5y:0;"

"4:AUTOMATION:method,wshim,load,,spin:0,gain:0,alock,loc:0;"

"4:SPECIAL:temp;"

0

dglp 2 2 8 0 0 4 1 7 1 64

1 "1(lpfilt):LP:lpalg,lpopt,lpfilt:0,lpnupts:0,strtlp:0,lpext:0,strtext:0;2(lpfilt1):LP 1:lpalg1,lpopt1,lpfilt1:0,lpnupts1:0,strtlp1:0,lpext1:0,strtext1:0;3(lpfilt2):LP 2:lpalg2,lpopt2,lpfilt2:0,lpnupts2:0,strtlp2:0,lpext2:0,strtext2:0;4:LP OUTPUT:lpprint(lpfilt):0,lptrace(lpfilt):0,lpprint1(lpfilt1):0,lptrace1(lpfilt1):0,lpprint2(lpfilt2):0,lptrace2(lpfilt2):0;"

0

dgss 2 2 8 0 0 4 1 6 1 64

1 "1:WET:wet,wetshape,wetpwr,pwwet:3,composit,compshape,gzlvlw,gtw:6,gswet:6,dz:6;2:C13 WET:c13wet,dofwet:1,dpwrwet,dmfwet,decwavewet;4:PRESAT:satmode,satpwr,satdly:3,satfrq:1;"

0

dhp 1 1 9 9 9 2 1 8192 0 64

1 40

0

disCmd 2 2 8 0 0 4 1 0 1 64

1 ""

0

dmf 7 1 11 11 11 2 1 8194 1 64

1 16949

0

dm 4 2 4 0 0 2 1 0 1 64

1 "nny"

4 "a" "n" "s" "y"

dlp 7 1 10 10 10 2 1 8194 0 64

1 10

0

displaymode 4 2 9.99999984307e+17 -9.99999984307e+17 0 4 1 0 1 64

1 "r"

0

dmg1 2 2 8 0 0 4 1 0 1 64

1 "ph1"

4 "" "av1" "ph1" "pwr1"

dmg 2 2 2 0 0 4 1 3 1 64

1 "ph"

0

dmm 4 2 4 0 0 2 1 0 1 64

1 "ccp"

9 "c" "f" "g" "m" "p" "r" "u" "w" "x"

gfs 1 1 1000 -1000 0.001 3 1 1 0 64

1 0

0

dn 2 2 4 0 0 2 1 8 1 64

1 "N15"

0

dod 3 1 14 14 14 2 1 8194 1 64

1 0.00999999977648

0

dnref 2 2 8 0 0 4 1 1 1 64

1 "Me4Si, 1% in CDCl3"

0

dof 5 1 8 8 8 2 5 8202 1 64

1 990

0

dotflag 2 2 8 0 0 4 1 0 1 64

1 "n"

2 "n" "y"

doscout 7 1 10 -10 1 2 1 0 1 64

1 0

0

dp 2 2 1 0 0 2 1 0 1 64

1 "y"

2 "y" "n"

dpwr 1 1 9 9 9 2 1 8194 1 64

1 46

0

dpf_wc2 1 1 9.99999984307e+17 -9.99999984307e+17 0 4 1 0 1 64

1 300

0

dreffrq 1 1 9.99999984307e+17 -9.99999984307e+17 0 2 1 0 1 64

1 50.6466710947

0

dres 7 1 360 1 0 2 1 0 1 64

1 9

0

dss_wc2 1 1 9.99999984307e+17 -9.99999984307e+17 0 4 1 0 1 64

1 151.75

0

dseq 2 2 8 0 0 2 1 0 1 64

1 "kp_bioN15_Ch3_10kHz-2200us_2014-06"

0

dss_sc2 1 1 9.99999984307e+17 -9.99999984307e+17 0 4 1 0 1 64

1 0

0

dss_sc 1 1 9.99999984307e+17 -9.99999984307e+17 0 4 1 0 1 64

1 0

0

dss_wc 1 1 9.99999984307e+17 -9.99999984307e+17 0 4 1 0 1 64

1 251.75

0

dz 3 1 14 14 14 2 1 8192 1 64

1 1e-05

0

dutyc 1 1 9.99999984307e+17 -9.99999984307e+17 0 2 1 0 1 64

1 0.1

0

explabel 2 2 8 0 0 2 1 0 1 64

1 "gHSQC"

0

filter 7 1 1000000000 0 1 2 1 2 1 64

1 54.0999984741

0

f2SW 2 2 8 0 0 2 1 1 1 64

1 ""

0

f1SW 2 2 8 0 0 2 1 1 1 64

1 "setsw1(dn,110,39.99)"

0

exppath 2 2 8 0 0 2 1 0 1 64

1 "/home/vnmr1/vnmrsys/exp323/acqfil"

0

f1coef 2 2 8 0 0 3 1 1 1 64

1 "1 0 1 0 0 1 0 -1"

0

fadflg 4 2 4 0 0 2 1 0 1 64

1 "n"

0

faculty 2 2 8 0 0 2 1 0 1 64

1 ""

0

fb 7 1 6 6 6 2 1 8203 1 64

1 4000

0

fidid 2 2 8 0 0 2 1 0 1 64

1 ""

0

file 2 2 6 0 0 2 1 3 1 64

1 "/home/vnmrsys/data/PK/2014/v500/bio/2014-09-07_N15-UBI/05_N15-UBI_OVERNIGHT_NHsel_2014-09-08_v500_kp_rtPS-gHSQC_protein_31_d2o_10_25C_3ax2_03.fid"

0

fpmult1 1 1 1000000000 0 0 3 2 0 0 64

1 0.5

0

foldflg 4 2 4 0 0 2 1 0 1 64

1 "y"

0

fn 7 1 524288 64 -2 3 1 1 1 64

1 32768

0

filtfile 2 2 8 0 0 2 1 0 1 64

1 ""

0

fn1 7 1 524288 64 -2 3 1 1 1 64

1 512

0

fpmult 1 1 1000000000 0 0 3 1 0 0 64

1 1

0

gain 1 1 60 0 2 2 1 0 1 64

1 30

0

ftproc 1 1 9.99999984307e+17 -9.99999984307e+17 0 3 1 256 1 64

2 1 327

0

fzoom 7 1 32767 0 0 4 1 0 1 64

1 0

0

gf 1 1 100000 0 0 3 1 1 0 64

1 0.096

0

gf1 1 1 100000 0 0 3 1 1 1 64

1 0.032

0

gstab2 3 1 14 14 14 2 1 8192 1 64

1 0.0005

0

go_Options 2 2 8 0 0 2 1 7 1 64

1 "au"

0

gfs1 1 1 1000 -1000 0.001 3 1 1 0 64

1 0

0

gradaxis 2 2 8 0 0 2 1 0 1 64

1 "t"

0

go_id 2 2 8 0 0 2 1 7 1 64

1 "exp323.user.hostname_1410144742_203565"

0

gstab 3 1 14 14 14 2 1 8192 1 64

1 0.0002

0

gt2 3 1 14 14 14 2 1 8192 1 64

1 0.0005

0

gt0 3 1 14 14 14 2 1 8192 1 64

1 0.0016

0

gt1 3 1 14 14 14 2 1 8192 1 64

1 0.0005

0

gtE 3 1 14 14 14 2 1 8192 1 64

1 0.002

0

gtD 3 1 14 14 14 2 1 8192 1 64

1 0.0005

0

gzlvl1 1 1 1000000 -1000000 1 2 1 0 1 64

1 14160

0

gzlvl0 1 1 1000000 -1000000 1 2 1 0 1 64

1 0

0

gzlvl3 1 1 1000000 -1000000 1 2 1 0 1 64

1 -4096

0

gzlvl2 1 1 1000000 -1000000 1 2 1 0 1 64

1 11580

0

hdres 1 1 9.99999984307e+17 -9.99999984307e+17 0 2 1 0 1 64

1 9

0

hdfrqc 5 1 1000000000 -1000000000 0 2 1 256 0 64

1 0

0

gzlvlE 1 1 1000000 -1000000 1 2 1 0 1 64

1 16000

0

gzlvlD 1 1 1000000 -1000000 1 2 1 0 1 64

1 6485

0

gzlvlw 1 1 9.99999984307e+17 -9.99999984307e+17 0 2 1 0 1 64

1 14354

0

hdof 5 1 7 7 7 2 1 8194 1 64

1 -214.8

0

hdfrqw 1 1 9.99999984307e+17 -9.99999984307e+17 0 2 1 256 0 64

1 10

0

hdmf 1 1 9.99999984307e+17 -9.99999984307e+17 0 2 1 0 1 64

1 4301

0

hdpwr 7 1 49 -16 1 2 1 2 1 64

1 32

0

hdpsmode 4 2 4 0 0 2 1 0 1 64

1 "n"

0

hdpwrf 1 1 4095 0 0 2 1 0 1 64

1 4095

0

hs 4 2 4 0 0 2 1 0 1 64

1 "nn"

2 "n" "y"

homo 2 2 8 0 0 2 1 8 1 64

1 "n"

2 "n" "y"

hdseq 2 2 8 0 0 2 1 0 1 64

1 "kp_ubiHDEC_-2000Hz"

0

ho 1 1 500 -500 0 4 1 1 1 64

1 -2.43914473684

0

hsgt 3 1 14 14 14 2 1 8192 1 64

1 0.002

0

hsgradaxis 2 2 8 0 0 2 1 0 1 64

1 "z"

0

hsglvl 1 1 1000000 -1000000 1 2 1 0 1 64

1 13000

0

hsgstab 3 1 14 14 14 2 1 8192 1 64

1 0.0005

0

hzmm 1 1 1000000000 -1000000000 0 4 1 11 1 64

1 13.75

0

hst 3 1 0.02 0 2.5e-08 2 1 2 1 64

1 0.00800000037998

0

pw_XBIP 6 1 13 13 13 2 1 8192 1 64

1 188

0

n1 2 2 6 0 0 4 1 3 1 64

1 ""

0

llfrq 1 1 1000000000 -1000000000 0 4 1 0 0 64

3 2857.05566406 2838.43994141 2804.26025391

0

io 1 1 500 -500 0.1 4 1 1 1 64

1 10

0

intmod 2 2 8 0 0 4 1 0 1 64

1 "off"

3 "off" "partial" "full"

ins2ref 1 1 9.99999984307e+17 -9.99999984307e+17 0 4 1 0 0 64

1 1

0

ins 3 1 8190 0 1e-07 4 1 3 0 64

1 100

0

in 4 2 1 0 0 2 1 0 1 64

1 "nn"

3 "n" "w" "y"

ins2 1 1 9.99999984307e+17 -9.99999984307e+17 0 4 1 0 0 64

1 1

0

insref 1 1 9.99999984307e+17 -9.99999984307e+17 0 4 1 0 0 64

1 1

0

intstd 2 2 8 0 0 2 1 0 1 64

1 ""

0

invgcal 1 1 1000000 -1000000 1 2 1 0 1 64

1 0

0

iswt 1 1 9.99999984307e+17 -9.99999984307e+17 0 2 1 0 1 64

1 0

0

is 1 1 1000000000 1e-06 0 4 1 3 1 64

1 33.5734901428

0

j1xh 1 1 9.99999984307e+17 -9.99999984307e+17 0 2 1 0 1 64

1 90

0

kp_X180 7 1 32767 0 0 2 1 0 1 64

1 3

0

lb 1 1 100000 -100000 0 3 1 1 0 64

1 -4.96875

0

kp_save_psg 2 2 8 0 0 2 1 0 1 64

1 ""

0

kp_expinfo 2 2 8 0 0 2 1 0 1 64

1 ""

0

kp_save_maclib 2 2 8 0 0 2 1 0 1 64

1 ""

0

kpph 7 1 1024 -1024 1 2 1 0 1 64

1 0

0

layout 2 2 8 0 0 4 1 0 1 64

1 "gHSQC"

0

lifrq 1 1 1000000000 -1000000000 0 4 1 0 0 64

1 0

0

lcpeak_ 7 1 32767 0 0 2 1 32768 1 64

1 0

0

lb1 1 1 100000 -100000 0 3 1 1 0 64

1 -0.0162816

0

liamp 1 1 1000000000 0 0 4 1 0 0 64

1 0

0

llamp 1 1 1000000000 0 0 4 1 0 0 64

3 0.00836642086506 0.0101188952103 -0.00130691204686

0

lkgate_flg 4 2 4 0 0 2 1 0 1 64

1 "n"

0

lpalg 2 2 8 0 0 3 1 0 1 64

1 "lpfft"

0

lockpower_ 1 1 68 0 1 2 1 65552 1 64

1 30

0

locdir 2 2 8 0 0 2 1 0 1 64

1 ""

0

load 2 2 4 0 0 2 1 3 1 64

1 "n"

2 "y" "n"

lllabel 2 2 8 0 0 3 1 0 1 64

1 ""

0

loc_ 7 1 12 12 12 2 1 8201 1 64

1 0

0

lockgain_ 1 1 48 0 1 2 1 65552 1 64

1 43

0

lockfreq_ 5 1 160 0 1e-07 2 1 9 1 64

1 76.7263

0

lockphase_ 1 1 360 0 1 2 1 65552 1 64

1 307

0

lp 1 1 3600 -3600 0.1 4 1 3 1 64

1 0

0

lot 2 2 8 0 0 2 1 0 1 64

1 ""

0

longlist 2 2 8 0 0 2 1 0 1 64

1 ""

0

lp1 1 1 3600 -3600 0.1 4 1 3 1 64

1 0

0

lptrace1 7 1 32767 0 1 3 1 0 1 64

1 0

0

lpnupts1 7 1 1000000 1 1 3 1 0 1 64

1 48

0

lpfilt 7 1 32767 1 1 3 1 0 1 64

1 32

0

lpext 7 1 1000000 0 1 3 1 0 1 64

1 1

0

lpalg1 2 2 8 0 0 3 1 0 1 64

1 "lpfft"

0

lpext1 7 1 1000000 0 1 3 1 0 1 64

1 144

0

lpfilt1 7 1 32767 1 1 3 1 0 1 64

1 8

0

lpnupts 7 1 1000000 1 1 3 1 0 1 64

1 64

0

lpprint1 7 1 63 0 1 3 1 0 1 64

1 0

0

lpopt1 2 2 8 0 0 3 1 0 1 64

1 "f"

0

lpopt 2 2 8 0 0 3 1 0 1 64

1 "b"

0

lpprint 7 1 63 0 1 3 1 0 1 64

1 0

0

lptrace 7 1 32767 0 1 3 1 0 1 64

1 0

0

markerfrq1 1 1 9.99999984307e+17 -9.99999984307e+17 0 2 1 0 1 64

1 0

0

lvl 1 1 1000000000 -1000000000 0 4 1 1 1 64

1 0

0

lsfrq 1 1 9.99999984307e+17 -9.99999984307e+17 0 3 1 0 0 64

1 0

0

lsfid 7 1 64000 -64000 1 3 1 0 0 64

1 0

0

lsfrq1 1 1 9.99999984307e+17 -9.99999984307e+17 0 3 1 0 0 64

1 0

0

marker1 2 2 8 0 0 2 1 0 1 64

1 ""

0

macro 2 2 6 0 0 4 1 3 1 64

1 ""

0

marker2 2 2 8 0 0 2 1 0 1 64

1 ""

0

mfslp 4 2 4 0 0 2 1 0 1 64

1 "n"

0

math 2 2 1 0 0 3 1 3 1 64

1 "f"

3 "d" "f" "i"

markerfrq2 1 1 9.99999984307e+17 -9.99999984307e+17 0 2 1 0 1 64

1 0

0

method 2 2 6 0 0 2 1 3 1 64

1 "z1z2"

0

modules 2 2 8 0 0 2 1 1 1 64

1 "presat wet gradient quant par2D"

0

mult 1 1 9.99999984307e+17 -9.99999984307e+17 0 2 1 0 1 64

1 2

0

pipeistTMult 2 2 8 0 0 3 1 0 1 64

1 "0.7"

0

parlabel 2 2 8 0 0 2 1 5 1 64

1 "gHSQC"

0

obsSW 2 2 8 0 0 2 1 1 1 64

1 "setsw(14,-2)"

0

nameprefix 2 2 8 0 0 2 1 9 1 64

1 "N15-UBI_OVERNIGHT_NHsel"

0

n3 2 2 6 0 0 4 1 3 1 64

1 ""

0

n2 2 2 6 0 0 4 1 3 1 64

1 "n"

0

name 2 2 8 0 0 3 1 0 1 64

1 ""

0

ni 7 1 32767 0 0 2 1 0 1 64

1 64

0

null 3 1 14 14 14 2 1 8192 1 64

1 0

0

npoints 1 1 9.99999984307e+17 -9.99999984307e+17 0 2 1 0 1 64

1 320

0

np 7 1 524288 32 2 2 1 11 1 64

1 1920

0

nt 7 1 1000000000 1 1 2 1 2 1 64

1 32

0

numvch 7 1 32767 0 0 2 1 256 1 64

4 4 2 1 0

0

nullflg 4 2 4 0 0 2 1 0 1 64

1 "n"

0

oversamp 7 1 68 0 1 2 1 9 1 64

1 1

0

oscoef 7 1 49999 3 2 2 1 1 0 64

1 31

0

operator_ 2 2 128 0 0 2 1 4 1 64

1 "vnmr1"

0

osfilt 2 2 8 0 0 2 1 0 1 64

1 ""

5 "a" "A" "b" "B" ""

osfb 1 1 100000 0 0 2 1 1 0 64

1 0

0

oslsfrq 1 1 9.99999984307e+17 -9.99999984307e+17 0 2 1 1 0 64

1 0

0

pad 3 1 14 14 14 2 1 8194 1 64

1 0.5

0

p11console 2 2 8 0 0 2 1 0 1 64

1 ""

0

p1 6 1 13 13 13 2 1 8194 1 64

1 0

0

page_ 2 2 8 0 0 2 1 0 1 64

1 ""

0

page 2 2 8 0 0 2 1 0 1 64

1 ""

0

paramChanged 1 1 9.99999984307e+17 -9.99999984307e+17 0 2 1 0 1 64

1 0

0

pipeGLB 2 2 8 0 0 3 1 0 1 64

2 "4"

"4"

0

phase 7 1 8 0 1 2 1 0 1 64

2 1 2

0

parver 2 2 8 0 0 2 1 0 1 64

1 "VnmrJ VERSION 4.0 REVISION A"

0

parstyle_ 2 2 8 0 0 4 1 0 1 64

1 ""

0

parversion 1 1 9.99999984307e+17 -9.99999984307e+17 0 2 1 4 1 64

1 5.1

0

phfid1 1 1 3600 -3600 0.1 3 1 0 0 64

1 0

0

phfid 1 1 3600 -3600 0.1 3 1 0 1 64

1 0

0

pipeELB 2 2 8 0 0 3 1 0 1 64

2 "0"

"0"

0

pipeQ3 2 2 8 0 0 3 1 0 1 64

2 "0"

"0"

0

pipeQ1 2 2 8 0 0 3 1 0 1 64

2 "0"

"0"

0

pipeGOFF 2 2 8 0 0 3 1 0 1 64

2 "0"

"0"

0

pipeQ2 2 2 8 0 0 3 1 0 1 64

2 "0"

"0"

0

pipeistIter 2 2 8 0 0 3 1 0 1 64

1 "1024"

0

pipeistCMult 2 2 8 0 0 3 1 0 1 64

1 "0.3"

0

pipeistMaxRes 2 2 8 0 0 3 1 0 1 64

1 "1"

0

prggt 3 1 14 14 14 2 1 8192 1 64

1 0.001

0

presatgh2 1 1 9.99999984307e+17 -9.99999984307e+17 0 2 1 0 1 64

1 10

0

pmode 2 2 8 0 0 3 1 0 1 64

1 "full"

0

pltmod 2 2 8 0 0 4 1 0 1 64

1 "fixed"

5 "off" "fixed" "full" "variable" "user"

plocklist 2 2 8 0 0 2 1 1 1 64

1 ""

0

pkpick_ 2 2 8 0 0 4 1 0 1 64

1 ""

0

plt2Darg 2 2 8 0 0 4 1 272 1 64

8 "both"

"20"

"1.3"

"f2"

"f1"

""

""

""

0

pltopt 4 2 4 0 0 2 1 0 1 64

1 "n"

0

pplvl 1 1 63 -16 1 2 1 0 1 64

1 55

0

position2 2 2 8 0 0 3 1 0 1 64

1 ""

0

position1 2 2 8 0 0 3 1 0 1 64

1 ""

0

pp 6 1 13 13 13 2 1 8192 1 64

1 15

0

prcopt 4 2 4 0 0 2 1 0 1 64

1 "y"

0

pplvl_cf 1 1 9.99999984307e+17 -9.99999984307e+17 0 2 1 0 1 64

1 1

0

preacq 6 1 13 13 13 2 1 8192 0 64

1 0

0

prgd1 3 1 14 14 14 2 1 8192 1 64

1 0.0002

0

prescan 2 2 8 0 0 3 1 256 1 64

11 "Not done"

"Not done"

"off"

"off"

"off"

"off"

"off"

"on"

"on"

""

""

0

presatinit 4 2 4 0 0 2 1 0 1 64

1 "n"

0

proccmd 2 2 8 0 0 3 1 7 1 64

1 "wft2d('noop','noop','noop','noop','noop','1','0','1','0','0','1','0','-1')"

0

probe_ 2 2 8 0 0 2 1 8 1 64

1 "3ax2"

0

priority 7 1 32768 0 1 2 1 0 1 64

1 5

0

proc 2 2 10 0 0 3 1 1 1 64

1 "ft"

0

probetype_ 2 2 8 0 0 2 1 1 1 64

1 "liquids"

0

proc1 2 2 10 0 0 3 2 1 1 64

1 "ft"

0

processid 1 1 9.99999984307e+17 -9.99999984307e+17 0 3 1 0 1 64

1 0

0

procdim 7 1 32767 0 0 3 1 0 1 64

1 2

0

protocols 2 2 8 0 0 3 1 0 1 64

1 ""

0

pspreacq 2 2 8 0 0 2 1 256 1 64

1 "auto"

0

pslock 2 2 8 0 0 2 1 256 1 64

1 "auto"

0

psgshim 2 2 8 0 0 2 1 256 1 64

1 "auto"

0

psgain 2 2 8 0 0 2 1 256 1 64

1 "auto"

0

pslabel 2 2 8 0 0 2 1 11 1 64

1 "kp_rtPS-gHSQC_protein_31"

0

psppm 2 2 8 0 0 2 1 1 1 64

1 ""

0

pw90 6 1 13 13 13 2 1 8194 1 64

1 19.6

0

pstune 2 2 8 0 0 2 1 256 1 64

1 "auto"

0

pstof 5 1 1000000000 -1000000000 0 2 1 264 1 64

1 0

0

pw 6 1 13 13 13 2 1 8194 1 64

1 9.6

0

pw_HBIP 6 1 13 13 13 2 1 8192 1 64

1 19.2

0

pw_H2O 6 1 13 13 13 2 1 8192 1 64

1 1400

0

pw_NH 6 1 13 13 13 2 1 8192 1 64

1 2220

0

strtlp1 7 1 1000000 1 1 3 1 0 1 64

1 48

0

samplename 2 2 8 0 0 2 1 9 1 64

1 "N15-UBI_OVERNIGHT_NHsel"

0

r3 1 1 1e+18 -1e+18 0 4 1 1 1 64

1 0

0

pwx180adR 2 2 8 0 0 2 1 0 1 64

1 "kp_N15_wu2i-5kHz_400usR"

0

pwr_NH 1 1 9.99999984307e+17 -9.99999984307e+17 0 2 1 0 1 64

1 42

0

pwr_H2O 1 1 9.99999984307e+17 -9.99999984307e+17 0 2 1 0 1 64

1 20

0

pwr_HBIP 1 1 9.99999984307e+17 -9.99999984307e+17 0 2 1 0 1 64

1 61

0

pwr_XBIP 1 1 9.99999984307e+17 -9.99999984307e+17 0 2 1 0 1 64

1 58

0

pwx180 6 1 13 13 13 2 1 8192 1 64

1 400.9

0

pwx 6 1 13 13 13 2 1 8192 1 64

1 47

0

pwx180ad 2 2 8 0 0 2 1 0 1 64

1 "kp_N15_wu2i-5kHz_400us"

0

pwxlvl180r 1 1 9.99999984307e+17 -9.99999984307e+17 0 2 1 0 1 64

1 0

0

pwx180r 6 1 13 13 13 2 1 8192 1 64

1 0

0

pwxlvl 1 1 63 -16 1 2 1 0 1 64

1 58

0

pwxlvl180 1 1 9.99999984307e+17 -9.99999984307e+17 0 2 1 0 1 64

1 58

0

r1 1 1 1e+18 -1e+18 0 4 1 1 1 64

1 62

0

pwxlvlS6 1 1 9 9 9 2 1 8192 1 64

1 60

0

pwxlvl_cf 1 1 9.99999984307e+17 -9.99999984307e+17 0 2 1 0 1 64

1 1.029

0

r2 1 1 1e+18 -1e+18 0 4 1 1 1 64

1 111

0

refpos1 5 1 1000000000 -1000000000 0 3 1 0 1 64

1 0

0

ref 5 1 1000000000 -1000000000 0 3 4 0 1 64

1 0

0

r6 1 1 1e+18 -1e+18 0 4 1 1 1 64

1 0

0

r4 1 1 1e+18 -1e+18 0 4 1 1 1 64

1 0.032

0

r5 1 1 1e+18 -1e+18 0 4 1 1 1 64

1 0

0

r7 1 1 1e+18 -1e+18 0 4 1 1 1 64

1 1

0

ref_pwr 1 1 9.99999984307e+17 -9.99999984307e+17 0 2 1 0 1 64

1 61

0

ref1 5 1 1000000000 -1000000000 0 3 1 0 1 64

1 0

0

ref_pw90 6 1 13 13 13 2 1 8192 1 64

1 9.6

0

reffrq1 1 1 9.99999984307e+17 -9.99999984307e+17 0 4 1 0 1 64

1 50.6466710947

0

reffrq 1 1 9.99999984307e+17 -9.99999984307e+17 0 4 1 0 1 64

1 499.823455813

0

refpos 1 1 9.99999984307e+17 -9.99999984307e+17 0 4 1 0 1 64

1 -1.59696708124

0

rfp1 1 1 1000000000 -1000000000 0 4 1 1 1 64

1 0

0

rfband 4 2 4 0 0 2 1 0 1 64

1 "ccc"

3 "c" "h" "l"

refsource1 2 2 8 0 0 4 1 0 1 64

1 "dfrq"

0

retentiontime_ 2 2 8 0 0 2 1 0 1 64

1 ""

0

rfl1 1 1 1000000000 -1000000000 0 4 1 1 1 64

1 -4953.60527364

0

rfl 1 1 1000000000 -1000000000 0 4 1 1 1 64

1 2482.90597574

0

rfp 1 1 1000000000 -1000000000 0 4 1 1 1 64

1 3983.5929428

0

rp 1 1 3600 -3600 0.1 4 1 3 1 64

1 141.7

0

rof2 6 1 13 13 13 2 1 8194 1 64

1 24.8

0

rof1 6 1 13 13 13 2 1 8194 1 64

1 10

0

rof3 6 1 13 13 13 2 1 8192 1 64

1 0

0

sa 7 1 10000000 0 0 3 1 1 0 64

1 1024

0

rp1 1 1 3600 -3600 0.1 4 1 3 1 64

1 0

0

sample 2 2 8 0 0 2 1 1 1 64

1 ""

0

showarray 2 2 8 0 0 4 1 0 1 64

1 "horiz"

0

sbs1 1 1 1000 -1000 0.001 3 1 1 0 64

1 0

0

sampwt 1 1 9.99999984307e+17 -9.99999984307e+17 0 2 1 0 1 64

1 0

0

samplename_global_ 2 2 8 0 0 2 1 0 1 64

1 ""

0

sas 7 1 10000000 0 0 3 1 1 0 64

1 0

0

sb 1 1 1000 -1000 0.001 3 1 0 0 64

1 0

0

saveglobal_ 2 2 8 0 0 3 1 0 1 64

24 "probe"

"lcpeak"

"loc"

"lockpower"

"lockgain"

"lockphase"

"lockfreq"

"z0"

"lkof"

"vloc"

"vrack"

"vzone"

"vproto"

"pkpick"

"parstyle"

"operator"

"studyid"

"systemname"

"probetype"

"investigator"

"notebook"

"page"

"study"

"samplename_global"

0

satpwr 1 1 30 -16 1 2 1 0 1 64

1 0

0

sbbiflg 4 2 4 0 0 2 1 0 1 64

1 "n"

0

sb1 1 1 1000 -1000 0.001 3 1 1 0 64

1 0

0

sbs 1 1 1000 -1000 0.001 3 1 1 0 64

1 0

0

segt5 3 1 14 14 14 2 1 8192 1 64

1 0.0005

0

sc2 1 1 3 3 3 4 1 8195 1 64

1 0

0

sc 1 1 1 1 1 4 1 8195 1 64

1 0

0

scans 2 2 8 0 0 3 1 0 1 64

1 ""

0

segstab 3 1 14 14 14 2 1 8192 1 64

1 0.0005

0

sdirtmplt 2 2 8 0 0 2 1 0 1 64

1 "$samplename$_%DATE%_"

0

sf 1 1 8190 0 1e-07 4 1 3 1 64

1 0

0

seqfil 2 2 8 0 0 2 1 11 1 64

1 "kp_rtPS-gHSQC_protein_31"

0

selfrq 5 1 1000000000 -1000000000 0 2 1 0 1 64

1 1500

0

sfrq 1 1 1000000000 0 0 2 1 11 1 64

1 499.8274565

0

sf1 1 1 9.99999984307e+17 -9.99999984307e+17 0 4 1 0 1 64

1 0

0

shims 2 2 8 0 0 2 1 0 1 64

1 ""

0

sn 1 1 1e+18 -1e+18 0 3 1 1 1 64

1 0

0

shp_NH 2 2 8 0 0 2 1 0 1 64

1 "kp_ubi_reburb-2200"

0

shownumx 1 1 9.99999984307e+17 -9.99999984307e+17 0 4 1 0 1 64

1 0

0

shownumbers 2 2 8 0 0 4 1 0 1 64

1 "n"

0

showcontent 2 2 8 0 0 4 1 0 1 64

1 "s"

0

shownumstyle 2 2 8 0 0 4 1 0 1 64

1 "auto"

0

shp_H2O 2 2 8 0 0 2 1 0 1 64

1 "H2O_sinc-1kHz"

0

shownumy 1 1 9.99999984307e+17 -9.99999984307e+17 0 4 1 0 1 64

1 0

0

shp_HBIP 2 2 8 0 0 2 1 0 1 64

1 ""

0

shp_XBIP 2 2 8 0 0 2 1 0 1 64

1 "kp_N15_bip50us"

0

shp_NHR 2 2 8 0 0 2 1 0 1 64

1 ""

0

ssfilter 1 1 50000 10 0.1 3 1 0 0 64

1 100

0

spin 1 1 39 0 1 2 1 0 0 64

1 0

0

sp 1 1 1000000000 -1000000000 0 4 1 3 1 64

1 4381.54634206

0

solvent 2 2 6 0 0 2 1 11 1 64

1 "d2o_10"

0

sp1 1 1 1000000000 -1000000000 0 4 1 3 1 64

1 6375.48027364

0

sreffrq 1 1 9.99999984307e+17 -9.99999984307e+17 0 2 1 0 1 64

1 499.82345741

0

srate 7 1 32767 0 1 2 1 1 1 64

1 25

0

ss 7 1 32767 -32768 0 2 1 0 1 64

1 256

0

sspul 4 2 4 0 0 2 1 0 1 64

1 "y"

0

ssntaps 7 1 2500 0 1 3 1 0 1 64

1 121

0

sslsfrq 1 1 9.99999984307e+17 -9.99999984307e+17 0 3 1 0 0 64

1 0

0

ssorder 7 1 20 1 1 3 1 0 0 64

1 7

0

strtext1 7 1 1000000 1 1 3 1 0 1 64

1 49

0

strtext 7 1 1000000 1 1 3 1 0 1 64

1 1

0

strtlp 7 1 1000000 1 1 3 1 0 1 64

1 2

0

wetpwr1 1 1 9.99999984307e+17 -9.99999984307e+17 0 2 1 0 1 64

1 0

0

vjdialogarg 2 2 8 0 0 4 1 256 1 64

1 ""

0

time_run 2 2 8 0 0 2 1 0 1 64

1 "20140908T035223"

0

t2 3 1 14 14 14 2 1 8192 1 64

1 0

0

studystatus 2 2 8 0 0 2 1 0 1 64

1 ""

0

studyid_ 2 2 8 0 0 2 1 0 1 64

1 "tmpstudy"

0

studyowner 2 2 8 0 0 2 1 0 1 64

1 "peter"

0

sw 1 1 5 5 5 2 1 8203 1 64

1 5000

0

svfopt 4 2 4 0 0 2 1 0 1 64

1 "y"

0

svftmplt 2 2 8 0 0 2 1 0 1 64

1 "$pslabel$_"

0

systemname_ 2 2 8 0 0 2 1 0 1 64

1 "hostname-vnmrs500"

0

sw1 1 1 5000000 1 -1.25e-08 2 1 0 1 64

1 2000

0

t1 3 1 14 14 14 2 1 8192 1 64

1 0

0

text_string 2 2 8 0 0 4 1 256 1 64

1 ""

0

tauA 6 1 13 13 13 2 1 8192 1 64

1 20

0

tBal 3 1 14 14 14 2 1 8192 1 64

1 0

0

t3 3 1 14 14 14 2 1 8192 1 64

1 0

0

tauC 6 1 13 13 13 2 1 8192 1 64

1 10

0

tauB 6 1 13 13 13 2 1 8192 1 64

1 10

0

temp 1 1 200 -150 0.1 2 1 8 1 64

1 25

0

time_complete 2 2 8 0 0 4 1 0 1 64

1 "20140908T075342"

0

th2d 1 1 1 1e-18 0 4 1 17 1 64

1 0.8

0

th 1 1 1000000000 0 0 4 1 1 1 64

1 2

0

time_plotted 2 2 8 0 0 3 1 0 1 64

1 "20140930T174033"

0

time_exp 2 2 8 0 0 2 1 0 1 64

1 "2 min, 12 sec"

0

time_processed 2 2 8 0 0 3 1 0 1 64

1 "20140930T172828"

0

tpwr_cf 1 1 9.99999984307e+17 -9.99999984307e+17 0 2 1 0 1 64

1 1

0

time_svfdate 2 2 8 0 0 2 1 0 1 64

1 "20140908T035222"

0

time_submitted 2 2 8 0 0 2 1 0 1 64

1 "20140908T035222"

0

time_saved 2 2 8 0 0 4 1 0 1 64

1 "20141218T181304"

0

time_submitted_local 2 2 8 0 0 2 1 0 1 64

1 "20140908T035222X03AMMonSep"

0

tlt 1 1 1000000000 -1000000000 0 4 1 1 1 64

1 0

0

tin 2 2 1 0 0 2 1 2 1 64

1 "n"

3 "n" "w" "y"

tn 2 2 4 0 0 2 1 8 1 64

1 "H1"

0

tnref 2 2 8 0 0 4 1 1 1 64

1 "Me4Si, 1% in CDCl3"

0

tofh2o 5 1 1000000000 -1000000000 0 2 1 0 1 64

1 0

0

tof 5 1 7 7 7 2 1 8202 1 64

1 1500

0

tpwr 1 1 17 17 17 2 1 8194 1 64

1 61

0

vch3 2 2 8 0 0 2 1 8 1 64

1 "z"

0

username 2 2 8 0 0 2 1 0 1 64

1 ""

0

tunemethod 2 2 8 0 0 2 1 0 1 64

1 "n"

0

trace 2 2 2 0 0 4 1 3 1 64

1 "f2"

2 "f1" "f2"

vch1 2 2 8 0 0 2 1 8 1 64

1 "tn"

0

userplotter 2 2 8 0 0 4 1 0 1 64

1 ""

0

vch2 2 2 8 0 0 2 1 8 1 64

1 "dn"

0

vchannelmap 2 2 8 0 0 2 1 256 1 64

4 "0"

"1"

"z"

"1"

0

vch5 2 2 8 0 0 2 1 8 1 64

1 ""

0

vch4 2 2 8 0 0 2 1 8 1 64

1 "dn"

0

vch6 2 2 8 0 0 2 1 8 1 64

1 ""

0

vjdialog 2 2 8 0 0 4 1 256 1 64

1 ""

0

vf 1 1 1000000000 0 0 4 1 3 1 64

1 250685

0

vzone_ 7 1 15 1 0 2 1 0 1 64

1 1

0

vrack_ 7 1 5 1 0 2 1 0 1 64

1 1

0

vp 7 1 500 -500 0.1 4 1 3 1 64

1 20

0

vo 1 1 500 -500 0 4 1 1 1 64

1 0

0

vloc_ 2 2 8 0 0 2 1 8 1 64

1 "A0"

0

volume 1 1 9.99999984307e+17 -9.99999984307e+17 0 2 1 0 1 64

1 0

0

vpfi 1 1 500 -500 0 4 1 0 1 64

1 0

0

vpf 1 1 500 -500 0 4 1 0 1 64

1 0

0

vproto_ 2 2 8 0 0 2 1 0 1 64

1 ""

0

vsproj 1 1 1000000000 1e-06 0 4 1 3 1 64

1 4.45071601868

0

vs2d 1 1 1000000000 1e-06 0 4 1 3 1 64

1 240

0

vs 1 1 1000000000 1e-06 0 4 1 3 1 64

1 1681.7936437

0

vtwait 3 1 14 14 14 2 1 8194 1 64

1 300

0

vtc 1 1 100 -200 1 2 1 2 1 64

1 25

0

wc2 1 1 4 4 4 4 1 8195 1 64

1 120

0

wbs 2 2 6 0 0 3 1 3 1 64

1 " "

0

wc 1 1 2 2 2 4 1 8203 1 64

1 160

0

wds 2 2 8 0 0 3 1 0 1 64

1 ""

0

wdone 2 2 8 0 0 3 1 0 1 64

1 ""

0

werr 2 2 6 0 0 3 1 3 1 64

1 " "

0

xz 7 1 19 19 19 2 1 8192 1 64

1 -9004

0

wnt 2 2 6 0 0 3 1 3 1 64

1 ""

0

wf1 1 1 9.99999984307e+17 -9.99999984307e+17 0 4 1 0 1 64

1 0.128

0

wexp 2 2 6 0 0 3 1 3 1 64

1 "wft2da setref setref1(dn) kp_save kp_nextexp au"

0

wf 1 1 8190 0 1e-07 4 1 3 1 64

1 0.192

0

whatemail 2 2 8 0 0 2 1 0 1 64

1 "message"

0

whenemail 2 2 8 0 0 2 1 0 1 64

1 "never"

0

wtfile1 2 2 15 0 0 3 2 1 1 64

1 ""

0

wrtp 2 2 8 0 0 3 1 0 1 64

1 ""

0

wp1 1 1 100000 0 0 4 1 3 1 64

1 140.625

0

wp 1 1 5000000 0 0 4 1 11 1 64

1 396.728515625

0

wqinit 2 2 8 0 0 4 1 0 1 64

1 ""

0

wstart 2 2 8 0 0 3 1 0 1 64

1 ""

0

wshim 4 2 4 0 0 2 1 1 1 64

1 "n"

15 "e" "f" "g" "n" "s" "0" "1" "2" "3" "4" "5" "6" "7" "8" "9"

wtfile 2 2 15 0 0 3 1 1 1 64

1 ""

0

x3 7 1 19 19 19 2 1 8192 1 64

1 3806

0

x1 7 1 19 19 19 2 1 8192 1 64

1 2998

0

wtune 4 2 4 0 0 2 1 0 1 64

1 "n"

10 "s" "t" "e" "v" "i" "n" "1" "2" "3" "4"

x2y2 7 1 19 19 19 2 1 8192 1 64

1 4775

0

xdiag 1 1 1e+18 0 0 4 1 17 1 64

1 0

0

x4 7 1 19 19 19 2 1 8192 1 64

1 -10344

0

xy 7 1 19 19 19 2 1 8192 1 64

1 -4866

0

z3y3 7 1 19 19 19 2 1 8192 1 64

1 -1106

0

z3 7 1 19 19 19 2 1 8192 1 64

1 1385

0

z1 7 1 19 19 19 2 1 8192 1 64

1 1730

0

y4 7 1 19 19 19 2 1 8192 1 64

1 0

0

y1 7 1 19 19 19 2 1 8192 1 64

1 -885

0

xz2 7 1 19 19 19 2 1 8192 1 64

1 -4808

0

y3 7 1 19 19 19 2 1 8192 1 64

1 1879

0

yz2 7 1 19 19 19 2 1 8192 1 64

1 5641

0

yz 7 1 19 19 19 2 1 8192 1 64

1 3467

0

z0_ 1 1 19 19 19 2 1 73744 1 64

1 -327

0

z2x2y2 7 1 19 19 19 2 1 8192 1 64

1 69

0

z2 7 1 19 19 19 2 1 8192 1 64

1 -442

0

z1c 7 1 19 19 19 2 1 8192 1 64

1 -1044

0

z2c 7 1 19 19 19 2 1 8192 1 64

1 196

0

z2xy 7 1 19 19 19 2 1 8192 1 64

1 8024

0

z2x3 7 1 19 19 19 2 1 8192 1 64

1 -1688

0

z2y3 7 1 19 19 19 2 1 8192 1 64

1 2262

0

z3x2y2 7 1 19 19 19 2 1 8192 1 64

1 6768

0

z3x 7 1 19 19 19 2 1 8192 1 64

1 -3003

0

z3c 7 1 19 19 19 2 1 8192 1 64

1 0

0

z3xy 7 1 19 19 19 2 1 8192 1 64

1 9646

0

z3x3 7 1 19 19 19 2 1 8192 1 64

1 -10368

0

z3y 7 1 19 19 19 2 1 8192 1 64

1 -6697

0

z5x 7 1 19 19 19 2 1 8192 1 64

1 10371

0

z4x2y2 7 1 19 19 19 2 1 8192 1 64

1 -6355

0

z4c 7 1 19 19 19 2 1 8192 1 64

1 0

0

z4 7 1 19 19 19 2 1 8192 1 64

1 6179

0

z4x 7 1 19 19 19 2 1 8192 1 64

1 1084

0

z4y 7 1 19 19 19 2 1 8192 1 64

1 3714

0

z4xy 7 1 19 19 19 2 1 8192 1 64

1 0

0

z5 7 1 19 19 19 2 1 8192 1 64

1 563

0

z8 7 1 19 19 19 2 1 8192 1 64

1 0

0

z6 7 1 19 19 19 2 1 8192 1 64

1 9928

0

z5y 7 1 19 19 19 2 1 8192 1 64

1 0

0

z7 7 1 19 19 19 2 1 8192 1 64

1 861

0

zx2y2 7 1 19 19 19 2 1 8192 1 64

1 1687

0

zoom 7 1 32767 0 0 4 1 0 1 64

1 0

0

zxy 7 1 19 19 19 2 1 8192 1 64

1 5009

0

zx3 7 1 19 19 19 2 1 8192 1 64

1 3240

0

zy3 7 1 19 19 19 2 1 8192 1 64

1 -9619

0

**Pulse sequence code for Bruker spectrometers**

;ek_ti_rakp_fbhsqcf3_wg2

;pure shift HSQC with real-time acquisition on F3 channel

;using explicit acquisition dwellmode

;using digital mode acquisition

;avance-version - Avance II, AQS system, SADC digitizer, RX22 receiver, TopSpin 2.1

;based on hsqcetgpsi2

;using sensitivity improvement

;2D H-1/X correlation via double inept transfer

;phase sensitive using Echo/Antiecho-TPPI gradient selection

;with decoupling during acquisition

;using trim pulse in inept transfer

;June 26, 2014

;$CLASS=HighRes

;$DIM=2D

;$TYPE=

;$SUBTYPE=

;$COMMENT=

#include <Avance.incl>

#include <Grad.incl>

#include <Delay.incl>

#include <De.incl>

define loopcounter count

define delay dwell

"p2=p1*2"

"p22=p21*2"

"d4=1s/(cnst2*4)"

"d20=1s/(2*cnst2)"

"d11=30m"

"d13=4u"

"d0=3u"

"in0=inf1/2"

"l3=(td1/2)"

"DELTA1=d13+p16+d16+4u"

"DELTA2=d24-p16-d16"

"DELTA3=d4-p16-d16"

# ifdef LABEL_CN

"DELTA=p16+d16+50u+larger(p2,p22)+d0*2"

# else

"DELTA=50u+p2+d0*2"

# endif /*LABEL_CN*/

"d19=dw*l12"

"l11=(0.5*decim*l12/2)"

"l13=(l12*decim*0.5)"

"count=(aq/d19)-1"

"dwell=(dw/decim)"

;dwellmode explicit

1 ze

d11 pl16:f3

2 d1 do:f3

6m pl1:f1

3 d11 pl1:f1

9m

4 (p1 ph1)

d4 pl3:f3

(center (p2 ph1) (p22 ph6):f3 )

d4

p28 ph1

d13

(p1 ph2) (p21 ph3):f3

d0

# ifdef LABEL_CN

(center (p2 ph7) (p22 ph1):f3 )

# else

(p2 ph7)

# endif /*LABEL_CN*/

d0

50u UNBLKGRAD

p16:gp1*1*EA

d16

(p22 ph4):f3

p16:gp1*-1*EA

d16

DELTA

(center (p1 ph1) (p21 ph4):f3 )

p16:gp3

d16

DELTA2

(center (p2 ph1) (p22 ph1):f3 )

DELTA2

p16:gp3

d16

(center (p1 ph2) (p21 ph5):f3 )

p16:gp4

d16

DELTA3

(center (p2 ph1) (p22 ph1):f3 )

DELTA3

p16:gp4

d16

(p1 ph1)

DELTA1

(p2 ph1)

d13

p16:gp9

d16 pl16:f3

4u BLKGRAD

5 ACQ_START(ph30,ph31) ;takes de

;;initial half chunk

5u pl16:f3

5u cpd3:f3

10u REC_UNBLK

10u syrec

6 dwell DWL_CLK_ON

dwell DWL_CLK_OFF

lo to 6 times l11

10u REC_BLK

10u sytra

5u do:f3

5u UNBLKGRAD

p17:gp12 ;gradient pulse for coherence selection and water suppression during acquisition

d16

5u

(p1 ph8)

d20 pl3:f3

(center (p2 ph9) (p22 ph9):f3 )

d20 pl1:f1

(ralign (p1 ph10) (p22 ph9):f3 )

5u

p17:gp12 ;gradient pulse for coherence selection and water suppression during acquisition

d16

60u

p19:gp13 ;gradient pulse for coherence selection and water suppression during acquisition

d16

5u

d31

(p2 ph18)

d31

5u

p19:gp13 ;gradient pulse for coherence selection and water suppression during acquisition

d16 BLKGRAD

;;looped chunks

7 5u pl16:f3

5u cpd3:f3

10u REC_UNBLK

10u syrec

8 dwell DWL_CLK_ON

dwell DWL_CLK_OFF

lo to 8 times l13

10u REC_BLK

10u sytra

5u do:f3

5u UNBLKGRAD

p17:gp12 ;gradient pulse for coherence selection and water suppression during acquisition

d16

5u

(p1 ph8)

d20 pl3:f3

(center (p2 ph9) (p22 ph9):f3 )

d20 pl1:f1

(ralign (p1 ph10) (p22 ph9):f3 )

5u

p17:gp12 ;gradient pulse for coherence selection and water suppression during acquisition

d16

60u

p19:gp13 ;gradient pulse for coherence selection and water suppression during acquisition

d16

5u

d31

(p2 ph18)

d31

5u

p19:gp13 ;gradient pulse for coherence selection and water suppression during acquisition

d16 BLKGRAD

lo to 7 times count

;;final half chunk

5u pl16:f3

5u cpd3:f3

10u REC_UNBLK

10u syrec

9 dwell DWL_CLK_ON

dwell DWL_CLK_OFF

lo to 9 times l11

10u REC_BLK

10u sytra

5u do:f3

5u

50u

rcyc=2

10 d1 do:f3 wr #0 if #0 zd

3m igrad EA

3m ip5*2

lo to 3 times 2

d11 id0

3m ip3*2

3m ip6*2

3m ip31*2

lo to 4 times l3

11 exit

;Phase Cycling

ph1=0

ph2=1

ph3=0 2

ph4=0 0 0 0 2 2 2 2

ph5=1 1 1 1 3 3 3 3

ph6=0

ph7=0 0 0 0 2 2 2 2

ph8= 0 0 1 1

ph9= 1 1 2 2

ph10=2 2 3 3

ph18=0 0 1 1

ph29=0

ph30=0

ph31=0 2 0 2 2 0 2 0

;pl1 : f1 channel - power level for pulse (default)

;pl2 : f2 channel - power level for pulse (default)

;pl3 : f3 channel - power level for pulse (default)

;pl12: f2 channel - power level for CPD/BB decoupling

;pl16: f3 channel - power level for CPD/BB decoupling

;pl0: pulse power off [120 dB]

;p1 : f1 channel - 90 degree high power pulse

;p2 : f1 channel - 180 degree high power pulse

;p3 : f2 channel - 90 degree high power pulse

;p4 : f2 channel - 180 degree high power pulse

;p21 : f3 channel - 90 degree high power pulse

;p22 : f3 channel - 180 degree high power pulse

;p16: gradient t1 encoding/purging gradient pulse [1 ms]

;p17: gradient pulse [0.5 ms]

;p19: gradient pulse [0.5 ms]

;p28: f1 channel - trim pulse

;d0 : incremented delay (2D) [3 usec]

;d1 : relaxation delay; 1-5 * T1

;d4 : 1/(4J)XH

;d24: 1/(4J)XH for XH

; 1/(8J)XH for all multiplicities

;d20: BIRD delay - 1/(2J)XH

;d11: delay for disk I/O [30 msec]

;d13: short delay [4 usec]

;d31: short delay [10 usec]

;d16: delay for homospoil/gradient recovery

;cnst2: = J(XH)

;inf1: 1/SW(X) = 2 * DW(X)

;in0: 1/(2 * SW(X)) = DW(X)

;nd0: 2

;NS: 4 * n

;DS: >= 16

;td1: number of experiments

;FnMODE: echo-antiecho

;cpd3: decoupling according to sequence defined by cpdprg3

;pcpd3: f3 channel - 90 degree pulse for decoupling sequence

;use gradient ratio: gp 1 : gp 9

; 80 : 40.2 for C-13

; 80 : 16.2 for N-15

;for z-only gradients:

;gpz3: homospoil 42%

;gpz4: homospoil 67%

;gpz12: ctp 61%

;gpz13: ctp 31%

;use gradient files:

;gpnam1: SINE.100

;gpnam3: SINE.100

;gpnam4: SINE.100

;gpnam9: SINE.100

;gpnam12: SINE.100

;gpnam13: SINE.100

;preprocessor-flags-start

;LABEL_CN: for C-13 and N-15 labeled samples start experiment with

;option -DLABEL_CN (eda: ZGOPTNS)

;preprocessor-flags-end

;$Id: hsqcetgp,v 1.4 2007/04/11 13:34:30 ber Exp $
